# Real-world effectiveness of CDK4/6i in first-line treatment of HR+/HER2− advanced/metastatic breast cancer: updated systematic review

**DOI:** 10.3389/fonc.2025.1530391

**Published:** 2025-03-10

**Authors:** Nadia Harbeck, Adam Brufsky, Chloe Grace Rose, Beata Korytowsky, Connie Chen, Krista Tantakoun, Endri Jazexhi, Do Hoang Vien Nguyen, Meaghan Bartlett, Imtiaz A. Samjoo, Timothy Pluard

**Affiliations:** ^1^ Breast Center, Department of Gynecology and Obstetrics and Comprehensive Cancer Center Munich, LMU University Hospital, Munich, Germany; ^2^ UPMC Hillman Cancer Center, University of Pittsburgh Medical Center, Pittsburgh, PA, United States; ^3^ Pfizer, Inc., New York, NY, United States; ^4^ Value & Evidence, EVERSANA^TM^ , Burlington, ON, Canada; ^5^ Hematology and Medical Oncology, St. Luke's Cancer Institute, Kansas City, MO, United States

**Keywords:** CDK4/6 inhibitors, quality assessment, breast, metastasis, real-world evidence, systematic literature review, HR+/HER2−

## Abstract

**Aim:**

Since 2021, additional real-world evidence (RWE) has emerged on the effectiveness of cyclin-dependent kinase 4/6 inhibitors (CDK4/6i) as first-line treatment of HR-positive/HER2-negative (HR+/HER2−) advanced/metastatic breast cancer (A/MBC), necessitating this updated review.

**Methods:**

MEDLINE^®^, Embase^®^, and Cochrane Databases (07/06/2019–01/09/2024), and key congresses (2020–2024) were searched. Studies reporting first-line CDK4/6i use, over 100 participants, and progression-free survival (PFS) and/or overall survival (OS) data were included.

**Results:**

This update included 82 unique studies, 42.7% for palbociclib, 7.3% for ribociclib, and 3.7% for abemaciclib; 46.3% assessed multiple CDK4/6i. In studies including multiple CDK4/6is, median PFS was 23.4–31.0 months for palbociclib, 19.8–44.0 for ribociclib, and 14.0–39.5 for abemaciclib. When reached, median OS was 38.0–58.0 months, 40.4–52.0 months, and 34.4 months, respectively. These real-world PFS and OS results were within the range of single-arm and CDK4/6i versus endocrine therapy (ET) studies, where CDK4/6i demonstrated greater benefits than ET alone.

**Conclusion:**

First-line CDK4/6i RWE demonstrates significant clinical benefits in HR+/HER2− A/MBC. These data are important to guide clinical decision-making, as they include patients who are not adequately represented in clinical trials. Studies with longer follow-up are needed to assess long-term benefits of all three CDK4/6i therapies in HR+/HER2− A/MBC.

## 1 Introduction

Breast cancer (BC) is the most commonly diagnosed cancer and the leading cause of cancer deaths in women globally (1). According to GLOBOCAN, approximately 2.0 million new cases of BC were diagnosed in 2022 worldwide, accounting for 11.5% of all new cancer cases and 6.8% of all cancer-related deaths (2).

The disease stage and subtype at diagnosis strongly influence survival with BC. Based on Surveillance, Epidemiology, and End Results (SEER) data from 2014–2020, 28% of patients were diagnosed with regional stage BC (i.e., cancer that has spread to regional lymph nodes; advanced BC [ABC]) and 6% were diagnosed with distant BC stage (i.e., cancer has metastasized; metastatic BC [MBC]) (3). The most common BC subtype is hormone receptor-positive/human epidermal growth factor receptor 2-negative (HR+/HER2−), with an age-adjusted rate of 90.0 new cases per 100,000 women. Among those with HR+/HER2− advanced/metastatic breast cancer (A/MBC), the 5-year relative survival rate between 2014–2020 was 86.7% and 31.9% in patients with ABC and MBC, respectively (3).

Therapeutic options for HR+/HER2− A/MBC have expanded with the introduction of cyclin-dependent kinase 4/6 inhibitors (CDK4/6i) into clinical practice. Palbociclib (Ibrance^®^, approved in 2015 in the United States [US]) (4), ribociclib (Kisqali^®^, approved in 2017 in the US) (5), and abemaciclib (Verzenio™, approved in 2017 in the US) (6) have been approved for use in combination with endocrine therapy (ET), including aromatase inhibitors (AIs) or fulvestrant, or as a single agent (abemaciclib). These approvals are based on the results of several randomized controlled trials (RCTs) (4–6) that met their study endpoint by demonstrating improvement in progression-free survival (PFS) among patients receiving CDK4/6i compared with those receiving ET monotherapy. Since their introduction, CDK4/6i plus ET have become the standard of care for first-line treatment of HR+/HER2− A/MBC, due to their efficacy, safety, and maintenance of quality of life when used as first-line treatment for patients with A/MBC (7–10).

Due to narrow patient eligibility criteria and study endpoints, RCTs are limited in providing a comprehensive understanding of clinical reality in routine practice. Real-world evidence (RWE) not only offers valuable insight into the effectiveness of treatments for HR+/HER2− A/MBC patient subgroups that may be underrepresented in clinical trials (e.g., older adults, those with comorbid or multimorbid conditions, Black, Indigenous, and People of Color [BIPOC] patients) but also reveals emerging patterns of care over extended periods, particularly after market approval. A systematic literature review (SLR) of RWE studies of CDK4/6i in the treatment of HR+/HER2− A/MBC was previously published including publications up to July 6, 2019 (11). At that time there were still limited follow-up data available, and limited real-world data for ribociclib and abemaciclib relative to palbociclib, given it was the first CDK4/6i approved for use in A/MBC. RWE for this class has grown in the years following, and some of the recent data have focused on describing outcomes among the three agents within the CDK4/6i class. The objective of this study was to understand the evolution of evidence around CDK4/6i to help inform clinical decision-making by highlighting the patient experience in the real world. Therefore, an updated SLR was conducted to summarize the effectiveness results of CDK4/6i from first-line RWE studies published since the previous review (11).

## 2 Materials and methods

### 2.1 Literature search

This SLR followed the Preferred Reporting for Systematic Reviews and Meta-Analyses (PRISMA) guidelines (12) (**Appendix A** in the **Supplementary Material**), which have been previously described (11). The search for the previous SLR (11) was performed on July 6, 2019. For this updated review, literature searches using OVID Medline, EMBASE, Cochrane Central Register of Controlled Trials and Cochrane Database of Systematic Reviews were conducted on October 7, 2020, June 1, 2021, December 1, 2022, January 6, 2023, October 18, 2023, and January 9, 2024 to capture all data published since the previous SLR search in 2019; results from these searches were pooled for this analysis. Details of the most recent search strategy are presented in **Appendix B** in the **Supplementary Material**. The data presented were collected uniformly across all searches. This review was not registered as it was developed *a priori*.

Updated grey literature searches of prespecified key clinical conferences were also performed to identify abstracts and posters from January 2020 to January 2024. These included the San Antonio Breast Cancer Symposium (SABCS), the American Society of Clinical Oncology (ASCO), the European Society for Medical Oncology (ESMO), ESMO BC, ESMO Asia, the Professional Society for Health Economics and Outcomes Research (ISPOR), and ISPOR Europe (EU). Only abstracts from January 2022 to January 2024 were included in the analysis to present only the most up-to-date information available in the literature.

### 2.2 Study selection and data extraction

Studies were assessed for eligibility by two independent reviewers using the systematic review software DistillerSR (DistillerSR Inc., Ottawa, Ontario, Canada) according to the predefined Population, Intervention, Comparison, Outcomes, and Study (PICOS) criteria (**Table 1**). Discrepancies between the two independent reviewers during screening were resolved by consensus, with any disputes resolved by a third reviewer.

**Table 1:** Population intervention comparators outcomes study design criteria.

Criteria	Inclusion criteria	Exclusion criteria
Population	• Patients aged ≥18 years old with HR+/HER2− A/MBC	• Only patients aged <18 years old • All other diseases
Intervention/comparators	• Palbociclib, ribociclib, and abemaciclib (within FDA indications) • Therapies used to treat locally advanced or metastatic, HR+, HER2− breast cancer via any route will be included as comparators	• Studies that do not include CDK 4/6is
Outcomes	• Effectiveness outcomes (e.g., clinical benefit rate, objective response rate, overall survival, progression-free survival) • Safety outcomes (e.g., overall rate of AEs, AEs of grade 3/4 severity, discontinuations due to AEs, grade 3/4 neutropenia) • Patient-reported outcomes/utility • Economic outcomes (e.g., direct and indirect costs, health resource utilization, wastage, productivity, and absenteeism) • Treatment duration, modifications, and discontinuations	• Studies that do not report any relevant outcomes
Study design*	• RWE studies (e.g., prospective and retrospective observational studies) • Published articles (July 6, 2019 to January 9, 2024) • Conference abstracts (January 1, 2022 to January 9, 2024**)	• Any non-RWE studies • Articles published before 2019 • Conference abstracts published before 2022
Language	• Articles in English^†^	• All non-English articles

*Case reports, commentaries, letters, consensus reports, nonsystematic reviews, systematic reviews, and meta-analyses (on relevant RWEs): the full texts of any relevant studies of these study designs that fit the criteria were acquired and hand-searched to find any additional relevant RWE studies not identified through the database searches.

**Relevant conference abstracts were included from SABCS, ASCO, ESMO, ESMO BC, ESMO Asia, and ISPOR.

^†^Citation retrieval was not limited by language. Records were categorized based on language during the title and abstract screening stage, and non-English abstracts were excluded. English abstracts with non-English articles were excluded at the full-text screening stage.

AEs, adverse events; A/MBC, advanced/metastatic breast cancer; CDK, cyclin-dependent kinase; HER2, human epidermal growth factor receptor 2; HR, hormone receptor; FDA, Food and Drug Administration; RWE, real-world evidence.

Studies were included if they reported RWE on patients aged ≥18 years with HR+/HER2− A/MBC receiving treatment with a CDK4/6i. Studies were excluded if published in any language other than English or before 2019. Only studies reporting data on CDK4/6i treatment in the first-line setting were included to focus on the treatment landscape wherein CDK4/6i are standard first-line treatment for HR+/HER2− A/MBC. To enhance the robustness of the review findings and relevance of the literature being summarized, studies with sample sizes of fewer than 100 patients and/or those that did not specify the line of therapy or specific CDK4/6i were excluded. Outcomes of interest included median PFS and/or median overall survival (OS) with corresponding hazard ratios (HRs), where available.

Data from the publications identified in this review were extracted into a standardized form in Microsoft^®^ Excel (Microsoft Corporation, Redmond, WA, US). A single reviewer performed data extraction and was independently assessed for accuracy and completeness by a second reviewer.

### 2.3 Data analysis

During data analysis, the included studies were categorized by study design (i.e., single-arm or comparative). Comparative studies, which assessed multiple treatment arms, were further stratified based on the comparator, distinguishing between ET and other CDK4/6i. Within each study design category, PFS and/or OS were then evaluated according to the type of CDK4/6i assessed (i.e., palbociclib, ribociclib, abemaciclib, and any CDK4/6i regimen), as well as the patient population (i.e., the overall population or specific subgroups). Any CDK4/6i regimen was defined as one that evaluated a CDK4/6i—whether palbociclib, ribociclib, or abemaciclib—but the results were not specific to any single CDK4/6i. Prespecified subgroups of interest, identified *a priori* in consultation with clinicians and listed in **Appendix C** in the **Supplementary Material**, were also included in the analysis. Of note is that the number of studies reporting on specific subgroups may not reflect the total number of included studies as some studies report on multiple subgroups.

The results were organized first to provide an overview of findings from single-arm studies, followed by a detailed analysis of comparative studies. Each section explored outcomes in the overall population and prespecified subgroups, offering a thorough understanding of PFS and/or OS across various CDK4/6i regimens.

### 2.4 Quality assessment

Of the included studies, only full-text publications were assessed for quality because conference abstracts often lack sufficient methodological data to assess study quality. Two independent reviewers performed the study quality assessments, resolving discrepancies through consensus. Risk of bias assessment was performed for included studies using the Newcastle-Ottawa scale (NOS) for nonrandomized studies (scores 7–9, 4–6, and <4 are considered low, intermediate, and high risk, respectively) (13). The ISPOR questionnaire (14) and ESMO Guidance for Reporting Oncology real-World Evidence (ESMO-GROW) checklist (15) were also used to determine the risk of bias for the included comparative studies and to assess appropriate reporting and transparency.

## 3 Results

### 3.1 Literature search and study selection

A total of 6737 records were identified, of which 4845 were found through database searches and 1892 through grey literature searches. The results of the literature search and study selection processes of each update are shown in **Appendix D** in the **Supplementary Material**. After the removal of duplicates, 4836 records were screened at the title and abstract stage, of which 2491 full texts were retrieved and assessed for eligibility. In total, 882 records were included in the SLR. The reasons for exclusion at the full-text stage of each update are summarized in **Appendix D** in the **Supplementary Material**.

Among the 882 records included in the SLR, 787 were excluded for reasons such as small sample sizes (<100 patients), unspecified line of therapy or type of CDK4/6i assessed, data on CDK4/6i treatment beyond first-line therapy, and/or lack of reported outcomes of interest (i.e., PFS and/or OS). Consequently, 95 publications (51 full-text articles and 44 conference abstracts/posters) representing 82 unique studies reported effectiveness data in the first-line setting and were included in the qualitative synthesis. Among these, 35 studies (42.7%) focused on palbociclib, 6 (7.3%) featured ribociclib, and 3 (3.7%) assessed abemaciclib. The remaining 38 studies (46.3%) investigated more than one CDK4/6i. A list of included studies is shown in **Appendix E** in the **Supplementary Material**.

**Figure 1:**
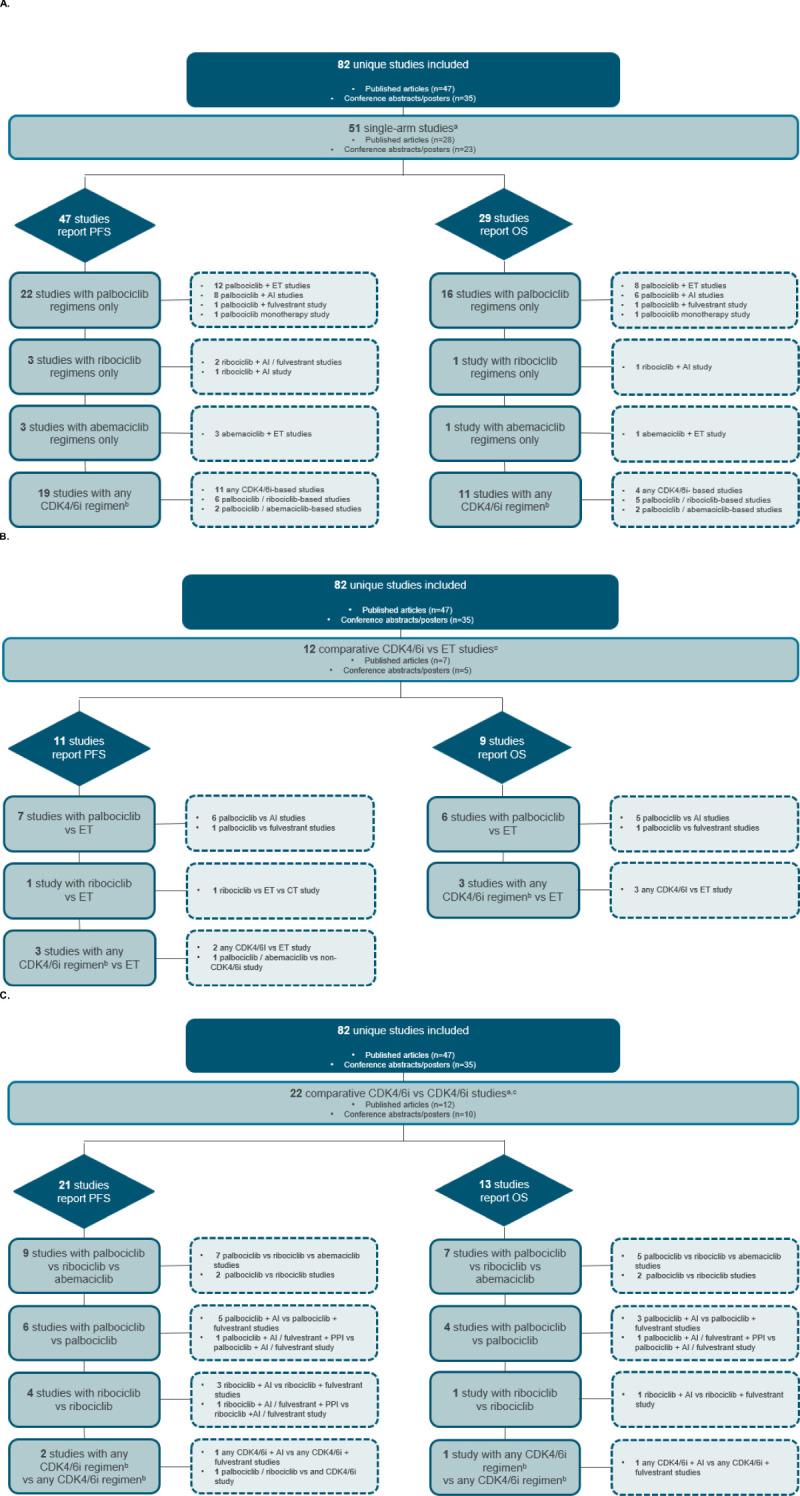
Study attrition diagram for **(A)** single-arm studies **(B)** comparative CDK4/6i versus ET studies, and **(C)** comparative CDK4/6i versus CDK4/6i studies. ^a^The GOIRC-04-2019 and REACHAUT studies had multiple associated records using single-arm and comparative analyses and are thus counted in both study design categories. ^b^Any CDK4/6i regimen was defined as that in which a CDK4/6i—whether palbociclib, ribociclib, or abemaciclib—was evaluated, but the results were not specific to any single CDK4/6i. ^c^The RIBANNA study had multiple associated records, including ET and CDK4/6i comparator arms, resulting in its inclusion in both comparative study design categories. AI, aromatase inhibitor; CDK4/6i, cyclin-dependent kinase 4/6 inhibitors; CT, chemotherapy; ET, endocrine therapy; OS, overall survival; PFS, progression-free survival; PPII, proton pump inhibitor.

The majority (n=51 [62.2%]) of the unique studies were single-arm (**Figure 1A**). The remaining studies were comparative in design (direct comparison or descriptive), with 12 studies comparing CDK4/6i to ET (**Figure 1B**) and 22 describing or comparing effectiveness studies evaluating multiple CDK4/6i (**Figure 1C**). Notably, three unique studies—GOIRC-04-2019, REACHAUT, and RIBANNA—were each represented by multiple associated records that led to their inclusion in both the single-arm and comparative design categories. The GOIRC-04-2019 study had two records: one for a single-arm analysis (16) and another for a comparison of multiple CDK4/6i (17). Similarly, the REACHAUT study included a single-arm analysis (18) and a comparison of two ribociclib regimens with different backbone therapies (AI or fulvestrant) (19). The RIBANNA study was represented by three abstracts, two of which compared CDK4/6i with ET (20, 21), whereas the third was a comparison of ribociclib treatment in combination with different ET therapies (AI or fulvestrant) (22).

**Figure 2:**
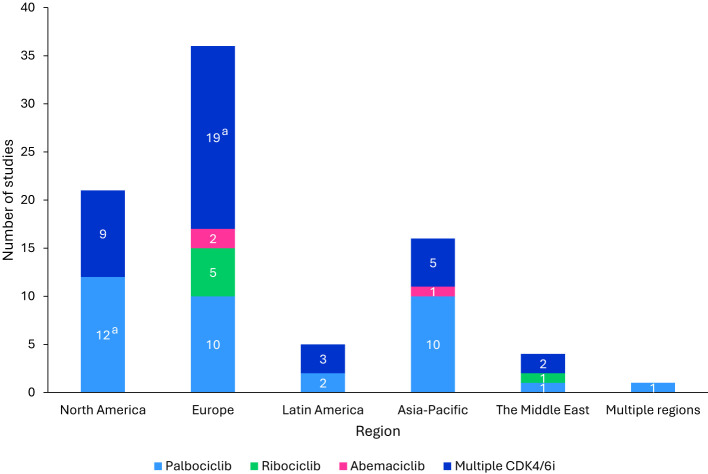
Regional distribution of included studies. ^a^The GOIRC-04-2019 study had multiple associated records, one evaluating palbociclib in North America and the other evaluating any CDK4/6i regimen in Europe. As such, this study is accounted for in both categories. ^b^Studies were classified as “Multiple CDK4/6i” if two or more specified CDK4/6i were included in the study. CDK4/6i, cyclin-dependent kinase 4/6 inhibitors.

Across all study types, the majority (43.9%) were conducted in Europe, with the highest representations from the United Kingdom (n=7), Spain (n=7), and Italy (n=6). Other regions included North America, Latin America, Asia-Pacific, and the Middle East (**Figure 2**).

### 3.2 Quality assessment

For all included full-text reports, the NOS quality scores ranged from 4–8 points out of 9; 66.7% (34/51) had a score of 4–6, and 33.3% (17/51) had a score of 7 or 8. Comparative studies were assessed using the ISPOR questionnaire and ESMO-GROW checklist, which indicated that the overall credibility of these reports was generally sufficient (87.0% [20/23] reports were identified as being of sufficient credibility). However, it is important to note that studies with an overall rating of sufficient credibility may still have significant limitations.

Among the assessed studies, the main source of biases identified concerned the study design and analyses. Nine studies used stabilized inverse probability of treatment weighting (sIPTW) and 1:1 propensity score matching (PSM) methods to control for differences in baseline demographics and clinical characteristics between treatment groups (23–31). However, the majority of studies did not provide evidence that robust statistical methods, such as sIPTW or PSM, were used to ensure comparability of treatment groups and only reported results descriptively. Moreover, the nonrandomized nature of these studies means confounding factors could affect these results. Data collection methods, including data cleaning and validation processes, were generally consistent among the assessed studies. All records underwent chart abstraction by certified tumor registrars. For two studies, this was followed by a quality control review for transparency and completeness by clinical analytics teams in two studies (28, 32). The remaining studies did not provide adequate details on how the different data sources were assessed.

Full results for the quality assessments are presented in **Appendix F** in the **Supplementary Material**.

### 3.3 Effectiveness of CDK4/6i in single-arm studies

Of the 51 single-arm studies, PFS data were reported in 47, whereas OS data were reported in 29 (**Figure 1A**). Data from studies evaluating any CDK4/6i regimen without CDK4/6i-specific results are summarized in **Appendix G** in the **Supplementary Material**.

#### 3.3.1 Progression-free survival

##### 3.3.1.1 Palbociclib

The PFS data for patients receiving palbociclib were reported in 22 single-arm studies (**Figure 1A**). Of these, 11 studies reported results for the overall population (**Supplementary Table 1**, **Appendix H** in the **Supplementary Material**), three reported results for specific subgroups of patients (**Supplementary Tables 1**, **2**, **Appendix I** in the **Supplementary Material**), and the remaining eight reported both overall and subgroup population data.

###### 3.3.1.1.1 Overall population

In 10 single-arm studies evaluating palbociclib plus ET (unspecified) in the overall patient population, median PFS ranged from 12.1 (n=103) (33) to 37.8 months (n=434) (34). The PFS results were similar to those of palbociclib plus AI, reported in seven single-arm studies, with median PFS ranging from 11.8 (35) to 39.0 months (16) in the overall population. However, it should be noted that the study with a median PFS of 11.8 months had only 30 patients in the arm receiving palbociclib plus AI (letrozole) (35). Omitting this study resulted in a median PFS range of 28.7 (n=305) (36) to 39.0 months (n=241) (16). In comparison, median PFS was lower in one single-arm study evaluating palbociclib plus fulvestrant in the overall population, with a median PFS of 19.6 months (n=317) (37). Data for palbociclib monotherapy was reported in a conference abstract from Taiwan (n=53), indicating that median PFS was not reached after a median follow-up of 24.5 months (38) (**Supplementary Table 1**, **Appendix H** in the **Supplementary Material**). Of the nine studies with quality assessment, all were considered intermediate risk (NOS score of 4–6) (32–36, 39–43).

###### 3.3.1.1.2 Subgroups

Subgroups based on the types of metastases (e.g., visceral, bone, liver, and so on) were assessed in six single-arm studies. Among patients presenting with visceral metastases, median PFS ranged from 15.3 (n=65) (42) to 27.9 months (n=78) (32), whereas those with no visceral metastases had a higher median PFS, ranging from 27.8 (n=212) (44) to 31.3 months (n=240) (42). For patients with bone-only metastases, the median PFS ranged from 20.0 (n=30) (45) to 44.9 months (n=123) (32). One study found that patients without liver metastases (n=245) had a statistically significant improvement in median PFS (12.7 months) compared with those with liver metastasis (n = 60; 31.3 months) with a HR of 2.17 (1.42-3.31; *P* < 0.001) (42).

Subgroups based on hormonal status (e.g., progesterone receptor [PR]-positive [+]/PR-negative [−], HER2 status, estrogen receptor [ER]/PR strong/weak) were assessed in four studies. Among patients with PR+ disease, the median PFS ranged from 24.5 (n=74) (42) to 38.0 months (n=127) (41), whereas those with PR− disease had a lower median PFS, ranging from 17.9 (n=75) (42) to 18.0 months (n=23) (41). Where reported, this trend was statistically significantly in favor of those with PR+ disease. Additionally, median PFS was generally similar among patients with HER2-zero (13.0 [n=83] (41) to 23 months (40)) and HER2-low status (19.0 [n=71] (40) to 25.0 months [n=67] (41)). One study also compared patients with strong (n=425) versus weak ER/PR expression (n=92) and found that PFS was statistically significantly higher in those with strong expression (42).

Subgroups based on ET response (e.g., *de novo*, recurrent, endocrine-resistant/sensitive) were assessed in three studies. Median PFS ranged from 14 (46) to 14.5 months (n=193) (44) for patients who relapsed within 12 months, 27.3 (n=86) (44) to 29.0 months (n=220) (46) for those who relapsed after more than 12 months, and 28.0 (n=233) (46) to 33.6 months (n=109) (44) for *de novo* patients. One study observed the highest median PFS in patients with no ET (25.4 months; n=126), followed by those with secondary resistance (20.3 months; n=58) and primary resistance (12.7 months; n=38) (42). A statistically significant difference was noted, with patients showing primary ET resistance having a higher risk of progression compared to those with no ET (HR: 1.91, 95% CI: 1.13–3.24; P=0.022). No significant difference was observed for secondary ET resistance (HR: 0.87, 95% CI: 0.52–1.49; P=0.022) or ET-sensitive patients (HR: 0.81, 95% CI: 0.50–1.32; P=0.022) compared to those with no ET (42).

Subgroups based on dose modifications were assessed in three studies. Median PFS was similar for patients with and without dose reductions, ranging from 25.0 (n=80) (41) to 28.0 months (n=377) (47) and 19.0 (n=385) (47) to 22.0 months (n=70) (41), respectively, with no statistically significant difference reported. One study reported dose modifications specifically due to grade 3 afebrile neutropenia and showed a statistically significant lower 24-month PFS rate among patients who required dose modifications (55.3%; n=128) than those who maintained their doses (67.9%; n=174) (34).

Three studies assessed subgroups based on risk factors (e.g., comorbidities, Charleson Comorbidity Index [CCI] score). Median PFS ranged from 12.5 to 23.7 months among patients with various comorbid disorders (e.g., vascular, psychiatric, metabolic, lymphatic, cardiac; n range: 32-495) (48), with increasing presence of risk factors generally correlating with lower median PFS (32, 42); however, no tests for statistical significance were conducted.

Additional prespecified subgroups of interest were assessed in the single-arm studies that reported PFS data for palbociclib-based regimens, which included menopausal status, Eastern Cooperative Oncology Group (ECOG) score, age, race/ethnicity, and palbociclib starting dose. The results of these studies are described in **Supplementary Table 1**, **Appendix I** in the **Supplementary Material**. The PFS results for studies that assessed other subgroups are detailed in **Supplementary Table 2**, **Appendix I** in the **Supplementary Material**.

Overall, in studies with subgroups of interest data, six received a quality assessment, with NOS scores of 5 or 6 (intermediate risk of bias) (32, 34, 40–42, 49).

##### 3.3.1.2 Ribociclib

The PFS data for patients receiving ribociclib were reported in three single-arm studies (**Figure 1A**). Of these, one study reported results for the overall population (**Supplementary Table 2**, **Appendix H** in the **Supplementary Material**) (50), another study reported subgroup-only results (**Supplementary Table 3**, **Appendix I** in the **Supplementary Material**) (51), and the third reported both overall and subgroup population data (18).

###### 3.3.1.2.1 Overall population

In a conference abstract of the single-arm REACHAUT study, the median PFS was 29.7 months among patients receiving ribociclib plus AI or fulvestrant in the overall patient population (n=283), with a median follow-up duration of 14.4 months (18). In another abstract of a single-arm study evaluating ribociclib plus AI (n=154), median PFS was reported to be 20.6 months, although the duration of follow-up was not provided (**Supplementary Table 2**, **Appendix H** in the **Supplementary Material**) (50).

###### 3.3.1.2.2 Subgroups

Two single-arm studies assessing ribociclib in combination with either AI or fulvestrant provided insights into prespecified subgroups of interest. In addition to the overall population, the conference proceeding for the REACHAUT study also evaluated patients with visceral metastases (n=116) and reported a median PFS of 32.7 months (18). The other study compared outcomes between patients who did not require a dose reduction and those who experienced a late dose reduction (51). After a median follow-up time of 18.4 months, the median PFS for patients without a dose reduction (n=46) was 15.6 months, while the median PFS for those with a late dose reduction (n=31) was not reached (51) (**Supplementary Table 3**, **Appendix I** in the **Supplementary Material**). This study was assessed for quality with a NOS score of 5 and judged to be of sufficient credibility (51).

##### 3.3.1.3 Abemaciclib

The PFS data for patients receiving abemaciclib in combination with ET (unspecified) in the overall population were reported in three single-arm studies (**Figure 1A**). Median PFS across these studies ranged from 21.4 (n=63) to 23.0 months (n=69), with 71.6% to 81.1% of patients achieving PFS at 12 months (**Supplementary Table 3**, **Appendix H** in the **Supplementary Material**) (52–54). Notably, none of the studies reported PFS data for specific patient subgroups. Only one of these studies was evaluated for quality (54); it received an NOS score of 4 and was judged to have insufficient credibility.

#### 3.3.2 Overall survival

##### 3.3.2.1 Palbociclib

The OS data for patients receiving palbociclib were reported in 16 single-arm studies (**Figure 1A**). Of these, six studies reported results for the overall population (**Supplementary Table 1**, **Appendix H** in the **Supplementary Material**), two reported results exclusively for specific subgroups of patients (**Supplementary Tables 1**, **2**, **Appendix I** in the **Supplementary Material**), and the remaining eight studies reported both overall and subgroup population data.

###### 3.3.2.1.1 Overall Population

In seven single-arm studies evaluating palbociclib plus ET (unspecified) in the overall patient population, median OS ranged from 33.0 (n=1066) (41) to 42 months (n=762) (46) when reached, with one study from Chile reporting a median OS of 111 months (n=67) (55). One of these studies (n=434) also reported a 24-month OS rate of 91.4% (34). Across five single-arm studies evaluating palbociclib plus AI, median OS was not reached where reported; however, the 24-month OS rate ranged from 70.0% (35) to 78.0% (n=242) (32). In comparison, median OS was 44.1 months in one single-arm study evaluating palbociclib plus fulvestrant (n=317) (37) (**Supplementary Table 1**, **Appendix H** in the **Supplementary Material**). Data for palbociclib monotherapy was reported in a conference abstract from Taiwan (n=53), indicating that median OS was not reached after a median follow-up of 24.5 months (**Supplementary Table 1**, **Appendix H** in the **Supplementary Material**) (38). The six studies reporting median OS underwent quality assessment and had NOS scores from 4 to 6 (35, 40–43, 55).

###### 3.3.2.1.2 Subgroups

Four single-arm studies included the same subgroups based on hormonal status as described in Section 3.3.1.1.2. Where median OS was reached, it ranged from 39.0 (n=127) (41) to 44.0 months (n=530) (56) for patients with PR+ disease and 28.0 (n=23) (41) to 40.0 months (n=213) (56) for those with PR− disease, with a statistically significant difference favoring PR+ patients. In two studies comparing patients with HER2-zero and HER2-low status, one found a higher OS rate in those with HER2-zero status, while the other reported a longer median OS in those with HER2-low status; however, these results were not statistically significant (**Supplementary Table 1**, **Appendix I** in the **Supplementary Material** (40).

Additional prespecified subgroups of interest were assessed in the single-arm studies that reported OS data for palbociclib-based regimens, which included metastases (e.g., bone, visceral), comorbidities (e.g., disorders, CCI score), dose modification, ECOG score, and ET response (e.g., *de novo*, recurrent). The results of these studies are described in **Supplementary Table 1**, **Appendix I** in the **Supplementary Material**. The OS results for studies that assessed other subgroups are detailed in **Supplementary Table 2**, **Appendix I** in the **Supplementary Material**.

Overall, in studies with subgroups of interest data, six of these studies were evaluated for quality (32, 34, 40–42, 49); all were identified as having an intermediate risk of bias (NOS scores of 5 or 6).

##### 3.3.2.2 Ribociclib

The OS data for patients in the overall population receiving ribociclib in combination with ET (unspecified) were reported in one single-arm study (**Figure 1A**). In a conference abstract of the single-arm REACHAUT study (n = 283), the 12-month OS rate was 90.3% after a median follow-up duration of 14.4 months (**Supplementary Table 2**, **Appendix H** in the **Supplementary Material**) (18). No OS data for specific patient subgroups were reported.

##### 3.3.2.3 Abemaciclib

The OS data for patients in the overall population receiving abemaciclib in combination with ET (unspecified) were reported in one single-arm study (n = 69; **Figure 1A**). In a conference abstract that reported effectiveness results from the Slovenian National Institute of Public Health and the Slovenian Cancer Registry, median OS was not reached after a median follow-up of 24 months (**Supplementary Table 3**, **Appendix H** in the **Supplementary Material**) (53). No OS data for specific patient subgroups were reported.

### 3.4 Effectiveness of CDK4/6i versus ET

Of the 12 comparative (including direct and descriptive comparison) studies evaluating CDK4/6i versus ET, PFS data were reported in 11 studies, whereas OS data were reported in nine studies (**Figure 1B**). Of note, although there are comparative studies for ribociclib versus ET, only PFS data were reported. Additionally, no studies evaluated abemaciclib in comparison to ET. Results from studies evaluating any CDK4/6i regimen versus ET without CDK4/6i-specific data are summarized and included in **Appendix J** in the **Supplementary Material**.

#### 3.4.1 Progression-free survival

##### 3.4.1.1 Palbociclib

**Figure 3:**
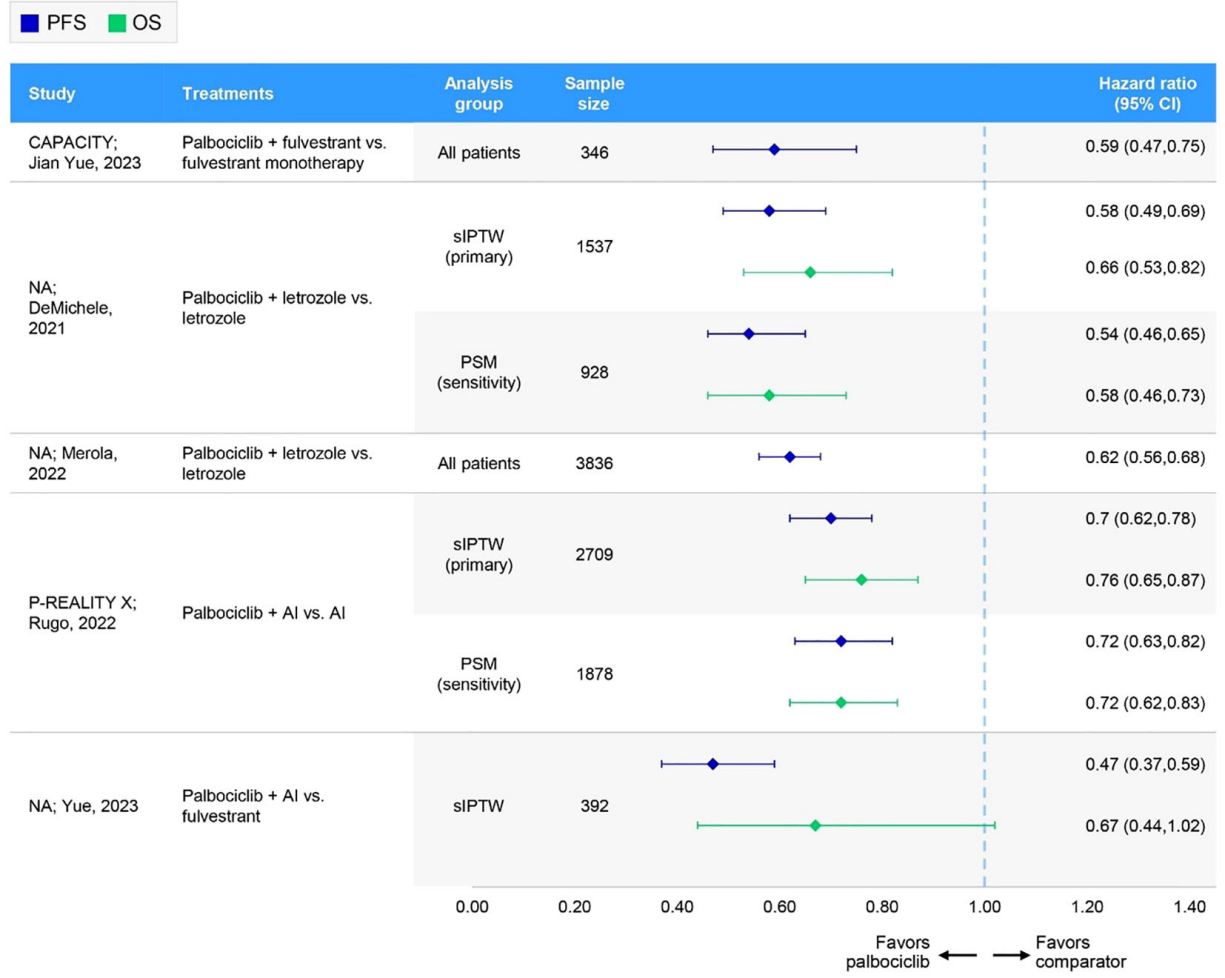
Forest plot of hazard ratios for effectiveness outcomes for overall first-line palbociclib in comparative RWE studies versus ET. AI, aromatase inhibitor; CI, confidence interval; NA, not applicable; OS, overall survival; PFS, progression-free survival; PSM, propensity score matching; sIPTW, stabilized inverse probability of treatment weighting.

The PFS data for patients receiving palbociclib versus ET were reported in seven studies (**Figure 1B**). Of these, three studies reported results for the overall population (**Table 2**, **Figure 3**), two focused on specific patient subgroup populations (**Supplementary Table 4**, **Appendix I** in the **Supplementary Material**), and the remaining two reported data for both the overall and subgroup populations.

**Table 2:** Effectiveness outcomes for overall first-line palbociclib in comparative RWE studies versus ET.

Study name; reference	Treatment	Subgroup	Sample size	PFS			OS		
				Median (95% CI), months	HR (95% CI); *P* value)	At latest time point, n (%)	Median (95% CI), months	HR (95% CI); *P* value	At latest time point, n (%)
CAPACITY; ASCO23-003-Jian Yue-2023	Palbociclib + fulvestrant	All patients	193	20 (17.3–22.7)	0.59 (0.47-0.75); *P*<0.0001	12 months: NR (65.8)	Not reached	NR	NR
	Fulvestrant monotherapy	All patients	153	12 (9.6–14.3)		12 months: NR (46.4)	65 (55.6–75.4)		NR
DeMichele 2021; 1265-DeMichele-2021	Palbociclib + letrozole	sIPTW cohort	839	20 (17.5–21.9)	sIPTW: 0.58 (0.49-0.69); *P* < 0.0001 PSM: 0.54 (0.46-0.65); *P*<0.0001	NR	Not reached	sIPTW: 0.66 (0.53-0.82); *P*, 0.0002). PSM: 0.58 (0.46-0.73); *P* < 0.0001	NR
		PSM cohort	464	20.2 (18.2–23.7)		NR	Not reached		NR
	Letrozole	sIPTW cohort	698	11.9 (10.5–13.7)		NR	43.1 (34.3–not reached)		NR
		PSM cohort	464	11.9 (10.4–14.5)		NR	43.1 (34.3–not reached)		NR
P-REALITY X; 176-Rugo-2022	Palbociclib + AI	sIPTW cohort	1,572	19.3 (17.5–20.7)	sIPTW: 0.70 (0.62-0.78); *P* < 0.0001 PSM: 0.72 (0.63-0.82); *P* < 0.0001	NR	49.1 (45.2–57.7)	sIPTW: 0.76 (0.65-0.87); *P* < 0.0001 PSM: 0.72 (0.62-0.83); *P* < 0.0001	48 months: 722 (54.5)
		PSM cohort	939	19.8 (17.3–21.9)		NR	57.8 (47.2–not reached)		48 months: 532 (46.8)
	AI	sIPTW cohort	1,137	13.9 (12.5–15.2)		NR	43.2 (37.6–48)		48 months: 707 (45.2)
		PSM cohort	939	14.9 (12.9–16.9)		NR	43.5 (37.6–48.9)		48 months: 441 (47)
284-Merola-2022	Palbociclib + letrozole	All patients	1,299	23.1 (20.8–24.7)	0.62 (0.56–0.68); NR	NR	NR	NR	NR
	Letrozole	All patients	2,537	14.2 (12.8–15.9)		NR	NR		NR
SABCS23-059-Yue-2023	Palbociclib + AI	sIPTW	240	22 (NR)	0.47 (0.37-0.59); *P*<0.0001	NR	Not reached	0.67 (0.44–1.02); *P*<0.94	NR
	Fulvestrant	sIPTW	152	14 (NR)		NR	57 (54–66)		NR

AI, aromatase inhibitor; CDK4/6i, cyclin-dependent kinase 4/6 inhibitors; CI, confidence interval; NR, not reported; OS, overall survival; PFS, progression-free survival; PSM, propensity score matching; RWE, real-world evidence.

###### 3.4.1.1.1 Overall population

In four comparative studies that used sIPTW as the primary analysis of the outcome measure, palbociclib plus AI consistently demonstrated greater PFS benefits relative to control treatment with AI alone, resulting in a median PFS of 19.3 (n=1572) (28) to 23.1 months (n=1229) (31) for palbociclib plus AI and 11.9 (n=698) (27) to 13.9 months (n=1137) (28) for the control. Similarly, the CAPACITY study conference abstract reported improved PFS outcomes with palbociclib plus fulvestrant (n=193) compared with the control arm of fulvestrant alone (n=153), demonstrating a longer median PFS (20.0 months vs. 12.0 months) and a greater proportion of patients experiencing PFS at 12 months (65.8% vs. 46.4%) (57). Overall, palbociclib-based regimens consistently demonstrated statistically significant improvements in PFS compared to ET (**Table 2**, **Figure 3**). Of the three studies assessed for quality (27, 28, 31), two had a low risk of bias (NOS scores of 8) (27, 28), while one had an intermediate risk (NOS score of 5) (31). ISPOR questionnaire assessment found all three to be of sufficient overall credibility.

###### 3.4.1.1.2 Subgroups

Three separate records of the P-REALITY X study assessed three different subgroups using data from the Flatiron Health database: patients aged 75 years or older (23), those with lung and/or liver metastases (24), and those with cardiovascular disease (58). Another unique study also included subgroups based on lung or liver metastases (24), while a third study assessed patients aged 65 years or older (30). The fourth study assessed African American subgroups (29). All four unique comparative studies demonstrated statistically significant improvements in PFS when treated with palbociclib plus AI versus AI alone (**Supplementary Table 4**, **Appendix I** in the **Supplementary Material**). The four publications assessed for quality had NOS scores of 8 and sufficient credibility according to the ISPOR questionnaire (23, 24, 29, 30).

##### 3.4.1.2 Ribociclib

The PFS data for patients receiving ribociclib plus AI or fulvestrant versus ET and chemotherapy alone were reported in one comparative study (RIBANNA) (**Figure 1B**).

###### 3.4.1.2.1 Overall population

In a conference abstract for the fifth interim analysis of the RIBANNA study, patients receiving ribociclib in combination with AI or fulvestrant (n=2163) had a median PFS of 32.2 months, similar to the 35.2 months observed with ET monotherapy (n=237). In contrast, patients receiving chemotherapy alone (n=181) had a shorter median PFS of 16.7 months (**Table 3**) (20).

**Table 3:** Effectiveness outcomes for overall first-line ribociclib in comparative RWE studies versus ET.

Study name; reference	Treatment	Subgroup	Sample size	PFS			OS		
				Median (95% CI), months	HR (95% CI); *P* value	At latest time point, n (%)	Median (95% CI), months	HR (95% CI); *P* value	At latest time point, n (%)
RIBANNA; SABCS22-090-Fasching-2022	Ribociclib + AI or fulvestrant	All patients	2,163	32.2 (29.3–34.8)	NR	NR	NR	NR	NR
	ET alone	All patients	237	35.2 (23.9–44.2)		NR	NR		NR
	CT alone	All patients	181	16.7 (9.9–17.5)		NR	NR		NR

AI, aromatase inhibitor; CDK4/6i, cyclin-dependent kinase 4/6 inhibitors; CI, confidence interval; CT, chemotherapy; ET, endocrine therapy; NR, not reported; OS, overall survival; PFS, progression-free survival; RWE, real-world evidence.

###### 3.4.1.2.2 Subgroups

In specific subgroup analyses of the RIBANNA study, patients with liver metastases receiving ribociclib plus AI or fulvestrant (n=384) had a median PFS of 16.6 months, which was significantly shorter than the 36.6 months observed in patients without liver metastases (n=1427). Conversely, those receiving ET alone had a median PFS of 10.4 months with liver metastases (n=23) versus 37.6 months without it (n=169). For patients on chemotherapy, the median PFS was 13.5 months in those with liver metastases (n=65) compared with 16.8 months in those without (n=78; **Supplementary Table 5**, **Appendix I** in the **Supplementary Material**) (21).

#### 3.4.2 Overall survival

##### 3.4.2.1 Palbociclib

The OS data for patients receiving palbociclib versus ET were reported in six studies (**Figure 1B**). Of these, two studies reported results for the overall population (**Table 2**, **Figure 3**), two studies focused on specific patient subgroup populations (**Supplementary Table 4**, **Appendix I** in the **Supplementary Material**), and the remaining two studies reported data for both the overall and subgroup populations.

###### 3.4.2.1.1 Overall population

In three comparative studies evaluating palbociclib plus AI versus AI alone, median OS was generally not reached for patients receiving palbociclib (27, 57), with the exception of one study. In P-REALITY X, the median OS was 49.1 and 57.8 months, with 48-month OS rates of 54.5% and 46.8% in two weighted patient groups (i.e., sIPTW [n=1572] and PSM [n=939]) (28). In contrast, the median OS for the control group (AI alone) ranged from 43.1 (n=698) (27) to 57.0 months (n=152) (57) across the studies, with 45.2% and 47.0% of patients experiencing OS at 48 months in the sIPTW and PSM cohorts in P-REALITY X (28). Similarly, the CAPACITY study conference abstract reported that the median OS was not reached with palbociclib plus fulvestrant, whereas the median OS was 65.0 months with fulvestrant alone (57). Overall, palbociclib-based regimens consistently demonstrated statistically significant improvements in OS compared to ET (**Table 2**, **Figure 3**). Of the two studies that underwent quality assessment, both were of high quality (NOS score of 8 and sufficient credibility) (27, 28).

###### 3.4.2.1.2 Subgroups

The same three comparative studies reporting PFS data for subgroups of patients described in Section 3.4.1.1.2 also reported OS data. Consistent with the PFS results, all studies demonstrated statistically significant improvements in OS with palbociclib plus AI compared with AI alone among older patients (23, 30), those with lung and/or liver metastases (26, 59), patients with cardiovascular disease (58), and African American patients (29) (**Supplementary Table 4**, **Appendix I** in the **Supplementary Material**). Assessed publications were all low risk and had sufficient credibility (23, 24, 29, 30).

### 3.5 Comparative effectiveness of CDK4/6i studies

Of the 22 comparative studies (including direct and descriptive comparison) evaluating a particular CDK4/6i regimen versus another CDK4/6i regimen, 12 studies directly compared two or more specified CDK4/6 inhibitors. The remaining studies evaluated the same CDK4/6i in combination with different backbone therapies (e.g., palbociclib plus AI vs. palbociclib plus fulvestrant). For completeness, these studies are summarized and included in **Appendix K** in the **Supplementary Material**.

#### 3.5.1 Progression-free survival

##### 3.5.1.1 Palbociclib versus ribociclib versus abemaciclib

**Figure 4:**
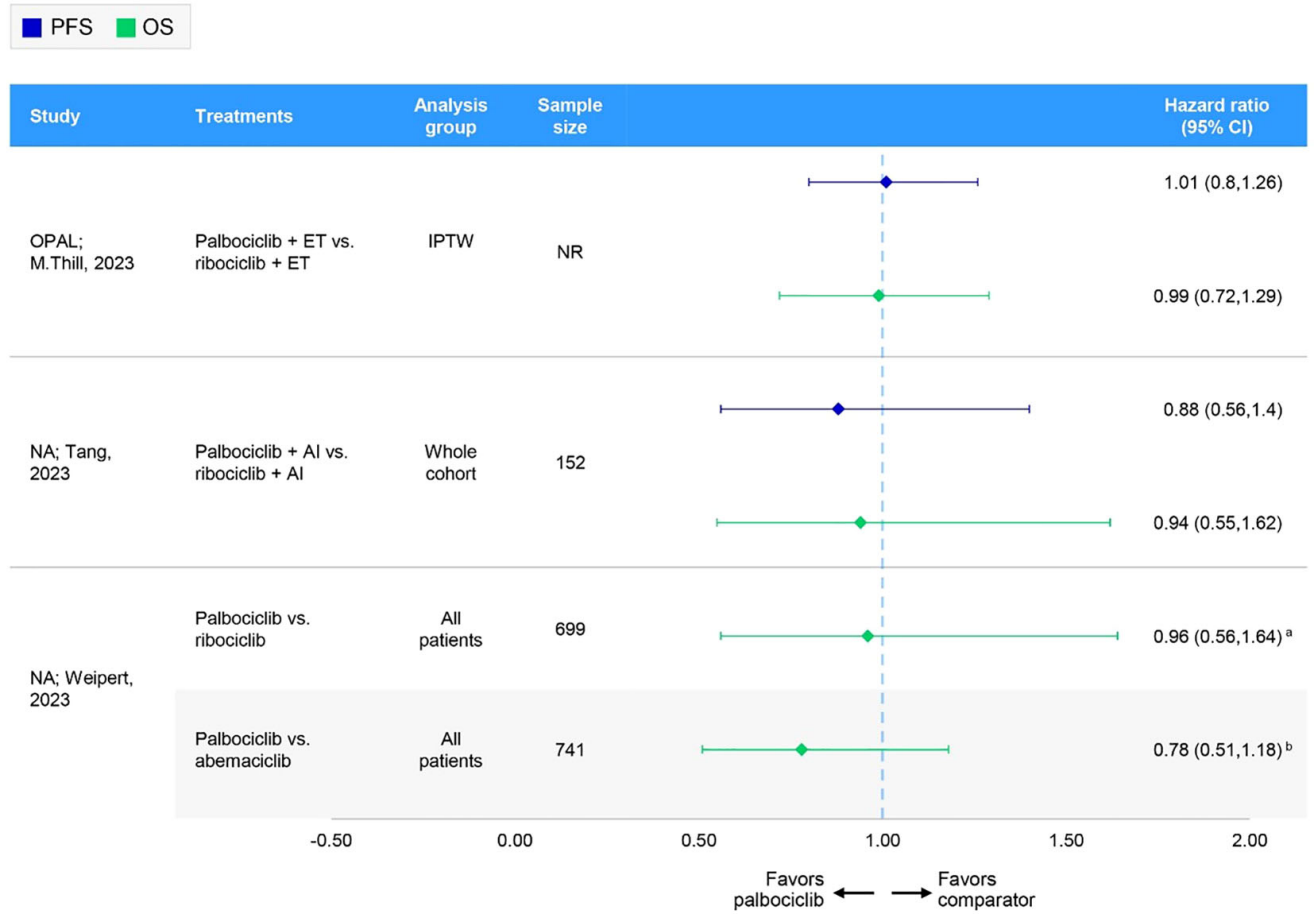
Forest plot of hazard ratios for effectiveness outcomes for overall first-line comparative RWE studies assessing two or more specified CDK4/6i. ^a^Hazard ratio has been inverted from that originally published by Weipert et al. for abemaciclib vs palbociclib: 1.29 (95% CI: 0.85-1.96). (60) ^b^Hazard ratio has been inverted from that originally published by Weipert et al. for ribociclib vs palbociclib: 1.04 (95% CI: 0.61-1.77) (60). AI, aromatase inhibitor; CI, confidence interval; ET, endocrine therapy; IPTW, inverse probability of treatment weighting; NA, not applicable; NR, not reported; OS, overall survival; PFS, progression-free survival.

The PFS data for patients receiving palbociclib, ribociclib, or abemaciclib were available in nine comparative studies (**Figure 1C**). Five studies reported results for the overall population (**Table 4**, **Figure 4**), while four reported overall and subgroup population data (**Supplementary Table 6**, **Appendix I** in the **Supplementary Material**).

**Table 4:** Effectiveness outcomes for overall first-line comparative RWE studies assessing two or more specified CDK4/6i.

Study name; reference	Treatment	Subgroup	Sample size	PFS			OS		
				Median (95% CI), months	HR (95% CI); *P* value	At latest time point, n (%)	Median (95% CI), months	HR (95% CI); *P* value	At latest time point, n (%)
GOIRC-04-2019; SABCS23-009-Moscetti-2023	CDK4/6i + AI/fulvestrant	All patients	134	31 (21–39)	NR	NR	NR	NR	NR
	Palbociclib + AI/fulvestrant	All patients	61	23.43 (15.4–31.5)		NR	NR		NR
	Ribociclib + AI/fulvestrant	All patients	44	39.9 (30.9–49)		NR	NR		NR
	Abemaciclib + AI/fulvestrant	All patients	25	23.3 (13.3–33.1)		NR	NR		NR
OPAL; SABCS23-007-M.Thill-2023	Palbociclib + ET	IPTW	NR	26.7 (23.2–30.7)	1.01 (0.80-1.26); NR	NR	41.4 (38.8–50.3)	0.99 (0.72-1.29); NR	NR
	Ribociclib + ET	IPTW	NR	27 (21.1–30.7)		NR	49.3 (36.9–not reached)		NR
3400-Tang-2023	Palbociclib + AI	Whole cohort	114	23.9 (NR)	0.88 (0.56-1.40); *P*=0.60	NR	49.5 (NR)	0.94 (0.55-1.62); *P*=0.94	NR
	Ribociclib + AI	Whole cohort	38	19.8 (NR)		NR	40.4 (NR)		NR
3527-Quieroz-2023	Abemaciclib + ET	All patients	NR	14 (NR)	NR	NR	NR	NR	NR
	Palbociclib + ET	All patients	NR	24 (8–40)		NR	NR		NR
	Ribociclib + ET	All patients	NR	30 (23.5–36.5)		NR	NR		NR
4112-Cejuela-2023	CDK4/6i + ET	All patients	206	35.61 (NR)	NR	NR	57.56 (44.6–70.5)	NR	NR
	Abemaciclib + ET	All patients	56	39.49 (NR)		NR	Not reached		NR
	Palbociclib + ET	All patients	96	30.03 (NR)		NR	Not reached		NR
	Ribociclib + ET	All patients	54	31.14 (NR)		NR	Not reached		NR
4132-Buller-2023	Palbociclib + ET	All patients	120	27.9 (23–32.5)	NR	NR	38 (33.5–42.5)	NR	NR
	Ribociclib + ET	All patients	28	31.1 (NR)		NR	NR		NR
	Abemaciclib + ET	All patients	44	17 (10.41–23.59)		NR	34.3 (NR)		NR
4506-Tang-2023	Palbociclib + ET	All patients	162	27.5 (NR)	NR	5 years: NR (20.88)	49.5 (NR)	NR	5 years: NR (48.54)
	Ribociclib + ET	All patients	46	25.7 (NR)		5 years: NR (32.58)	50.2 (NR)		5 years: NR (42.33)
	Abemaciclib + ET	All patients	19	NR		5 years: NR (66.8)	NR		5 years: NR
ESMO23-022-Gullick-2023	Palbociclib + ET	All patients	473	31 (25–35)	NR	NR	NR	NR	NR
	Abemaciclib + ET	All patients	33	16 (9-NR)		NR	NR		NR
	Ribociclib + ET	All patients	38	44 (21-NR)		NR	NR		NR
ESMO23-066-Lenza-2023	Palbociclib + ET	All patients	NR	16 (NR)	NR	NR	44 (NR)	NR	NR
	Ribociclib + ET	All patients	NR	14 (NR)		NR	52 (NR)		NR
	Abemaciclib + ET	All patients	NR	17 (NR)		NR	NR		NR
SABCS23-065- Weipert-2023	Palbociclib + ET	All patients	608	NR	NR	NR	58 (58–not reached)	Abemaciclib vs palbociclib: 1.29 (0.85-1.96); *P*=0.229 Ribociclib vs palbociclib: 1.04 (0.61-1.77); *P*=0.899	NR
	Abemaciclib + ET	All patients	133	NR		NR	NR (56–not reached)		NR
	Ribociclib + ET	All patients	91	NR		NR	NR		NR

AI, aromatase inhibitor; CDK4/6i, cyclin-dependent kinase 4/6 inhibitors; CI, confidence interval; ET, endocrine therapy; IPTW, inverse probability of treatment weight; NR, not reported; OS, overall survival; PFS, progression-free survival; RWE, real-world evidence.

###### 3.5.1.1.1 Overall population

In these nine comparative studies, median PFS was comparable across CDK4/6i-based regimens. Specifically, PFS ranged from 16.0 (n=NR) (61) to 31.0 months (n=473) (62) for patients receiving palbociclib, 14.0 (n=NR) (61) to 44.0 months (n=38) (62) for those receiving ribociclib, and 14.0 (n=NR) (63) to 39.5 months (n=56) (64) for abemaciclib. Notably, lower median PFS results for palbociclib and ribociclib were reported in a conference poster reporting on initial data from the Canarian Breast Cancer Group of Spain; however, information regarding the methods used, sample sizes, and follow-up times was largely unavailable (61). Omitting this study resulted in a median PFS range of 23.4 (n=61) (17) to 31.0 months (n=473) (62) for palbociclib and 19.8 (n=38) (65) to 44.0 (n=38) (62) for ribociclib. In one study, the PFS rate at 5 years was 20.9%, 32.6%, and 66.8% among palbociclib-, ribociclib-, and abemaciclib-based regimens, respectively (66). However, it should be noted that the sample sizes varied greatly between treatments, with 162 patients receiving palbociclib, 46 receiving ribociclib, and only 19 patients receiving abemaciclib (66). Two studies, the German OPAL study and a real-world UK study, compared palbociclib and ribociclib and showed no statistically significant difference in PFS (65, 67); no comparisons with abemaciclib were available (**Table 4**, **Figure 4**). Quality assessment was performed for four of these studies; three had NOS scores of 7 and were judged of sufficient credibility on the ISPOR questionnaire (63, 64, 66), while the remaining study (68) had a NOS score of 5 and was judged of insufficient quality due to low scores in the credibility, analyses, and interpretation domains of the ISPOR questionnaire.

###### 3.5.1.1.2 Subgroups

Subgroups based on ET response (e.g., *de novo*, recurrent, endocrine-resistant/sensitive) were reported in three studies. Across the different CDK4/6i-based regimens, the median PFS and PFS rates were consistently higher among patients with *de novo* disease than those with recurrent disease (61, 64, 66). For patients with recurrent disease, median PFS ranged from 8.0 (n = NR) (61) to 20.9 months (n = 88) (66) with palbociclib, 6.0 (n = NR) (61) to 18.9 months (n = 34) (66) with ribociclib, and 12.0 months (n = NR) (61) with abemaciclib. One study included endocrine-resistant subgroups and found median PFS was higher among patients receiving the palbociclib regimen (n = 37; 17.0 months) than those receiving the ribociclib regimen (n = 20; 10.4 months) (64).

Additional prespecified subgroups of interest were assessed in the comparative studies that reported PFS data for palbociclib versus ribociclib versus abemaciclib, which included metastases (e.g., visceral), dose reduction, hormonal status (e.g., ER-positive [ER+], PR−), and age. The results of these studies are described in **Supplementary Table 6**, **Appendix I** in the **Supplementary Material**.

Overall, in studies with subgroups of interest data, the quality assessment of two studies indicated they were of reasonably high quality (both with NOS scores of 7 and judged sufficiently credible per the ISPOR questionnaire) (64, 66).

#### 3.5.2 Overall survival

##### 3.5.2.1 Palbociclib versus ribociclib versus abemaciclib

The OS data for patients receiving palbociclib, ribociclib, or abemaciclib were available in seven comparative studies (**Figure 1C**). Three studies reported results for the overall population (**Table 4**, **Figure 4**), while four reported overall and subgroup population data (**Supplementary Table 6**, **Appendix I** in the **Supplementary Material**).

###### 3.5.2.1.1 Overall population

In these seven comparative studies, the data reflect a broadly similar level of OS benefit across the CDK4/6i class. More specifically, median OS ranged from 38.0 (n=120) (68) to 58.0 months (n=608) (60) among patients receiving a palbociclib regimen, with one study reporting a 5-year OS rate of 48.5% (n=162) (66). Similarly, patients on a ribociclib regimen experienced median OS ranging from 40.4 (n=38) (65) to 52.0 months (n=NR) (61), with a 5-year OS rate of 42.3% (n=46) (66). In comparison, OS results for abemaciclib regimens were often not reached or not reported; however, a comparable median OS of 34.3 months was reported in one study (n=44) (68). In the German registry study, OPAL and a real-world UK study, the comparison between palbociclib and ribociclib indicated no statistically significant difference in OS (65, 67) (**Table 4**, **Figure 4**). Where reported, median follow-up ranged from 27.6 (64) to 49.8 months (65) across studies. Quality assessment was performed for three of these studies; two had a NOS score of 7 and were judged to have sufficient credibility on the ISPOR questionnaire (64, 66), whereas the remaining study (68) had a NOS score of 5 and was judged to be of insufficient credibility due to low scores in the credibility, analyses, and interpretation domains of the ISPOR questionnaire.

###### 3.5.2.1.2 Subgroups

Subgroups based on ET response, as described in Section 3.5.1.1.2, were reported in three studies. Across the different CDK4/6i-based regimens, median OS and OS rates were consistently higher among patients with *de novo* disease than those with recurrent disease (61, 65, 66). For patients with recurrent disease, median OS ranged from 22.0 (n=NR) (61) to 48.4 months (n=NR) (65) with palbociclib, 28.0 (n=NR) (61) to 44.6 months (n=34) (66) with ribociclib, and 30.7 months (n=8) (66) with abemaciclib (**Supplementary Table 6**, **Appendix I** in the **Supplementary Material**). Quality assessment of one of these studies indicated it was of reasonably high quality (with a NOS score of 7 and judged sufficiently credible per the ISPOR questionnaire) (66).

Additional prespecified subgroups of interest were assessed in the comparative studies that reported OS data for palbociclib versus ribociclib versus abemaciclib, which included metastases (e.g., visceral), hormonal status (e.g., ER+, PR−), and age. The results of these studies are described in **Supplementary Table 6**, **Appendix I** in the **Supplementary Material**.

## 4 Discussion

As experience treating patients with HR+/HER2− A/MBC in the first-line setting continues to grow, the RWE base should be examined and updated periodically to better understand the patient experience of those treated with a CDK4/6i. Newly identified studies published after an earlier SLR confirm the validity of prior findings and fully inform care and policy development for health care consumers with the latest research (69). Updated reviews also provide the benefit of further guiding future opportunities for research and synthesis as new evidence emerges or new methods develop.

Our previous SLR from 2021 provided a qualitative assessment of 114 eligible RWE studies of approved CDK4/6i in HR+/HER2− A/MBC conducted between 2015 and 2019 across various outcomes related to effectiveness, safety, patient-reported outcomes, and more (11). The overall CDK4/6i evidence base has historically been most established for supporting the real-world effectiveness of palbociclib. However, newer data have emerged for first-line ribociclib and abemaciclib in HR+/HER2− A/MBC, necessitating this updated synthesis of the current RWE landscape of CDK4/6i therapy. Outcomes of interest for this update and the subsequent qualitative synthesis included OS and/or PFS data published since 2019. These two outcomes are widely used in RCTs for evaluating treatment effectiveness and are the primary considerations of oncologists when choosing a specific therapy for patients. Thus, our focused approach provides new insights into the initial use of CDK4/6i in the real world, fleshing out the picture from RCTs, directly informing first-line treatment strategies, and enhancing real-world clinical decision-making for patients with HR+/HER2− A/MBC.

The current synthesis of recently published RWE shows that treating patients with HR+/HER2− A/MBC with CDK4/6i in the first-line setting effectively improves survival outcomes. These results are based on additional data from 82 unique studies spanning almost 5 years since our previously published findings (11). Furthermore, these results corroborate efficacy estimates observed in clinical trials (70). Overall, this updated review captures a greater body of RWE, with the newly included studies encompassing a wider range of study designs (i.e., single-arm and comparative studies), study follow-up times, subgroup population characteristics (e.g., age, racial/ethnic identity, sensitivity to ET, dose reductions, presence of visceral metastases), and additional CDK4/6i effectiveness data spanning several regions in North America, Europe, South America, and Asia. Across the 10 studies comparing different CDK4/6i in the overall population, a total of 1634 patients received palbociclib, 339 patients received ribociclib, and 310 patients received abemaciclib (where reported), with similar effectiveness results. Specifically, the median PFS ranged from 23.4 (17) to 31.0 months (62) for palbociclib, 19.8 (66) to 44.0 months (62) for ribociclib, and 14.0 (63) to 39.5 months (64) for abemaciclib after the exclusion of outlying results from a conference abstract. Furthermore, median OS ranged from 38.0 (68) to 58.0 months (60) for palbociclib, 40.4 (65) to 52.0 months (61) for ribociclib, and 34.4 months in one study for abemaciclib (68). These data highlight an important consideration for assessing longitudinal real-word effectiveness outcomes; estimates may be unreliable if there is a substantial censoring resulting from limited follow up, which needs to be considered as this may lead to an underestimate of survival differences. As for all comparisons, it is important to look at the broader scope of the data; instead of focusing on the median, which is a descriptive statistic reflecting only one point in time, we have focused on the hazard ratios, which take into account the full Kaplan–Meier curves and censoring that are critical to discern the robustness of the outcomes data. These PFS and OS results should be interpreted with caution as the median follow-up times for patients on ribociclib and abemaciclib were consistently shorter than palbociclib (68). Notably, within the included studies for abemaciclib, the available effectiveness data frequently appeared to be lower or not reached. This is likely due to abemaciclib being the most recently approved CDK4/6i; abemaciclib was approved by the US Food and Drug Administration in 2017, and by the European Medicines Agency in 2018 (71, 72), relative to palbociclib approval in 2015 (US) and 2016 (Europe) (73, 74) and ribociclib approval in 2017 (75, 76). There was also a slower initial uptake after approval given reimbursement negotiations as well as greater uptake once the secondary endpoints of survival read out for all CDK4/6i. As a result, the included studies evaluating abemaciclib had comparatively shorter follow-up durations of cohorts. The other key point is the imbalance of patients in each cohort across comparative studies wherein there was a smaller sample of ribociclib and abemaciclib patients at this juncture. However, the vast majority of studies show relatively consistent findings with those observed in single-arm studies and CDK4/6i versus ET comparisons, where CDK4/6i consistently demonstrated greater survival benefits relative to ET alone.

It is critical to note, as indicated by the ISPOR quality assessment, that the comparative studies have significant limitations. For one, the shorter limited follow-up in the abemaciclib and ribociclib cohorts presents an underlying challenge given the later approvals of these CDK4/6i and limited initial uptake. As a result, OS and, in some studies, even PFS, had not reached 50% of events by the time of reporting, making the results unstable until further follow-up. Landmark analyses may be preferred at the earlier time points for comparison purposes, where more patients contributed data. Additionally, most of the studies were descriptive in nature, not controlling for differences in baseline characteristics, such as age or presence of visceral metastases to enable robust comparison of outcomes. Furthermore, the sample size of these studies may preclude the ability to make such comparisons given the relatively limited number of ribociclib and abemaciclib patients as well as in some subgroups. There was also considerable heterogeneity in the clinical variables included and in the level of missing data due to the variety of data sources. It is important to consider this variability, as physician choice between different CDK4/6i is often based on these factors. Lastly, most studies included only select sites, investigators, and patients within a single country, which introduces potential bias and limits the generalizability of the study conclusions. This contrasts with studies that included all eligible patients within an electronic health record database where multiple sites across an entire EHR network are represented. Despite these limitations, 20 studies (87.0%) were identified as being of sufficient credibility due to thorough reporting of study relevance (e.g., population, intervention, and outcomes) and methodology (e.g., statistical analysis and data sources), as well as appropriate descriptions and interpretations of the corresponding results.

Leveraging additional RWE, such as those from comparative studies, may also help guide clinicians towards alternative therapeutic options with comparable effectiveness and safety to CDK4/6i, although the caveats mentioned above need to be considered knowing the selection biases that exist. Despite this, our findings in this updated review were consistent with previously published data based on a different set of studies (11), as well as with efficacy estimates observed in RCTs for these agents (70, 77). Palbociclib with its large body of real-world evidence and the longest follow-up showed generally consistent survival benefit as in RCTs; as more real-world data becomes available for ribociclib and abemaciclib, it will be possible to gain a more complete picture of clinical benefit of CDK4/6i as a class and will also facilitate more robust comparisons between CDK4/6i. Current evidence from comparative studies does not indicate that there is any one CDK4/6i that is better than the other. However, RWE can be used in conjunction with data from RCTs to inform treatment decisions in the clinic, particularly for specific patient populations that may be excluded from RCTs, such as older patients, African-American patients, patients with specific comorbidities or types/numbers of metastases, patients with specific cancer subtypes or genetic signatures, or patients with different treatment-free intervals, to name a few of the subgroups identified in this analysis.

This study has potential limitations. Because the current review only included studies assessing OS and/or PFS, the full effect and benefits of CDK4/6i in real-world settings in terms of therapeutic response, health-related quality of life, and safety were not captured; a targeted review assessing publications across all CDK4/6i found limited safety, quality of life or patient-reported outcome data in the literature (78). A recent SLR assessing evidence from both RCTs and RWE showed that palbociclib was effective, well tolerated, and maintained QoL in older patients with HR+/HER2− A/MBC; clinical benefit profile of palbociclib in real-world settings was consistent with results seen in clinical trials (70). One other recent SLR assessing the impact of palbociclib on patient quality of life found that quality of life is largely maintained while on treatment with palbociclib to ET therapy as assessed in RCTs and RWE (10), but similar large RWE studies have not yet been published for ribociclib or abemaciclib, or for the CDK4/6i class as a whole. Thus, there are future opportunities for synthesis of available RWE evaluating these additional outcomes in populations with HR+/HER2− A/MBC. The follow-up duration of cohorts in the included studies may be insufficient to inform the long-term survival benefits of CDK4/6i use, as some studies had median follow-up times of 18.5 months or less (18, 51, 79, 80), and many studies did not report median follow-up durations. Moreover, the nonrandomized nature of these studies means these results could be affected by confounding factors. It is also important to recognize that there is variability in the rigor and robustness of RWE studies – a majority of studies included in this analysis (59%) has NOS scores of 4 or 5, indicating lower quality of these studies; lack of a comparator arm was the most significant contributor to lower scores. This suggests the need for cautious interpretation when reviewing outcomes and conclusions from these studies, and emphasizes that quality assessment of the published research is an important factor to consider when giving weight to published outcomes.

Finally, the current review only provided a qualitative evaluation of RWE on the effectiveness of CDK4/6i in HR+/HER2− A/MBC. Although inherent to RWE, the inclusion of diverse patient populations, subgroups, and geographical regions results in notable heterogeneity across studies that can affect the generalizability and comparability of findings, it represents patients who may not have been included in clinical trials given strict inclusion/exclusion criteria. Future research may incorporate quantitative analyses to help synthesize data from different sources, account for variations in study design and population, and offer more robust estimates of effectiveness and safety to more accurately guide clinical decision-making.

## 5 Conclusion

Consistent with findings from a previously published review, as well as with CDK4/6i clinical trials, the single-arm and comparative RWE studies included in this updated SLR indicate that first-line CDK4/6i are effective treatments for patients with HR+/HER2− A/MBC with the largest available real-world data reported for palbociclib at this time. With increasing use of CDK4/6i in first-line standard of care for HR+/HER2− A/MBC, we can potentially expect more long-term comparative data to become available in bigger RWE studies. With longer follow-up and larger patient cohorts, the current body of evidence can better inform real-world treatment guidelines and clinical decision-making. These data fill important knowledge gaps from randomized clinical trials and may help guide clinical decision-making for broad patient populations and specific subgroups that may particularly benefit from CDK4/6i therapy.

## 6 Summary points

**Introduction**

- Breast cancer remains the most commonly diagnosed cancer for women.
- The introduction of CDK4/6i changed the treatment landscape for HR+/HER2– A/MBC, resulting in a new standard-of-care.
- The available RWE on the impact of CDK4/6i has increased since the publication of a previous SLR, and now includes real-world data for all three CDK4/6i, although this data is predominantly for palbociclib.

**Materials and methods**

- Literature published since the previous SLR searches was included in this analysis.
- OVID Medline, EMBASE, Cochrane databases, and key clinical congress proceedings were searched.
- Studies were included if they reported RWE in adult patients with HR+/HER2 – A/MBC who received treatment with a CDK4/6i in the first-line setting.
- Studies were excluded if published before 2019, had fewer than 100 patients, or did not specify the line of therapy or a specific CDK4/6i.
- Outcomes of interest were median PFS and/or median OS.
- Studies were categorized by study design, with further stratification by comparator arm if/when possible.
- Data were reported for overall populations and pre-identified subgroups of interest.
- Risk of bias and credibility were assessed using the Newcastle-Ottawa scale, the ISPOR questionnaire, and the ESMO-GROW checklist.

**Results**

- Eighty-two unique studies were included in this qualitative synthesis.
- Most studies (43%) evaluating a single CDK inhibitor were palbociclib studies; 46% of the studies assessed more than one CDK inhibitor.
- In single arm studies, CDK4/6i were generally effective at improving survival outcomes in real-world clinical practice both in a broad population and in subgroups of high clinical interest (eg, older patients, patients with visceral or bone metastases, patients with comorbidities).
- When compared to ET monotherapy, palbociclib plus AI demonstrated improved PFS in broad populations and subgroups; limited data was available for ribociclib plus ET versus ET alone, and no comparative studies for abemaciclib were identified in the SLR.
- In the studies comparing CDK4/6i regimens, the impact on PFS and OS were generally comparable across the three CDK4/6i in the overall population.

**Discussion**

- Since the previous SLR investigating the real-world impact of CDK4/6i was developed, the available pool of RWE has grown.
- This updated synthesis of RWE published since 2019 indicates that CDK4/6i treatment in the first-line setting is effective at improving survival outcomes in patients with HR+/HER2– A/MBC across a wide range of study designs and subgroups of interest; however, the most patients studied had received palbociclib + ET.
- However, these data should be interpreted with caution as there is limited median follow-up time for patients being treated with ribociclib or abemaciclib.
- Additionally, results from RWE studies should be considered in the context of study design, strength of the statistical methods, possible geographical bias, and sample size.
- The studies included in this analysis were largely identified as being of sufficient credibility, and the data are consistent with previously published studies and RCTs.

**Conclusions**

- Consistent with the previous review and RCTs, the published RWE indicates that CDK4/6i are effective first-line treatments for patients with HR+/HER2– A/MBC.
- Longer-term comparative data from larger RWE studies will add to the current body of evidence and provide additional resources to guide clinical decision-making.
