# Supplementary material for: Real-world effectiveness of CDK4/6i in first-line treatment of HR+/HER2− advanced/metastatic breast cancer: updated systematic review
Source: Front Oncol. 2025 Mar 10;15:1530391. doi: 10.3389/fonc.2025.1530391 (PMC11931418; doi:10.3389/fonc.2025.1530391)
Supplement: Supplementary file 1 [file DataSheet1.docx]

**Supplementary Materials**

- **APPENDIX A:** PRISMA 2020 checklist
- **APPENDIX B:** Most recent search strategies
- **APPENDIX C:** List of prespecified subgroups of interest
- **APPENDIX D:** PRISMA flow diagrams
- **APPENDIX E:** List of included studies
- **APPENDIX F:** Quality assessment
- **APPENDIX G:** Effectiveness of any first-line CDK4/6i regimen in single-arm studies
- **APPENDIX H:** Effectiveness outcomes for first-line CDK4/6i in single-arm RWE studies
- A**PPENDIX I:** Effectiveness outcomes for first-line CDK4/6i in subgroup populations
- **APPENDIX J:** Effectiveness of any first-line CDK4/6i regimen vs ET in comparative studies
- **APPENDIX K:** Effectiveness of CDK4/6i versus CDK4/6i: Comparative results of CDK4/6i and different backbone therapies
- **APPENDIX L:** Supplementary references

APPENDIX A: PRISMA 2020 checklist ^1^

| **Section and topic** | **Item #** | **Checklist item** | **Location where item is reported** |
| --- | --- | --- | --- |
| **Title** | | |  |
| Title | 1 | Identify the report as a systematic review. | Pg 1 |
| **Abstract** | | |  |
| Abstract | 2 | See the PRISMA 2020 for Abstracts checklist. | Pg 2 |
| **Introduction** | | |  |
| Rationale | 3 | Describe the rationale for the review in the context of existing knowledge. | Pg 3-4 |
| Objectives | 4 | Provide an explicit statement of the objective(s) or question(s) the review addresses. | Pg 4 |
| **Methods** | | |  |
| Eligibility criteria | 5 | Specify the inclusion and exclusion criteria for the review and how studies were grouped for the syntheses. | Pg 4-5 |
| Information sources | 6 | Specify all databases, registers, websites, organizations, reference lists, and other sources searched or consulted to identify studies. Specify the date when each source was last searched or consulted. | Pg 4 |
| Search strategy | 7 | Present the full search strategies for all databases, registers, and websites, including any filters and limits used. | Appendix B |
| Selection process | 8 | Specify the methods used to decide whether a study met the inclusion criteria of the review, including how many reviewers screened each record and each report retrieved, whether they worked independently, and, if applicable, details of automation tools used in the process. | Pg 4-5 |
| Data collection process | 9 | Specify the methods used to collect data from reports, including how many reviewers collected data from each report, whether they worked independently, any processes for obtaining or confirming data from study investigators, and, if applicable, details of automation tools used in the process. | Pg 4-5 |
| Data items | 10a | List and define all outcomes for which data were sought. Specify whether all results that were compatible with each outcome domain in each study were sought (e.g., for all measures, time points, analyses), and if not, the methods used to decide which results to collect. | Pg 5 |
|  | 10b | List and define all other variables for which data were sought (e.g., participant and intervention characteristics, funding sources). Describe any assumptions made about any missing or unclear information. | Pg 5, Appendix C |
| Study risk of bias assessment | 11 | Specify the methods used to assess risk of bias in the included studies, including details of the tool(s) used, how many reviewers assessed each study and whether they worked independently, and if applicable, details of automation tools used in the process. | Pg 6 |
| Effect measures | 12 | Specify for each outcome the effect measure(s) (e.g., risk ratio, mean difference) used in the synthesis or presentation of results. | Pg 5 |
| Synthesis methods | 13a | Describe the processes used to decide which studies were eligible for each synthesis (e.g., tabulating the study intervention characteristics and comparing against the planned groups for each synthesis (item #5)). | Pg 5 |
|  | 13b | Describe any methods required to prepare the data for presentation or synthesis, such as handling of missing summary statistics or data conversions. | n/a |
|  | 13c | Describe any methods used to tabulate or visually display results of individual studies and syntheses. | Pg 5 |
|  | 13d | Describe any methods used to synthesize results and provide a rationale for the choice(s). If meta-analysis was performed, describe the model(s), method(s) to identify the presence and extent of statistical heterogeneity, and software package(s) used. | Pg 5, 17-18 |
|  | 13e | Describe any methods used to explore possible causes of heterogeneity among study results (e.g., subgroup analysis, meta-regression). | n/a |
|  | 13f | Describe any sensitivity analyses conducted to assess robustness of the synthesized results. | n/a |
| Reporting bias assessment | 14 | Describe any methods used to assess risk of bias due to missing results in a synthesis (arising from reporting biases). | n/a |
| Certainty assessment | 15 | Describe any methods used to assess certainty (or confidence) in the body of evidence for an outcome. | Pg 7 |
| **Results** | | |  |
| Study selection | 16a | Describe the results of the search and selection process, from the number of records identified in the search to the number of studies included in the review, ideally using a flow diagram. | Pg 6 |
|  | 16b | Cite studies that might appear to meet the inclusion criteria but which were excluded, and explain why they were excluded. | Appendix D |
| Study characteristics | 17 | Cite each included study and present its characteristics. | Appendix E |
| Risk of bias in studies | 18 | Present assessments of risk of bias for each included study. | Appendix F |
| Results of individual studies | 19 | For all outcomes, present, for each study: (a) summary statistics for each group (where appropriate) and (b) an effect estimate and its precision (e.g., confidence/credible interval), ideally using structured tables or plots. | n/a |
| Results of syntheses | 20a | For each synthesis, briefly summarise the characteristics and risk of bias among contributing studies. | Pg 7, Appendices G-K |
|  | 20b | Present results of all statistical syntheses conducted. If meta-analysis was done, present for each the summary estimate and its precision (e.g., confidence/credible interval) and measures of statistical heterogeneity. If comparing groups, describe the direction of the effect. | n/a |
|  | 20c | Present results of all investigations of possible causes of heterogeneity among study results. | Pg 19 |
|  | 20d | Present results of all sensitivity analyses conducted to assess the robustness of the synthesized results. | n/a |
| Reporting biases | 21 | Present assessments of risk of bias due to missing results (arising from reporting biases) for each synthesis assessed. | Pg 7-17 |
| Certainty of evidence | 22 | Present assessments of certainty (or confidence) in the body of evidence for each outcome assessed. | Pg 7-17 |
| **Discussion** | | |  |
| Discussion | 23a | Provide a general interpretation of the results in the context of other evidence. | Pg 18 |
|  | 23b | Discuss any limitations of the evidence included in the review. | Pg 18 |
|  | 23c | Discuss any limitations of the review processes used. | n/a |
|  | 23d | Discuss implications of the results for practice, policy, and future research. | Pg 19-21 |
| **Other information** | | |  |
| Registration and protocol | 24a | Provide registration information for the review, including register name and registration number, or state that the review was not registered. | Pg 4 |
|  | 24b | Indicate where the review protocol can be accessed or state that a protocol was not prepared. | Pg 26 |
|  | 24c | Describe and explain any amendments to information provided at registration or in the protocol. | n/a |
| Support | 25 | Describe sources of financial or nonfinancial support for the review and the role of the funders or sponsors in the review. | Pg 26 |
| Competing interests | 26 | Declare any competing interests of review authors. | Pg 26 |
| Availability of data, code. and other materials | 27 | Report which of the following are publicly available and where they can be found: template data collection forms; data extracted from included studies; data used for all analyses; analytic code; any other materials used in the review. | Pg 26 |

For more information, visit: <http://www.prisma-statement.org/>

APPENDIX B: Most recent search strategy

**Date of the search:** 9 Jan 2024

**Update date span:** Oct 2023 - Current (based on the update dates of the included databases, CENTRAL & CDSR update: Sept 2022 - Current)

**Databases searched:**

Ovid MEDLINE(R) and Epub Ahead of Print, In-Process & Other Non-Indexed Citations and Daily

Ovid EMBASE

Ovid EBM Reviews - Cochrane Central Register of Controlled Trials

Ovid EBM Reviews - Cochrane Database of Systematic Reviews

**Summary of Results:**

| **Search** | **Ovid Results** | **Results deduped in Ovid** | **Results deduped in EndNote** |
| --- | --- | --- | --- |
| CDK4/6i SLR Update Oct 2023 - Current | 708 | 578 | 572 |

Search history saved in **Corner3** as: "Breast Cancer - Palbociclib - Multifile - UPDATE 6 -120 000443- FINAL v3"

09 Jan 2024 - UPDATE #6 timespan: Oct 2023 (Sept 2022 for CENTRAL & CDSR) - Current

**MULTIFILE SEARCH**

Database(s): **EBM Reviews - Cochrane Central Register of Controlled Trials**December 2023**, EBM Reviews - Cochrane Database of Systematic Reviews**2005 to January 3, 2024**, Embase**1974 to 2024 January 08**, Ovid MEDLINE(R) and Epub Ahead of Print, In-Process, In-Data-Review & Other Non-Indexed Citations and Daily**1946 to January 08, 2024

Search Strategy:

| **#** | **Searches** | **Results** |
| --- | --- | --- |
| 1 | exp Breast Neoplasms/ | 1029163 |
| 2 | exp Breast Carcinoma In Situ/ | 15846 |
| 3 | ((breast$1 or mamma or mammary) adj3 (adenocarcinoma* or cancer* or carcinoma* or neoplasm* or tumour* or tumor*)).tw,kf. | 1085445 |
| 4 | ((ductal or intraductal or intra-ductal) adj (carcinoma? or hyperplasia?)).tw,kf. | 49610 |
| 5 | DCIS.tw,kf. | 18207 |
| 6 | (lobul* carcinoma? adj2 "in situ").tw,kf. | 2992 |
| 7 | LCIS.tw,kf. | 1951 |
| 8 | (paget* and (areola? or breast* or mammary or nipple*)).tw,kf. | 3442 |
| 9 | or/1-8 [BREAST CANCER] | 1309948 |
| 10 | Receptor, ErbB-2/ | 107636 |
| 11 | ERBB2 protein, human.nm. | 12851 |
| 12 | (ErbB2 or "ErbB 2" or HER2* or "HER 2*" or "c-ErbB2" or "C-ErbB 2").tw,kf. | 165534 |
| 13 | ((oncoprotein* or onco-protein* or protein* or receptor*) adj1 (neu or neuregulin)).tw,kf. | 3235 |
| 14 | CD340.tw,kf. | 49 |
| 15 | ("p185(c-neu)" or p185erbB).tw,kf. | 90 |
| 16 | (neu protooncogene or neu proto-oncogene).tw,kf. | 454 |
| 17 | NGL.tw,kf. | 1962 |
| 18 | metastatic lymph node gene 19.tw,kf. | 0 |
| 19 | (MLN19 or MLN 19).tw,kf. | 2 |
| 20 | (human adj1 "epidermal growth factor receptor 2").tw,kf. | 27941 |
| 21 | ErbB Receptors/ | 156368 |
| 22 | or/10-21 | 333450 |
| 23 | 9 and 22 [HER2 BREAST CANCER] | 149931 |
| 24 | exp Receptors, Estrogen/ | 169215 |
| 25 | estrogen receptor?.tw,kf. | 146412 |
| 26 | oestrogen receptor?.tw,kf. | 16112 |
| 27 | ERalpha*.tw,kf. | 29345 |
| 28 | ERbeta.tw,kf. | 15152 |
| 29 | ER positive.tw,kf. | 20841 |
| 30 | "luminal a".tw,kf. | 10276 |
| 31 | "luminal b".tw,kf. | 8689 |
| 32 | (luminal adj2 subtype*).tw,kf. | 6495 |
| 33 | hormone receptor?.tw,kf. | 93480 |
| 34 | HR positive.tw,kf. | 4531 |
| 35 | progesterone receptor?.tw,kf. | 59555 |
| 36 | progestin receptor?.tw,kf. | 1925 |
| 37 | PR positive.tw,kf. | 4286 |
| 38 | or/24-37 | 332470 |
| 39 | 9 and 38 [HR BREAST CANCER] | 169470 |
| 40 | exp Breast Neoplasms/sc [secondary/metastatic] | 5262 |
| 41 | (advanced adj3 ((breast$1 or mamma or mammary) adj3 (adenocarcinoma* or cancer* or carcinoma* or neoplasm* or tumour* or tumor*))).tw,kf. | 40140 |
| 42 | (metastatic* adj3 ((breast$1 or mamma or mammary) adj3 (adenocarcinoma* or cancer* or carcinoma* or neoplasm* or tumour* or tumor*))).tw,kf. | 73332 |
| 43 | (metastas* adj3 ((breast$1 or mamma or mammary) adj3 (adenocarcinoma* or cancer* or carcinoma* or neoplasm* or tumour* or tumor*))).tw,kf. | 39821 |
| 44 | (progressive adj3 ((breast$1 or mamma or mammary) adj3 (adenocarcinoma* or cancer* or carcinoma* or neoplasm* or tumour* or tumor*))).tw,kf. | 658 |
| 45 | (secondary adj3 ((breast$1 or mamma or mammary) adj3 (adenocarcinoma* or cancer* or carcinoma* or neoplasm* or tumour* or tumor*))).tw,kf. | 2803 |
| 46 | (terminal adj3 ((breast$1 or mamma or mammary) adj3 (adenocarcinoma* or cancer* or carcinoma* or neoplasm* or tumour* or tumor*))).tw,kf. | 171 |
| 47 | or/40-46 [METASTATIC BREAST CANCER] | 140337 |
| 48 | 23 or 39 or 47 [BREAST CANCERS OF INTEREST] | 344074 |
| 49 | exp Infant/ not exp Adult/ | 1857551 |
| 50 | exp Child/ not exp Adult/ | 3735929 |
| 51 | Adolescent/ not exp Adult/ | 1408289 |
| 52 | 48 not (49 or 50 or 51) [INFANT-, CHILD-, ADOLESCENT-ONLY REMOVED] | 343239 |
| 53 | Palbociclib.nm. | 956 |
| 54 | (palbociclib or ibrance$2 or palbociclib or "pd 0332991" or "pd 0332991" or pd332991or pd332991 or "pf 00080665" or pf00080665).tw,kf. | 7202 |
| 55 | ribociclib.nm. | 344 |
| 56 | (ribociclib or kisqali$2 or "lee 011" or lee 011a or lee 011bba or lee 11 or lee 11a or lee 11bba or lee011 or lee011a or lee011bba or lee11 or lee11a or lee11bba).tw,kf. | 3050 |
| 57 | (abemaciclib or bemaciclib$2 or ly 2835219 or ly2835219 or verzenio$2).tw,kf. | 3046 |
| 58 | Cyclin-Dependent Kinase Inhibitor Proteins/ | 9206 |
| 59 | cyclin-dependent kinase inhibitor?.tw,kf. | 16182 |
| 60 | (CDKI or CDKIs).tw,kf. | 1532 |
| 61 | CKI Protein?.tw,kf. | 47 |
| 62 | (CIP-KIP adj2 protein?).tw,kf. | 85 |
| 63 | Cyclin-Dependent Kinases/ | 22674 |
| 64 | Cyclin-Dependent Kinase 4/ | 20275 |
| 65 | Cyclin-Dependent Kinase 6/ | 11962 |
| 66 | (Cyclin-Dependent Kinase 4 or Cyclin-Dependent Kinase 6).tw,kf. | 6075 |
| 67 | (CDK4* or CDK 4* or CDK6* or CDK 6*).tw,kf. | 32198 |
| 68 | (Cell Division Protein Kinase 4 or PSK-J3 Kinase or PSKJ3 Kinase or p34PSK-J3 Kinase or p34PSKJ3 Kinase).tw,kf. | 6 |
| 69 | (Cell Division Protein Kinase 6 or PLSTIRE Protein).tw,kf. | 12 |
| 70 | or/53-69 [FIRST LINE DRUGS] | 78591 |
| 71 | 52 and 70 [BREAST CANCERS OF INTEREST - FIRST LINE DRUGS] | 10739 |
| 72 | exp Animals/ not Humans/ | 16902871 |
| 73 | 71 not 72 [ANIMAL-ONLY REMOVED] | 9919 |
| 74 | limit 73 to yr="2023-current" | 1928 |
| 75 | 74 use ppez [MEDLINE RECORDS] | 523 |
| 76 | exp breast cancer/ | 948461 |
| 77 | ((breast$1 or mamma or mammary) adj3 (adenocarcinoma* or cancer* or carcinoma* or neoplasm* or tumour* or tumor*)).tw,kw. | 1070247 |
| 78 | ((ductal or intraductal or intra-ductal) adj (carcinoma? or hyperplasia?)).tw,kw. | 48962 |
| 79 | DCIS.tw,kw. | 18130 |
| 80 | (lobul* carcinoma? adj2 "in situ").tw,kw. | 2920 |
| 81 | LCIS.tw,kw. | 1942 |
| 82 | (paget* and (areola? or breast* or mammary or nipple*)).tw,kw. | 3455 |
| 83 | or/76-82 [BREAST CANCER] | 1279169 |
| 84 | epidermal growth factor receptor 2/ | 80055 |
| 85 | (ErbB2 or "ErbB 2" or HER2* or "HER 2*" or "c-ErbB2" or "C-ErbB 2").tw,kw. | 165364 |
| 86 | ((oncoprotein* or onco-protein* or protein* or receptor*) adj1 (neu or neuregulin)).tw,kw. | 3223 |
| 87 | CD340.tw,kw. | 49 |
| 88 | ("p185(c-neu)" or p185erbB).tw,kw. | 90 |
| 89 | (neu protooncogene or neu proto-oncogene).tw,kw. | 453 |
| 90 | NGL.tw,kw. | 1957 |
| 91 | metastatic lymph node gene 19 protein*.tw,kw. | 0 |
| 92 | (MLN19 or MLN 19).tw,kw. | 2 |
| 93 | (human adj1 "epidermal growth factor receptor 2").tw,kw. | 28093 |
| 94 | or/84-93 | 193980 |
| 95 | 83 and 94 [HER2 BREAST CANCER] | 137033 |
| 96 | exp estrogen receptor/ | 169215 |
| 97 | estrogen receptor?.tw,kw. | 145488 |
| 98 | oestrogen receptor?.tw,kw. | 15985 |
| 99 | ERalpha*.tw,kw. | 29320 |
| 100 | ERbeta.tw,kw. | 15122 |
| 101 | exp estrogen receptor positive breast cancer/ | 12198 |
| 102 | ER positive.tw,kw. | 20788 |
| 103 | "luminal a".tw,kw. | 10275 |
| 104 | "luminal b".tw,kw. | 8685 |
| 105 | (luminal adj2 subtype*).tw,kw. | 6441 |
| 106 | hormone receptor?.tw,kw. | 91091 |
| 107 | progesterone receptor positive breast cancer/ | 2165 |
| 108 | HR positive.tw,kw. | 4497 |
| 109 | progesterone receptor?.tw,kw. | 59314 |
| 110 | PR positive.tw,kw. | 4276 |
| 111 | progestin receptor?.tw,kw. | 1919 |
| 112 | or/96-111 | 332793 |
| 113 | 83 and 112 [HR BREAST CANCER] | 171392 |
| 114 | metastatic breast cancer/ | 17223 |
| 115 | (advanced adj3 ((breast$1 or mamma or mammary) adj3 (adenocarcinoma* or cancer* or carcinoma* or neoplasm* or tumour* or tumor*))).tw,kw. | 39888 |
| 116 | (metastatic* adj3 ((breast$1 or mamma or mammary) adj3 (adenocarcinoma* or cancer* or carcinoma* or neoplasm* or tumour* or tumor*))).tw,kw. | 72798 |
| 117 | (metastas* adj3 ((breast$1 or mamma or mammary) adj3 (adenocarcinoma* or cancer* or carcinoma* or neoplasm* or tumour* or tumor*))).tw,kw. | 40009 |
| 118 | (progressive adj3 ((breast$1 or mamma or mammary) adj3 (adenocarcinoma* or cancer* or carcinoma* or neoplasm* or tumour* or tumor*))).tw,kw. | 658 |
| 119 | (secondary adj3 ((breast$1 or mamma or mammary) adj3 (adenocarcinoma* or cancer* or carcinoma* or neoplasm* or tumour* or tumor*))).tw,kw. | 2770 |
| 120 | (terminal adj3 ((breast$1 or mamma or mammary) adj3 (adenocarcinoma* or cancer* or carcinoma* or neoplasm* or tumour* or tumor*))).tw,kw. | 174 |
| 121 | or/114-120 [METASTATIC BREAST CANCER] | 140684 |
| 122 | 95 or 113 or 121 [BREAST CANCERS OF INTEREST] | 336637 |
| 123 | adolescent/ not exp adult/ | 1408289 |
| 124 | child/ not exp adult/ | 2699095 |
| 125 | infant/ not exp adult/ | 1176104 |
| 126 | fetus/ not exp adult/ | 244311 |
| 127 | 122 not (123 or 124 or 125 or 126) [FETUS-, INFANT-, CHILD-, ADOLESCENT-ONLY REMOVED] | 335859 |
| 128 | palbociclib/ | 7133 |
| 129 | (palbociclib or ibrance$2 or palbociclib or "pd 0332991" or "pd 0332991" or pd332991or pd332991 or "pf 00080665" or pf00080665).tw,kw. | 7196 |
| 130 | ribociclib/ | 2921 |
| 131 | (ribociclib or kisqali$2 or "lee 011" or lee 011a or lee 011bba or lee 11 or lee 11a or lee 11bba or lee011 or lee011a or lee011bba or lee11 or lee11a or lee11bba).tw,kw. | 3045 |
| 132 | abemaciclib/ | 3120 |
| 133 | (abemaciclib or bemaciclib$2 or ly 2835219 or ly2835219 or verzenio$2).tw,kw. | 3042 |
| 134 | cyclin dependent kinase inhibitor/ | 8439 |
| 135 | cyclin-dependent kinase inhibitor?.tw,kw. | 15939 |
| 136 | (CDKI or CDKIs).tw,kw. | 1512 |
| 137 | CKI protein?.tw,kw. | 47 |
| 138 | (CIP-KIP adj2 protein?).tw,kw. | 84 |
| 139 | cyclin dependent kinase/ | 23120 |
| 140 | cyclin dependent kinase 4/ | 20275 |
| 141 | cyclin dependent kinase 6/ | 11962 |
| 142 | (Cyclin-Dependent Kinase 4 or Cyclin-Dependent Kinase 6).tw,kw. | 5974 |
| 143 | (CDK4* or CDK 4* or CDK6* or CDK 6*).tw,kw. | 32182 |
| 144 | (Cell Division Protein Kinase 4 or PSK-J3 Kinase or PSKJ3 Kinase or p34PSK-J3 Kinase or p34PSKJ3 Kinase).tw,kw. | 6 |
| 145 | (Cell Division Protein Kinase 6 or PLSTIRE Protein).tw,kw. | 12 |
| 146 | or/128-145 [FIRST LINE DRUGS] | 80700 |
| 147 | 127 and 146 [BREAST CANCERS OF INTEREST - FIRST LINE DRUGS] | 11469 |
| 148 | exp animal/ or exp animal experimentation/ or exp animal model/ or exp animal experiment/ or nonhuman/ or exp vertebrate/ | 61019819 |
| 149 | exp human/ or exp human experimentation/ or exp human experiment/ | 48486809 |
| 150 | 148 not 149 | 12535021 |
| 151 | 147 not 150 [ANIMAL-ONLY REMOVED] | 11208 |
| 152 | limit 151 to yr="2023-current" | 2090 |
| 153 | 152 use oemezd [EMBASE RECORDS] | 1412 |
| 154 | exp Breast Neoplasms/ | 1029163 |
| 155 | exp Breast Carcinoma In Situ/ | 15846 |
| 156 | ((breast$1 or mamma or mammary) adj3 (adenocarcinoma* or cancer* or carcinoma* or neoplasm* or tumour* or tumor*)).ti,ab,kw. | 1069857 |
| 157 | ((ductal or intraductal or intra-ductal) adj (carcinoma? or hyperplasia?)).ti,ab,kw. | 48936 |
| 158 | DCIS.ti,ab,kw. | 18110 |
| 159 | (lobul* carcinoma? adj2 "in situ").ti,ab,kw. | 2909 |
| 160 | LCIS.ti,ab,kw. | 1934 |
| 161 | (paget* and (areola? or breast* or mammary or nipple*)).ti,ab,kw. | 3445 |
| 162 | or/154-161 [BREAST CANCER] | 1304645 |
| 163 | Receptor, ErbB-2/ | 107636 |
| 164 | (ERBB2 protein adj1 human).mp. | 14454 |
| 165 | (ErbB2 or "ErbB 2" or HER2* or "HER 2*" or "c-ErbB2" or "C-ErbB 2").ti,ab,kw. | 165301 |
| 166 | ((oncoprotein* or onco-protein* or protein* or receptor*) adj1 (neu or neuregulin)).ti,ab,kw. | 3220 |
| 167 | CD340.ti,ab,kw. | 49 |
| 168 | ("p185(c-neu)" or p185erbB).ti,ab,kw. | 90 |
| 169 | (neu protooncogene or neu proto-oncogene).ti,ab,kw. | 453 |
| 170 | NGL.ti,ab,kw. | 1955 |
| 171 | metastatic lymph node gene 19.ti,ab,kw. | 0 |
| 172 | (MLN19 or MLN 19).ti,ab,kw. | 2 |
| 173 | (human adj1 "epidermal growth factor receptor 2").ti,ab,kw. | 28069 |
| 174 | ErbB Receptors/ | 156368 |
| 175 | or/163-174 | 333294 |
| 176 | 162 and 175 [HER2 BREAST CANCER] | 149628 |
| 177 | exp Receptors, Estrogen/ | 169215 |
| 178 | estrogen receptor?.ti,ab,kw. | 145456 |
| 179 | oestrogen receptor?.ti,ab,kw. | 15912 |
| 180 | ERalpha*.ti,ab,kw. | 29320 |
| 181 | ERbeta.ti,ab,kw. | 15120 |
| 182 | ER positive.ti,ab,kw. | 20770 |
| 183 | "luminal a".ti,ab,kw. | 10271 |
| 184 | "luminal b".ti,ab,kw. | 8681 |
| 185 | (luminal adj2 subtype*).ti,ab,kw. | 6439 |
| 186 | hormone receptor?.ti,ab,kw. | 91040 |
| 187 | HR positive.ti,ab,kw. | 4495 |
| 188 | progesterone receptor?.ti,ab,kw. | 59252 |
| 189 | progestin receptor?.ti,ab,kw. | 1918 |
| 190 | PR positive.ti,ab,kw. | 4271 |
| 191 | or/177-190 | 329702 |
| 192 | 162 and 191 [HR BREAST CANCER] | 168871 |
| 193 | exp Breast Neoplasms/sc [secondary/metastatic] | 5262 |
| 194 | (advanced adj3 ((breast$1 or mamma or mammary) adj3 (adenocarcinoma* or cancer* or carcinoma* or neoplasm* or tumour* or tumor*))).ti,ab,kw. | 39846 |
| 195 | (metastatic* adj3 ((breast$1 or mamma or mammary) adj3 (adenocarcinoma* or cancer* or carcinoma* or neoplasm* or tumour* or tumor*))).ti,ab,kw. | 72757 |
| 196 | (metastas* adj3 ((breast$1 or mamma or mammary) adj3 (adenocarcinoma* or cancer* or carcinoma* or neoplasm* or tumour* or tumor*))).ti,ab,kw. | 39998 |
| 197 | (progressive adj3 ((breast$1 or mamma or mammary) adj3 (adenocarcinoma* or cancer* or carcinoma* or neoplasm* or tumour* or tumor*))).ti,ab,kw. | 657 |
| 198 | (secondary adj3 ((breast$1 or mamma or mammary) adj3 (adenocarcinoma* or cancer* or carcinoma* or neoplasm* or tumour* or tumor*))).ti,ab,kw. | 2764 |
| 199 | (terminal adj3 ((breast$1 or mamma or mammary) adj3 (adenocarcinoma* or cancer* or carcinoma* or neoplasm* or tumour* or tumor*))).ti,ab,kw. | 174 |
| 200 | or/193-199 [METASTATIC BREAST CANCER] | 139968 |
| 201 | 176 or 192 or 200 [BREAST CANCERS OF INTEREST] | 343199 |
| 202 | exp Infant/ not exp Adult/ | 1857551 |
| 203 | exp Child/ not exp Adult/ | 3735929 |
| 204 | Adolescent/ not exp Adult/ | 1408289 |
| 205 | 201 not (202 or 203 or 204) [INFANT-, CHILD-, ADOLESCENT-ONLY REMOVED] | 342365 |
| 206 | Palbociclib.mp. | 9922 |
| 207 | (palbociclib or ibrance$2 or palbociclib or "pd 0332991" or "pd 0332991" or pd332991or pd332991 or "pf 00080665" or pf00080665).ti,ab,kw. | 6696 |
| 208 | ribociclib.mp. | 4211 |
| 209 | (ribociclib or kisqali$2 or "lee 011" or lee 011a or lee 011bba or lee 11 or lee 11a or lee 11bba or lee011 or lee011a or lee011bba or lee11 or lee11a or lee11bba).ti,ab,kw. | 2832 |
| 210 | abemaciclib.mp. | 4422 |
| 211 | (abemaciclib or bemaciclib$2 or ly 2835219 or ly2835219 or verzenio$2).ti,ab,kw. | 2892 |
| 212 | Cyclin-Dependent Kinase Inhibitor Proteins/ | 9206 |
| 213 | cyclin-dependent kinase inhibitor?.ti,ab,kw. | 15936 |
| 214 | (CDKI or CDKIs).ti,ab,kw. | 1504 |
| 215 | CKI protein?.ti,ab,kw. | 47 |
| 216 | (CIP-KIP adj2 protein?).ti,ab,kw. | 84 |
| 217 | Cyclin-Dependent Kinases/ | 22674 |
| 218 | Cyclin-Dependent Kinase 4/ | 20275 |
| 219 | Cyclin-Dependent Kinase 6/ | 11962 |
| 220 | (Cyclin-Dependent Kinase 4 or Cyclin-Dependent Kinase 6).ti,ab,kw. | 5973 |
| 221 | (CDK4* or CDK 4* or CDK6* or CDK 6*).ti,ab,kw. | 32176 |
| 222 | (Cell Division Protein Kinase 4 or PSK-J3 Kinase or PSKJ3 Kinase or p34PSK-J3 Kinase or p34PSKJ3 Kinase).ti,ab,kw. | 6 |
| 223 | (Cell Division Protein Kinase 6 or PLSTIRE Protein).ti,ab,kw. | 12 |
| 224 | or/206-223 [FIRST LINE DRUGS] | 80738 |
| 225 | 205 and 224 [BREAST CANCERS OF INTEREST - FIRST LINE DRUGS] | 11403 |
| 226 | 225 use coch,cctr [COCHRANE RECORDS] | 1221 |
| 227 | 75 or 153 or 226 [ALL DATABASES] | 3156 |
| 228 | limit 75 to dt="20231001-20241231" [Limit not valid in CCTR,CDSR,Embase; records were retained] | 135 |
| 229 | limit 153 to dc="20231001-20241231" [Limit not valid in CCTR,CDSR; records were retained] | 508 |
| 230 | 226 and (202309$ or 202310$ or 202311$ or 202312$ or 2024$).up. | 65 |
| 231 | 228 or 229 or 230 | 708 |
| 232 | remove duplicates from 231 | 578 |

EndNote deduplication process: **6** duplicates removed

APPENDIX C: List of prespecified subgroups of interest

| 1. Metastases (e.g., visceral, bone, liver, etc.) |
| --- |
| 1. ET response (e.g., *de novo*, endocrine-resistant, endocrine-sensitive) |
| 1. Hormonal status (e.g., HER2 0, HER2 +1, ER/PR strong, ER/PR weak, etc.) |
| 1. Dose modification/reduction |
| 1. Age (e.g., older adults) |
| 1. Race/ethnicity (e.g., BIPOC) |
| 1. ECOG score |
| 1. Comorbidity (e.g., specific disorders, CCI)/risk factors |
| 1. CDK4/6i starting dose (e.g., palbociclib 125mg, palbociclib <125mg) |
| 1. Menopausal status |

BIPOC = Black, Indigenous, and People of Color; CCI = Charlson Comorbidity Index; CDK4/6i = cyclin-dependent kinase 4/6 inhibitors; ECOG = Eastern Cooperative Oncology Group; ET = endocrine therapy; ER = endocrine receptor; HER = Human Epidermal Growth Factor Receptor; PR = progesterone receptor.

APPENDIX D: PRISMA flow diagrams

January 6, 2023 Update


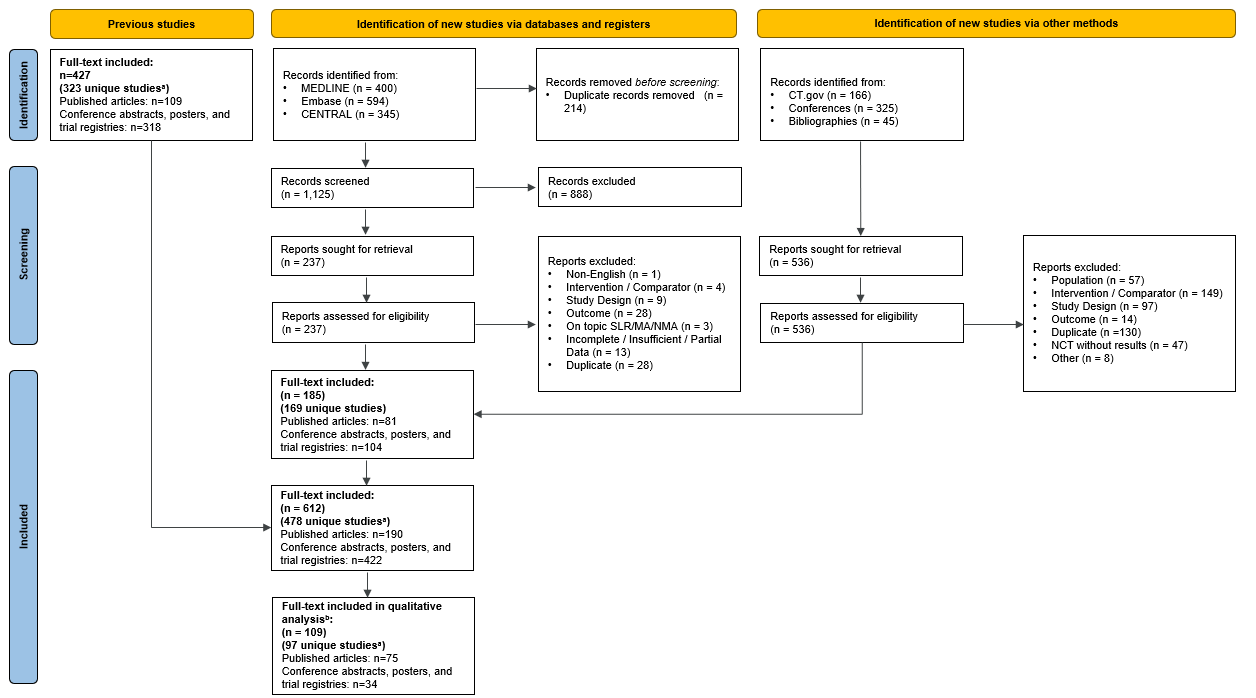


*The January 6, 2023 update includes records from the October 7, 2020, June 1, 2021, and December 1, 2021 searches.

^a^Note that references related to 14 unique studies were captured in the previous SLRs and the December 1, 2021 search. Therefore, the total number of unique studies is 478 when records from all SLRs are combined.

^b^The qualitative analysis included articles published in 2020 onwards and conference abstracts and posters published in 2022. Studies were excluded from the analysis if they had sample sizes of fewer than 100 patients and did not specify the line of therapy.

MA = meta-analysis; NCT = National Clinical Trial; PRISMA = Preferred Reporting Items for Systematic Reviews and Meta-Analyses; SLR = systematic literature review.

October 18, 2023 Update


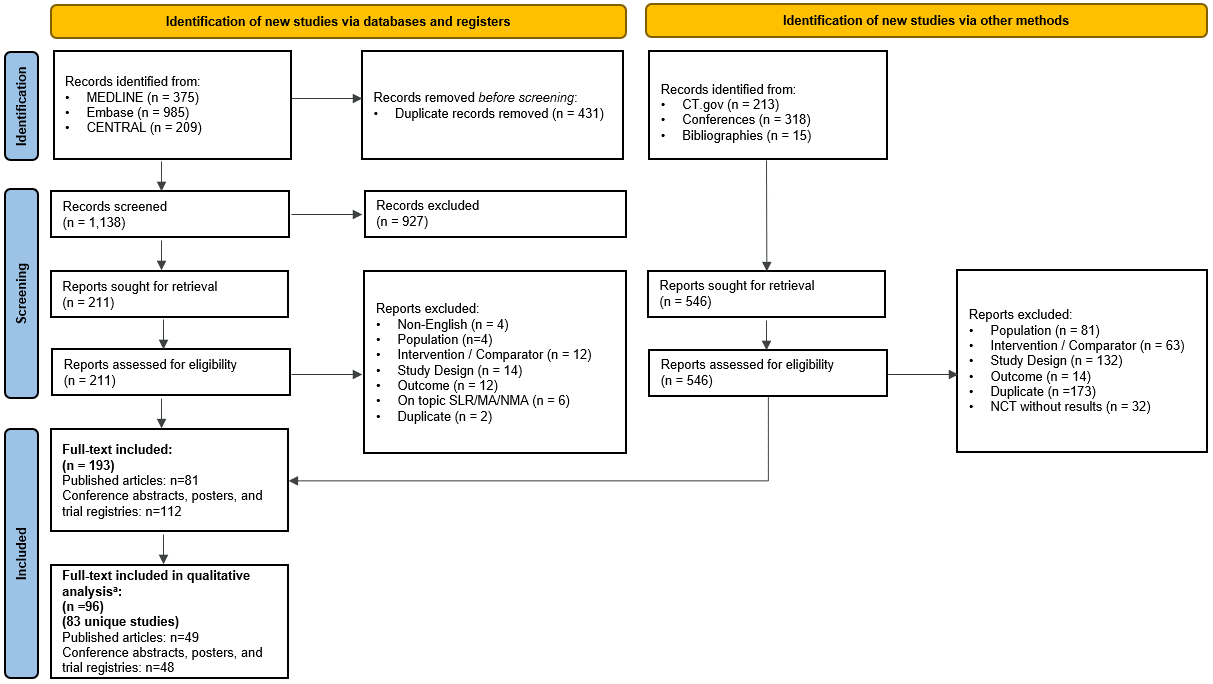


^a^Studies were excluded from the analysis if they had sample sizes less than 100 patients and did not specify the line of therapy or type of CDK4/6i assessed.

CDK4/6i = cyclin-dependent kinase 4/6 inhibitors; MA = meta-analysis; NCT = National Clinical Trial; PRISMA = Preferred Reporting Items for Systematic Reviews and Meta-Analyses; SLR = systematic literature review.

January 9, 2024 Update


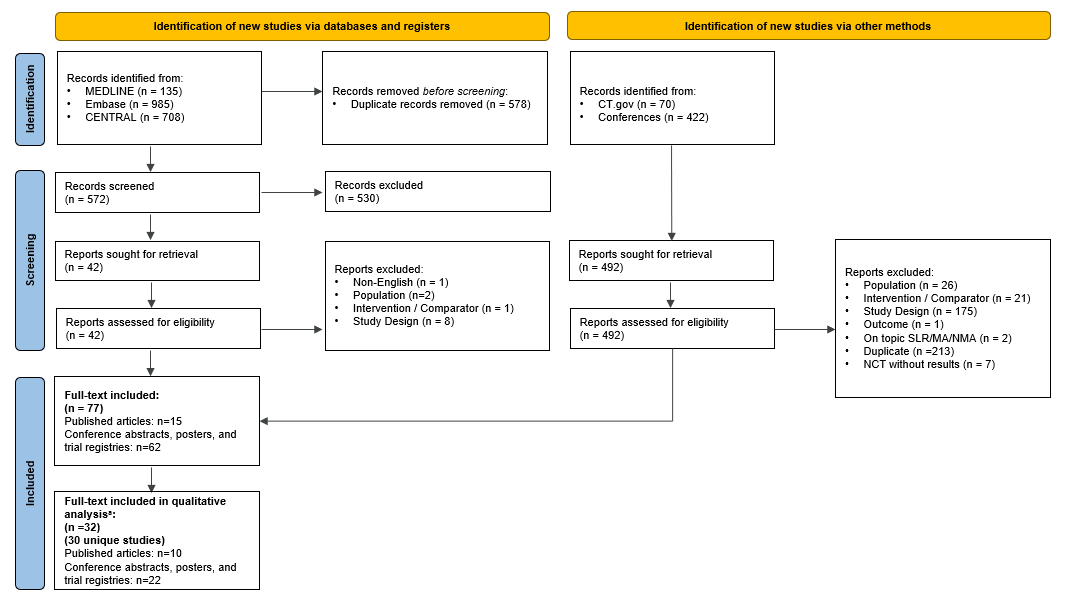


^a^ Studies were excluded from the analysis if they had sample sizes less than 100 patients and did not specify the line of therapy or type of CDK4/6i assessed.

CDK4/6i = cyclin-dependent kinase 4/6 inhibitors; MA = meta-analysis; NCT = National Clinical Trial; PRISMA = Preferred Reporting Items for Systematic Reviews and Meta-Analyses; SLR = systematic literature review.

APPENDIX E: List of included studies

| **Study name; reference** | **Author** | **Year** | **Title** |
| --- | --- | --- | --- |
| 1668 | Amaro | 2021 | First-line treatment with a cyclin-dependent kinase 4/6 inhibitor plus an aromatase inhibitor for metastatic breast cancer in Alberta |
| ESMO23-017 | Bjerrum | 2023 | 391P - Detrimental effect on overall survival of CDK4/6 inhibitor dose reduction if immortal time bias is considered |
| BRAVE;  SABCS23-114 | Werutsky | 2023 | Real-world data on first-line treatment of hormone receptor-positive, HER2-negative, metastatic breast cancer in brazil (BRAVE study - LACOG 0221) |
| 659 | Bruno | 2022 | Cyclin-dependent kinase 4/6 inhibitor outcomes in patients with advanced breast cancer carrying germline pathogenic variants in DNA repair-related genes |
| 4132 | Buller | 2023 | CDK4/6 inhibitors in metastatic breast cancer, a comparison of toxicity and efficacy across agents in a real-world dataset |
| CAPACITY;  ASCO23-003 | Yue | 2023 | Comparative effectiveness of palbociclib plus aromatase inhibitor versus fulvestrant alone as initial endocrine therapy for HR+/HER2- advanced breast cancer in Chinese clinical practice: A real-world study. |
| 638 | Carlino | 2022 | HER2-low status does not affect survival outcomes of patients with metastatic breast cancer (MBC) undergoing first-line treatment with endocrine therapy plus palbociclib: Results of a multicenter, retrospective cohort study |
| CDK-PREDICT;  3381 | Tolosa | 2023 | Efficacy analysis of CDK4/6 inhibitors in combination with endocrine therapy treatment in HR+/HER2- breast cancer according to PAM50 intrinsic subtype: primary results of SOLTI-1801-CDK-PREDICT study |
| 4112 | Cejuela | 2023 | Abemaciclib, palbociclib, and ribociclib in real-world data: A direct comparison of first-line treatment for endocrine-receptor-positive metastatic breast cancer |
| 621 | Choong | 2022 | Clinical management of metastatic hormone receptor-positive, HER2-negative breast cancer (MBC) after CDK 4/6 inhibitors: A retrospective single-institution study |
| 1894 | Collins | 2021 | A real-world evidence study of CDK4/6 inhibitor treatment patterns and outcomes in metastatic breast cancer by germline BRCA mutation status |
| 4060 | Criado | 2023 | Concomitant administration of palbociclib and proton pump inhibitors affects clinical outcomes in metastatic breast cancer patients |
| 576 | Del Re | 2022 | Concomitant administration of proton pump inhibitors does not significantly affect clinical outcomes in metastatic breast cancer patients treated with ribociclib |
| DeMichele 2021;  661 | Brufsky | 2022 | Real-world effectiveness of palbociclib plus letrozole vs letrozole alone for metastatic breast cancer with lung or liver metastases: Flatiron database analysis |
| DeMichele 2021;  1265 | DeMichele | 2021 | Comparative effectiveness of first-line palbociclib plus letrozole versus letrozole alone for HR+/HER2− metastatic breast cancer in US real-world clinical practice |
| DeMichele 2021;  1905 | Brufsky | 2021 | Real-world tumor response of palbociclib plus letrozole versus letrozole for metastatic breast cancer in US clinical practice |
| 563 | Douganiotis | 2022 | Prognostic significance of low HER2 expression in patients with metastatic hormone receptor-positive breast cancer treated with first line cdk4/6 inhibitors: A Greek multicenter real-world data analysis |
| 1809 | El Badri | 2021 | Palbociclib in combination with aromatase inhibitors in patients >= 75 years with oestrogen receptor-positive, human epidermal growth factor receptor 2 negative advanced breast cancer: A real-world multicentre UK study |
| 4009 | Elnaghi | 2023 | Hormonal-receptors-positive and HER2-negative patients with metastatic breast cancer treated with first-line palbociclib and hormonal therapy: Impact of first-cycle neutropenia and dose reduction on therapeutic outcome |
| 556 | Endo | 2022 | Time to chemotherapy for patients with estrogen receptor-positive breast cancer and cyclin-dependent kinase 4 and 6 inhibitor use |
| 606 | Fountzilas | 2020 | Real-world clinical outcome and toxicity data and economic aspects in patients with advanced breast cancer treated with cyclin-dependent kinase 4/6 (CDK4/6) inhibitors combined with endocrine therapy: The experience of the Hellenic Cooperative Oncology Group. |
| 3961 | Gad | 2023 | Clinical outcome and toxicity profile of cyclin-dependent kinase 4/6 inhibitors in combination with hormonal treatment in management of metastatic breast cancer patients: A Middle-East real world experience |
| 3954 | Garly | 2023 | A retrospective, non-interventional study of breast cancer patients diagnosed with ER+/HER2 negative, locally advanced or metastatic breast cancer treated with palbociclib in Denmark |
| GOIRC-04-2019;  SABCS23-009 | Moscetti | 2023 | CDK4/6 inhibitors in advanced breast cancer (aBC). Preliminary results of the CDK4/6i choice and sequence of treatments in a series of 174 patients. GOIRC-04-2019 retro/prospective observational study |
| GOIRC-04-2019;  442 | Izano | 2022 | Time to treatment discontinuation and time to next treatment as proxies of real-world progression-free survival in breast cancer patients |
| 3924 | Goyal | 2023 | Overall survival associated with CDK4/6 inhibitors in patients with HR+/HER2- metastatic breast cancer in the United States: A SEER-Medicare population-based study |
| ESMO23-022 | Gullick | 2023 | 422P - UK real-world data (RWD) of cyclin-dependent kinase 4/6 inhibitor (CDK4/6i) use in metastatic breast cancer (MBC) |
| 3898 | Hall | 2023 | Socioeconomic outcomes with ribociclib in patients with HR+, HER2- advanced breast cancer (ABC) in UK real-world settings |
| SABCS22-067 | Huang | 2022 | High incidence of palbociclib related neutropenia in Asian patients associated with genetic polymorphisms |
| IRIS Europe II;  1901 | Mycock | 2021 | Real-world treatment of patients with palbociclib for HR+/HER2-advanced/metastatic breast cancer: The Europe IRIS study |
| IRIS: Europe, North and South America, and ASIA;  261 | Mycock | 2022 | Real-world treatment patterns and clinical outcomes associated with palbociclib combination therapy: a multinational, pooled analysis from the Ibrance Real World Insights Study |
| KBCSG‑TR‑1316;  3963 | Futamura | 2023 | Detection of high-risk patients resistant to CDK4/6 inhibitors with hormone receptor-positive HER2-negative advanced and metastatic breast cancer in Japan (KBCSG-TR-1316) |
| 2009 | Kim | 2021 | Which clinicopathologic parameters suggest primary resistance to palbociclib in combination with letrozole as the first-line treatment for hormone receptor-positive, HER2-negative advanced breast cancer? |
| 3775 | Kim | 2023 | Efficacy of limited dose modifications for palbociclib-related grade 3 neutropenia in hormone receptor-positive metastatic breast cancer |
| 3779 | Kim | 2023 | Real world experience of second-line treatment strategies after palbociclib and letrozole: Overall survival in metastatic hormone receptor-positive human epidermal growth factor receptor 2-negative breast cancer |
| ESMO23-092 | Kimmick | 2023 | 457P - Disparities in receipt of CDK4/6 inhibitors with endocrine therapy as therapy for hormone receptor-positive, HER2-negative metastatic breast cancer in the real-world setting |
| 3774 | King | 2023 | Abemaciclib for treating patients with HR+, HER2- advanced/metastatic breast cancer in the UK: A real-world study |
| 549 | KovaC | 2020 | Efficacy and safety of selective cyclin-dependent kinases 4/6 inhibitors in hormone-receptor-positive, HER2-negative advanced breast cancer - results from a real-world setting. |
| 951 | Kristensen | 2021 | Dose modifications of ribociclib and endocrine therapy for treatment of ER+ HER2− metastatic breast cancer |
| 3760 | Kruse | 2023 | Treatment patterns and outcomes associated with sequential and non-sequential use of CDK4 & 6 inhibitors in patients with HR+, HER2- MBC in the real world |
| 4679 | Lai | 2023 | Clinical and genotypic insights into higher prevalence of palbociclib associated neutropenia in Asian patients |
| 366 | Law | 2022 | Real-world treatment patterns and clinical effectiveness of palbociclib plus an aromatase inhibitor as first-line therapy in advanced/metastatic breast cancer: Analysis from the US Syapse Learning Health Network |
| ESMO23-066 | Lenza | 2023 | 467P - Initial results from the Canarian registry of luminal breast cancer patients treated with first-line CDK 4/6 inhibitors |
| 324 | Low | 2022 | Real-world outcomes from use of CDK4/6 inhibitors in the management of advanced/metastatic breast cancer in Asia |
| SABCS23-083 | Mardani | 2023 | Clinical outcomes of CDK4/6 inhibitors in patients with bone only metastatic breast cancer |
| SABCS23-045 | Masuda | 2023 | Real-world progression-free survival and overall survival of palbociclib plus endocrine therapy in Japanese patients with HR+/HER2- ABC in the first- or second-line setting: A multicenter observational study |
| 4638 | Matos | 2023 | Efficacy and safety of abemaciclib in the treatment of HR+ HER2-advanced breast cancer: Real world data |
| 285 | Menichetti | 2022 | CDK 4/6 inhibitors for metastatic breast cancer: A multicenter realworld study |
| 284 | Merola | 2022 | Effectiveness research in oncology with electronic health record data: a retrospective cohort study emulating the PALOMA-2 trial |
| 271 | Mouabbi | 2022 | Histology-based survival outcomes in hormone receptor-positive metastatic breast cancer treated with targeted therapies |
| 3627 | Muller | 2023 | CDK4/6 inhibitors in advanced HR+/HER2-breast cancer: A multicenter real-world data analysis |
| NCT04394247;  G-TR23-070 | CT.gov | 2023 | Patient characteristics, treatment patterns, and clinical outcomes in patients diagnosed with HR+/​HER2 advanced/​metastatic breast cancer on palbociclib + aromatase inhibitor (AI) combination therapy |
| NCT04460911;  G-TR24-061 | CT.gov | 2024 | Treatment patterns and clinical outcomes among patients with HR+/HER2- mBC receiving palbociclib combination therapy in the US community oncology setting. |
| SABCS23-084 | Nguyen | 2023 | Efficacy and safety of first line CDK4/6i plus endocrine therapy for patients with HR+/HER2- metastatic breast cancer: initial real-world experience at Ho Chi Minh city oncology hospital, Viet Nam |
| 3597 | Nozawa | 2023 | Real-world treatment patterns and outcomes of abemaciclib for the treatment of HR + , HER2- metastatic breast cancer patients in Japan |
| 3587 | Oikonomidou | 2023 | Real-world clinical outcomes associated with first-line palbociclib and aromatase inhibitor therapy among patients with HR+/HER2- advanced breast cancer in Europe |
| OPAL;  SABCS23-007 | Thill | 2023 | Palbociclib versus ribociclib in first-line treatment of patients with hormone-receptor positive HER2 negative advanced breast cancer – real world outcome data from the German registry platform OPAL |
| PALBOSPAIN;  3682 | Martinez-Janez | 2023 | Palbospain: Observational analysis of first-line therapy with palbociclib in patients with HR+/HER2- metastatic breast cancer (MBC) in real-life conditions |
| PALBOSPAIN;  4203 | Anton | 2023 | Real-world treatment patterns and outcomes of patients receiving palbociclib plus endocrine therapy in Spain: Subgroup analysis based on age, sites and number of metastatic locations, menopausal status and dose received from PALBOSPAIN study |
| PALBOSPAIN;  SABCS23-017 | Moreno | 2023 | Real-world outcomes of patients receiving first-line palbociclib plus endocrine therapy in Spain: Subgroup analysis based on tumor grade, progesterone receptor, Ki-67 and histological subtype from PALBOSPAIN study |
| PALMERES;  4424 | Zattarin | 2023 | Peripheral blood lymphocytes predict clinical outcomes in hormone receptor-positive HER2-negative advanced breast cancer patients treated with CDK4/6 inhibitors |
| 1067 | Palumbo | 2021 | Patterns of treatment and outcome of palbociclib plus endocrine therapy in hormone receptor-positive/HER2 receptor negative metastatic breast cancer: a real-world multicentre Italian study |
| PERFORM;  ESMO23-012 | Radosa | 2023 | 407P - Palbociclib plus endocrine therapy in HR+/HER2- advanced breast cancer patients: Interim results of the PERFORM study |
| POLARIS;  ESMO23-005 | Tripathy | 2023 | 373P - Impact of comorbidities on real-world (rw) clinical outcomes of patients (pts) with hormone receptor-positive/human epidermal growth factor 2-negative (HR+/HER2-) advanced breast cancer (ABC) treated with palbociclib and enrolled in POLARIS |
| P-REALITY-X;  176 | Rugo | 2022 | Real-world study of overall survival with palbociclib plus aromatase inhibitor in HR+/HER2− metastatic breast cancer |
| P-REALITY-X;  177 | Rugo | 2022 | Overall survival with first-line palbociclib plus an aromatase inhibitor (AI) vs AI in metastatic breast cancer: A large real-world database analysis |
| P-REALITY-X;  4379 | Brufsky | 2023 | Real-world treatment patterns and effectiveness of palbociclib plus an aromatase inhibitor in patients with metastatic breast cancer aged 75 years or older |
| P-REALITY-X  4881 | Brufsky | 2023 | Palbociclib combined with an aromatase inhibitor in patients with breast cancer with lung or liver metastases in US clinical practice |
| P-REALITY-X;  SABCS22-020 | Rugo | 2022 | Real-world treatment patterns of palbociclib plus an aromatase inhibitor or aromatase inhibitor alone for metastatic breast cancer in the Flatiron database |
| P-REALITY-X;  SABCS23-018 | Brufsky | 2023 | Real-world effectiveness of palbociclib plus aromatase inhibitors (AI) in metastatic breast cancer patients with cardiovascular diseases |
| 3527 | Queiroz | 2023 | Real-world treatment outcomes in HR+ HER2- metastatic breast cancer patients treated with CDK4/6 inhibitors: Results from a reference center in Brazil |
| 1623 | Rath | 2021 | Efficacy and safety of palbociclib and ribociclib in patients with estrogen and/or progesterone receptor positive, HER2 receptor negative metastatic breast cancer in routine clinical practice |
| REACHAUT;  133 | Singer | 2022 | REACH AUT: Efficacy and safety of first-line (1L) ribociclib (RIB) + endocrine therapy (ET) in HR+, HER2- metastatic breast cancer (MBC) from a real-world (RW) study: 3rd interim analysis |
| REACHAUT;  SABCS23-117 | Singer | 2023 | Real-world outcomes with first-line ribociclib + endocrine therapy in patients with metastatic HR+, HER2– breast cancer: Fourth interim analysis of REACH AUT trial |
| RIBANNA;  ESMO23-044 | Woeckel | 2023 | 441P - Real-world efficacy of ribociclib (RIB) + aromatase inhibitor (AI)/fulvestrant (FUL) in subgroups of special interest: 5th interim analysis (IA) of the RIBANNA study |
| RIBANNA;  SABCS22-089 | Jackisch | 2022 | RIBANNA 5th interim analysis: Matched-pair analysis of progression-free survival (PFS) across treatment cohorts and comparison of frontline ribociclib + endocrine therapy PFS data from RIBANNA vs MONALEESA trials, in HR+, HER2- ABC |
| RIBANNA;  SABCS22-090 | Fasching | 2022 | Progression-free survival and patient-reported outcomes in HR+, HER2- AC patients treated with first-line ribociclib + endocrine therapy (ET) or ET monotherapy or chemotherapy in real-world setting: 5th interim analysis of RIBANNA |
| ROIS;  3569 | Palmieri | 2023 | A real-world study of the first use of palbociclib for the treatment of advanced breast cancer within the UK National Health Service as part of the novel Ibrance Patient Program |
| 3506 | Rottier | 2023 | Pretreatment neutrophil to lymphocyte ratio as prognostic factor in metastatic breast cancer treated with cyclin dependent kinase 4/6 inhibitors |
| 4549 | Rugo | 2023 | Real-world effectiveness of palbociclib plus aromatase inhibitors in African American patients with metastatic breast cancer |
| 3494 | Rugo | 2023 | Real-world comparative effectiveness of palbociclib plus letrozole versus letrozole in older patients with metastatic breast cancer |
| SABCS23-028 | Sánchez | 2023 | Palbociclib in advanced hormone receptor positive breast cancer: A real world study in South America |
| 1198 | Schreier | 2022 | Racial disparities in neutrophil counts among patients with metastatic breast cancer during treatment with CDK4/6 inhibitors |
| SABCS23-105 | Sharaf | 2023 | From clinical trials to clinical practice; real world clinical outcomes of patients treated with ribociclib in combination with aromatase inhibitors or fulvestrant for HRpositive, HER2- negative metastatic breast cancer |
| ASCO23-043 | Solís | 2023 | HER2-low expression as a prognostic factor in patients treated with CDK4/6 inhibitors: A retrospective analysis. |
| 3400 | Tang | 2023 | Palbociclib or ribociclib with aromatase inhibitor in post-menopausal women with ER+/HER2- advanced breast cancer? Real-world overall survival evidence |
| 4506 | Tang | 2023 | Clinical Impact of CDK4/6 Inhibitors in *de novo* or PR- or very elderly post-menopausal ER+/HER2- advanced breast cancers |
| 64 | Visani | 2022 | Safety of CDK4/6 inhibitors and concomitant radiation therapy in patients affected by metastatic breast cancer |
| 4472 | Walbaum | 2023 | Palbociclib in advanced stage hormone receptor-positive breast cancer: real-world data from a Chilean multicentre registry |
| 4381 | Wang, | 2024 | Efficacy, safety, and predictive model of palbociclib in the treatment of HR-positive and HER2-negative metastatic breast cancer |
| SABCS23-065 | Weipert | 2023 | Real-world (RW) utilization and patient outcomes across three CDK4/6 inhibitors in metastatic breast cancer (mBC) |
| ESMO23-006 | Yildirim | 2023 | 429P - The impact of HER2 status on the efficacy of CDK 4/6 inhibitors: A multicenter study |
| SABCS23-059 | Yue | 2023 | Comparative effectiveness of Palbociclib plus aromatase inhibitor versus fulvestrant alone as initial endocrine therapy for HR+/HER2- advanced breast cancer in Chinese clinical practice: a real-world study |
| 1624 | Zhang | 2021 | The efficacy and safety of palbociclib combined with endocrine therapy in patients with hormone receptor-positive HER2-negative advanced breast cancer: a multi-center retrospective analysis |
| 16 | Zhang | 2022 | Clinical outcomes and clinical/genetic risk factors of palbociclib plus endocrine therapy (ET) for HR+HER2- advanced breast cancer (ABC) patients in Chinese multicenter study of real-world practices. |

APPENDIX F: Quality assessment

Newcastle-Ottawa Scale

ISPOR Questionnaire

ESMO-GROW Checklist

APPENDIX G: Effectiveness of any first-line CDK4/6i regimen in single-arm studies

Progression-free survival

The PFS data for patients receiving any CDK4/6i regimen were reported in 19 single-arm studies (**Figure 1A**). Of these, 10 studies reported results for the broader population (**Supplementary Table 1**), four for subgroups of patients exclusively (**Supplementary Tables 2 and 3**), and the remaining five reported both broader and subgroup population data.

**Broader population**

In eight single-arm studies evaluating any CDK4/6i regimen in the broader population, median PFS ranged from 17.8 (n=55)^2^ to 35.6 months (n=175).^3^ The PFS results were similar in the six studies evaluating regimens that included palbociclib or ribociclib but did not distinguish between either CDK4/6i, with median PFS ranging from 19.0 (n=NR)^4^ to 37.9 months (n=316)^5^ when reached. In contrast, one single-arm study reported a median PFS of 28.2 months for a regimen that included palbociclib or abemaciclib (n=91)^6^ (**Supplementary Table 1**).

**Subgroups**

The PFS results for single-arm studies evaluating any CDK4/6i regimen in prespecified subgroups of interest are summarized in **Supplementary Table 2**. These subgroups included ET response (e.g., *de novo*, endocrine resistant, endocrine sensitive), dose reductions, hormonal status (e.g., HER2 status), and race/ethnicity (**Supplementary Table 2**). The PFS results for studies that assessed other subgroups are detailed in **Supplementary Table 3**.

Overall survival

The OS data for patients receiving any CDK4/6i regimen were reported in 11 single-arm studies (**Figure 1A**). Of these, four reported results for the broader population (**Supplementary Table 1**), three reported results for specific patient subgroups exclusively (**Supplementary Tables 2 and 3**), and the remaining four studies reported data for both the broader and subgroup populations.

**Overall Population**

Median OS was often not reached across single-arm studies reporting OS data for patients receiving any CDK4/6i regimen. When median OS was reported, it showed consistent outcomes across different CDK4/6i combinations. Specifically, median OS was 51.0 months for regimens including any CDK4/6i (n=126),^7^ 50.3 months for regimens including either palbociclib or ribociclib (n=NR),^4^ and 61.7 months for regimens that included either palbociclib or abemaciclib (n=91)^6^ (**Supplementary Table 1**).

**Subgroups**

The OS results for single-arm studies evaluating any CDK4/6i regimen in prespecified subgroups of interest are summarized in **Supplementary Table 2**. The OS results for studies that assessed other subgroups are detailed in **Supplementary Table 3**.

**Supplementary Table 1.** Effectiveness outcomes for any first-line CDK4/6i regimen in single-arm RWE studies.

| **Study name; reference** | **Treatment** | **Subgroup** | **Sample size** | **Starting dose for CDK4/6i, n (%)** | **PFS** | | **OS** | |
| --- | --- | --- | --- | --- | --- | --- | --- | --- |
|  |  |  |  |  | Median (95% CI), months | At latest time point, n (%) | Median (95% CI), months | At latest time point, n (%) |
| Muller 2023;    3627-Muller-2023 | CDK4/6i + AI or fulvestrant ± GnRH agonists | All patients | 278 | NR | 23 (NR) | NR | NR | NR |
| PALMERES;  4424-Zattarin-2023 | CDK4/6i + ET | All patients | 335 | NR | 26.1  (23.4–30.8) | NR | NR | NR |
| Rottier 2023;    3506-Rottier-2023 | CDK4/6i + ET | All patients | 126 | NR | 27 (21–36) | 12 months:  NR (73.8) | 51 (NR) | NR |
| 64-Visani-2022 | CDK4/6i | All patients | 55 | NR | 17.8 (NR) | NR | NR | NR |
| 285-Menichetti-2022 | CDK4/6i + ET | All patients | 273 | NR | 24.9  (20.7–29.1) | NR | NR | NR |
| 324-Low-2022 | Palbociclib or ribociclib + fulvestrant, AI, or tamoxifen | All patients | 122 | NR | 28.17 (NR) | NR | NR | 24 months: NR (72) |
| 549-KovaC-2020 | Palbociclib or Ribociclib | All patients | 74 | NR | NR | NR | Not reached | 60 months: Not reached |
| 563-Douganiotis-2022 | CDK4/6i + AI or fulvestrant or tamoxifen in combination with GnRHa | All patients | 191 | NR | 28.9 (NR) | NR | Not reached | NR |
| 606- Fountzilas-2020 | Palbociclib or ribociclib + ET | All patients | 149 | NR | 18.7 (13.5–not reached) | NR | Not reached | NR |
|  | Palbociclib or ribociclib + AI | All patients | 107 | NR | Not reached | NR | NR | NR |
|  | Palbociclib or ribociclib + fulvestrant | All patients | 42 | NR | 24.2 (8.7–not reached) | NR | NR | NR |
| 621-Choong-2022 | Palbociclib + letrozole, fulvestrant, anastrozole, or exemestane, or Abemaciclib | All patients | 91 | NR | 28.2  (19.6–34.9) | NR | 61.7 (56–not reached) | NR |
| 1623-Rath-2021 | Palbociclib or ribociclib + fulvestrant, letrozole, exemestane, letrozole + leuprolide, or fulvestrant + leuprolide | All patients | 22 | NR | 21.1  (16.3–not reached) | NR | NR | NR |
| 1668-Amaro-2021 | Palbociclib or ribociclib + AI | All patients | 316 | NR | 37.9  (26.7–not reached) | NR | Not reached | 36 months: 215 (68.0) |
| 3760-Kruse-2023 | CDK4/6i combination | Group 2: 1L CDK4/6i to 2L non-CDK4/6i | 94 | NR | 22.8  (17.4-–25.0) | NR | NR | NR |
| 3961-Gad-2023 | Palbociclib or Ribociclib + ET | All patients | NR | NR | 19 (NR) | NR | 50.3 (NR) | NR |
| ASCO23-043- Solís-2023 | CDK4/6i + AI or Fulvestrant | All patients | 175 | NR | 35.61  (25.46–45.76) | NR | NR | NR |
| SABCS23-084- Nguyen-2023 | Palbociclib or Ribociclib + ET | All patients | 134 | NR | Not reached | 18 months:  NR (91.5) | NR | NR |

1L = first-line; 2L = second line; AI = aromatase inhibitor; CDK4/6i = cyclin-dependent kinase 4/6 inhibitors; CI = confidence interval; ET= endocrine therapy; GnRHa = Gonadotropin-Releasing Hormone Agonist NR = not reported; OS = overall survival; PFS = progression-free survival; RWE = real-world evidence.

**Supplementary Table 2.** Effectiveness outcomes for prespecified subgroups of interest in any first-line CDK4/6i regimen in single-arm RWE studies.

| **Study name; reference** | **Treatment** | **Subgroup** | **Sample size** | **Starting dose for CDK4/6i, n (%)** | **PFS** | | | **OS** | | |
| --- | --- | --- | --- | --- | --- | --- | --- | --- | --- | --- |
|  |  |  |  |  | Median (95% CI), months | HR (95% CI); *P* value | At latest time point, n (%) | Median (95% CI), months | HR (95% CI); *P* value | At latest time point, n (%) |
| 285-Menichetti-2022 | CDK4/6i + ET | Endocrine-sensitive | 170 | NR | 35.9  (22.4–39.4) | NR | NR | NR | NR | NR |
|  |  | Endocrine-sensitive with visceral disease | 75 | NR | 18.3 | Endocrine-sensitive with no visceral involvement vs Endocrine-sensitive with visceral disease:  0.38  (0.24-0.61);  P < 0.001 | NR | NR |  | NR |
|  |  | Endocrine-sensitive with no visceral involvement | 95 | NR | 50.1 |  | NR | NR |  | NR |
|  |  | Patients with dose reductions | NR | NR | 35.4 | NR | NR | NR |  | NR |
|  |  | Patients with no dose reductions | NR | NR | 16.9 |  | NR | NR |  | NR |
| 563-Douganiotis-2022 | CDK4/6i + AI or fulvestrant or tamoxifen in combination with GnRHa | HER2 0 | 52 | NR | 40.2 (NR) | NR | NR | NR | NR | NR |
|  |  | HER2 +1 | 90 | NR | 26.2 (NR) |  | NR | NR |  | NR |
|  |  | HER1+2/ISH-negative | 47 | NR | 20.9 (NR) |  | NR | NR |  | NR |
| 606- Fountzilas-2020 | Palbociclib or ribociclib + ET | Hormone-sensitive patients | NR | NR | Not reached | NR | NR | Not reached | NR | NR |
|  |  | Hormone-resistant patients | NR | NR | 18.1 (10.5– not reached) |  | NR | Not reached |  | NR |
|  |  | *De novo* metastatic patients | 42 | NR | Not reached |  | NR | Not reached |  | NR |
|  | Palbociclib or ribociclib + AI | Hormone-sensitive patients | 62 | NR | Not reached |  | NR | NR |  | NR |
|  |  | Hormone-resistant patients | 37 | NR | 18.1 (10.5– not reached) |  | NR | NR |  | NR |
|  | Palbociclib or ribociclib + fulvestrant | Hormone resistant patients | 26 | NR | 18.7 (7.9– not reached) |  | NR | NR |  | NR |
| 1198-Schreier-2022 | CDK4/6i + letrozole, fulvestrant, anastrozole, exemestane, or tamoxifen | Black patients | NR | NR | 32.5  (26.0–not reached) | NR | NR | NR | NR | NR |
|  |  | Black patients with neutropenia | NR | NR | 32.5  (26.0–not reached) |  | NR | NR |  | NR |
|  |  | Black patients with dose reduction | NR | NR | 26.3  (18.6–not reached) |  | NR | NR |  | NR |
|  |  | Non-Black patients | NR | NR | 43.2  (33.4–not reached) |  | NR | NR |  | NR |
|  |  | Non-Black patients with neutropenia | NR | NR | 43.2  (33.4–not reached) |  | NR | NR |  | NR |
|  |  | Non-Black patients with dose reduction | NR | NR | 52.3  (33.9–not reached) |  | NR | NR |  | NR |
| ESMO23-006- Yildirim-2023 | CDK4/6i | HER2-0 | 111 | NR | NR | NR | NR | Not reached | HER2-0 vs HER2-low: 0.87 (NR);  P = 0.818 | NR |
|  |  | HER2-low | 49 | NR | NR |  | NR | 49.03 (NR) |  | NR |
| ESMO23-017- Bjerrum-2023 | CDK4/6i + ET | Dose reduction 12w | 189 | NR | 39.8  (35.5–NE) | NR | NR | NR | Dose reduction 12w vs Full dose 12w:  1.63  (1.11-2.38);  *P =* 0.013  Dose reduction naive vs Full dose naive: 0.53  (0.38-0.73);  *P =* 0.00016 | NR |
|  |  | Dose-reduction naïve | 189 | NR | 53.6  (47.2–NE) |  | NR | NR |  | NR |
|  |  | Full dose 12w | 352 | NR | 55.8  (50.1–NE) |  | NR | NR |  | NR |
|  |  | Full-dose naïve | 352 | NR | 39.7  (33.8–49.6) |  | NR | NR |  | NR |
| ASCO23-043- Solís-2023 | CDK4/6i + AI or Fulvestrant | HER2 0+ | 49 | NR | 21.52  (14.3–28.73) | HER2 0+ vs HER2 1+: NR; P = 0.881  HER2 0+ vs HER2 2+: NR; P = 0.155  HER2 1+ vs HER2 2+: NR; P =0.238 | NR | NR | NR | NR |
|  |  | HER2 1+ | 57 | NR | 31.15  (15.59–46.70) |  | NR | NR |  | NR |
|  |  | HER2 2+ | 69 | NR | 39.49 (NE) |  | NR | NR |  | NR |

AI = aromatase inhibitor; CDK4/6i = cyclin-dependent kinase 4/6 inhibitors; CI = confidence interval; ET= endocrine therapy; GnRH = Gonadotropin hormone-releasing hormone; HER2 = human epidermal growth factor receptor 2; ISH = in situ hybridization; NR = not reported; OS = overall survival; PFS = progression-free survival; RWE = real-world evidence.

**Supplementary Table 3.** Effectiveness outcomes for other subgroups in any first-line CDK4/6i regimen in single-arm RWE studies.

| **Study name; reference** | **Treatment** | **Subgroup** | **Sample size** | **Starting dose for CDK4/6i, n (%)** |  | **PFS** | | **OS** | | | |
| --- | --- | --- | --- | --- | --- | --- | --- | --- | --- | --- | --- |
|  |  |  |  |  | Median (95% CI), months | HR (95% CI); *P* value | At latest time point, n (%) | Median (95% CI), months | HR (95% CI); *P* value | At latest time point, n (%) |  |
| CDK-PREDICT;    3381-Tolosa-2023 | CDK4/6i +ET | Luminal Subtypes | 101 | NR | 26.8  (18.9–43.8) | Luminal Subtypes vs Nonluminal Subtypes: 2.44 (1.17-5.07); NR | NR | NR | NR | NR |  |
|  |  | Nonluminal Subtypes | 12 | NR | 10  (5.8–26.0) |  | NR | NR |  | NR |  |
|  |  | Luminal A | NR | NR | NR  (23.0–NR) | Luminal B vs Luminal A: 1.98 (1.09-3.62); NR  HER-2 enriched vs Luminal A: 2.75 (1.05-7.18); NR  Normal-like vs Luminal A: 19.35 (2.32-160.9); NR  Basal-like vs Luminal A: 5.44 (1.44-20.60); NR | NR | NR |  | NR |  |
|  |  | Luminal B | NR | NR | 19.5  (15.7–27.3) |  | NR | NR |  | NR |  |
|  |  | HER-2 enriched | NR | NR | 10  (4.4–NR) |  | NR | NR |  | NR |  |
|  |  | Normal-like | NR | NR | 12.4  (5.8–NR) |  | NR | NR |  | NR |  |
|  |  | Basal-like | NR | NR | NE (NE) |  | NR | NR |  | NR |  |
| KBCSG‑TR‑1316;    3963-Futamura-2023 | Palbociclib or Abemaciclib + ET | NOLUS(+) | 19 | NR | 9.1 (NR) | NOLUS(+) vs NOLUS(-): 3.03 (1.89-4.88); P < 0.001 | 12 months:  NR (46.3) | 27.3 (NR) | NOLUS(+) vs NOLUS(-): 2.41 (1.13-5.17); P = 0.0234 | 12 months:  NR (88.9) |  |
|  |  | NOLUS(-) | 179 | NR | 28.5 (NR) |  | 12 months:  NR (71.3) | 102.3 (NR) |  | 12 months:  NR (94) |  |
| Rottier 2023;    3506-Rottier-2023 | CDK4/6i + ET | High NLR (≥2.53) | 64 | NR | 21.5 (NR) | Low NLR vs High NLR: 0.50 (0.32-0.79); P = 0.002 | 12 months:  42 (65.6) | 43 (NR) | Low NLR vs High NLR: 0.45 (0.23-0.87); P = 0.015 | NR |  |
|  |  | Low NLR (<2.53) | 62 | NR | 39 (NR) |  | 12 months:  50 (80.7) | 56 (NR) |  | NR |  |
|  |  | Lymphopenia (<1.5 G/L) | 67 | NR | 21 (NR) | Lymphopenia vs Normal ALC: 0.52 (0.30-0.90); P = 0.068 | 12 months: 46 (68.7) | 41 (NR) | Lymphopenia vs Normal ALC: 0.58 (0.30-1.10); P = 0.09 | NR |  |
|  |  | Normal ALC/no lymphopenia (≥1.5 G/L) | 59 | NR | 36 (NR) |  | 12 months: 46 (78) | 51 (NR) |  | NR |  |
|  |  | High PLR (≥174.4) | 68 | NR | 22.6 (NR) | High PLR vs Low PLR: 0.73 (0.47-1.15); P = 0.17 | 12 months:  47 (69.1) | NR | NR | NR |  |
|  |  | Low PLR (<174.4) | 58 | NR | 36 (NR) |  | 12 months:  45 (77.6) | NR |  | NR |  |
|  |  | High LMR (≥3.3) | 60 | NR | 36 (NR) | High LMR vs Low LMR: 0.75 (0.48-1.18); P = 0.21 | 12 months:  43 (71.7) | NR | NR | NR |  |
|  |  | Low LMR (< 3.3) | 66 | NR | 24.5 (NR) |  | 12 months:  49 (74.2) | NR |  | NR |  |
| 1894-Collins-2021 | CDK4/6i combinations | BRCA, mutation | 36 | NR | NR | NR | NR | 26  (22–not reached) | BRCA mutation vs BRCA wild type:1.50 (1.06-2.14); NR | NR |  |
|  |  | BRCA, wild-type | 293 | NR | NR |  | NR | 51 (NR) |  | NR |  |

AI = aromatase inhibitor; ALC = absolute lymphocyte count; BRCA = breast cancer gene; CDK4/6i = cyclin-dependent kinase 4/6 inhibitors; CI = confidence interval; ET= endocrine therapy; HER2 = human epidermal growth factor receptor 2; LMR = lymphocyte-monocyte ratio; NLR = neutrophil-lymphocyte ratio; NOLUS = nonluminal disease score; NR = not reported; OS = overall survival; PLR = platelet-lymphocyte ratio; PFS = progression-free survival; RWE = real-world evidence.

APPENDIX H: Effectiveness outcomes for first-line CDK4/6i in single-arm RWE studies

**Supplementary Table 1.** Effectiveness outcomes for overall first-line palbociclib in single-arm RWE studies.

| **Study name; reference** | **Treatment** | **Subgroup** | **Sample size** | **Starting dose for CDK4/6i, n (%)** | **PFS** | | **OS** | |
| --- | --- | --- | --- | --- | --- | --- | --- | --- |
|  |  |  |  |  | Median (95% CI), months | At latest time point, n (%) | Median (95% CI), months | At latest time point, n (%) |
| GOIRC-04-2019;    442-Izano-2022 | Palbociclib + AI | All patients | 241 | NR | 39 (30–not reached) | NR | NR | NR |
| NCT04394247    G-TR23-070-2023 | Palbociclib + AI | All patients | 242 | 125 mg: NR (89.7)  100 mg: NR (7.4)  75 mg: NR (1.2) | NR | 36 months: NR (48) | NR | 36 months: NR (69) |
| NCT04460911    G-TR24-061-2024 | Palbociclib + fulvestrant | All patients | 317 | 125 mg: NR (92.4) | 19.6  (15.2–23.6) | NR | 44.1 (39.4– not reached) | NR |
| PALBOSPAIN ;    3682- Martinez-Janez-2023 | Palbociclib + ET | All patients | 762 | NR | 24 (25–27) | NR | 42 (40–not reached) | NR |
| PERFORM;    ESMO23-012- Radosa-2023 | Palbociclib + ET | All patients | 624 | NR | Not reached (19.9–not reached) | 18 months: NR (60.8) | NR | NR |
| ROIS;    3569-Palmieri-2023 | Palbociclib + AI or fulvestrant or unclassified | All patients | 137 | NR | 22.8 (16.5–not reached) | 24 months: NR (48.9) | Not reached | 24 months: NR (74.2) |
|  | Palbociclib + letrozole | All patients | 30 | NR | 11.8 (10.0–not reached) | 24 months: NR (36.7) | Not reached | 24 months: NR (70) |
| 16-Zhang-2022 | Palbociclib + ET | All patients | NR | NR | 19 (NR) | NR | NR | NR |
| 366-Law-2022 | Palbociclib + AI | All patients | 242 | 125 mg: 217 (89.7)  100 mg: 18 (7.4)  75 mg: 3 (1.7)  Unknown: 4 (1.2) | 31.7 (27.9– not reached) | NR | NR | 24 months: NR (78) |
| 638-Carlino-2022 | Palbociclib + ET | All patients | 165 | NR | 20  (18–25) | NR | Not reached | 48 months: 97 (59) |
| 2009-Kim-2021 | Palbociclib + letrozole | All patients | 305 | NR | 28.7  (22.5–34.9) | NR | Not reached | NR |
| 3587-  Oikonomidou-2023 | Palbociclib + AI | All patients | 668 | NR | 31.8  (27.7–35.4) | 36 months: NR (45.2) | NR | NR |
| 3775-Kim-2023 | Palbociclib + ET | All patients | 434 | 125 mg: 434 (100) | 37.8  (28.4–NR) | 24 months:  NR (61.1) | NR | 24 months: NR (91.4) |
| 3779-Kim-2023 | Palbociclib + Letrozole | All patients | 305 | NR | 29  (23.5–34.4) | NR | NR | 60 months: NR (66.5) |
| 4009- Elnaghi-2023 | Palbociclib + ET | All patients | 1066 | NR | 22 (NR) | NR | 33 (NR) | NR |
| 4381-Wang-2023 | Palbociclib + ET | All patients | 103 | 125 mg: 103 (100)  100 mg: 0 | 12.13  (5.47–19.62) | NR | NR | NR |
| 4472-Walbaum-2023^a^ | Palbociclib + ET | All patients | 67 | NR | NR | NR | 111 (NR) | 5 years: NR (89) |
| 4679-Lai-2023 | Palbociclib + ET | All patients | 57 | NR | 20.4 (NR) | NR | Not reached | NR |
| SABCS22-067-Huang-2022 | Palbociclib | All patients | 53 | NR | Not reached | NR | Not reached | NR |
| SABCS23-028-C.Sanchez-2023 | Palbociclib + ET | All patients | NR | NR | NR | 12 months: NR (67.2) | NR | NR |
| SABCS23-045-N. Masuda-2023 | Palbociclib + ET | All patients | 420 | 125 mg:  380 (90.5)  100 mg: 32 (7.6)  75 mg: 8 (1.9)  Other: 0 | 24.5  (19.9–29.4) | NR | Not reached (52.7–NE) | NR |

^a^Patients with previous chemotherapy were excluded from this study, which may have removed patients with higher burden of disease or visceral crisis. These patients were relatively young (median age of 49 years) with access to high-cost treatments and supportive care.

AI = aromatase inhibitor; CI = confidence interval; ET= endocrine therapy; NR = not reported; OS = overall survival; PFS = progression-free survival; RWE = real-world evidence.

**Supplementary Table 2.** Effectiveness outcomes for first-line ribociclib in single-arm RWE studies.

| **Study name; reference** | **Treatment** | **Subgroup** | **Sample size** | **Starting dose for CDK4/6i, n (%)** | **PFS** | | **OS** | |
| --- | --- | --- | --- | --- | --- | --- | --- | --- |
|  |  |  |  |  | Median (95% CI), months | At latest time point, n (%) | Median (95% CI), months | At latest time point, n (%) |
| REACHAUT;    133-Singer-2022 | Ribociclib + AI or fulvestrant | All patients | 283 | NR | 29.7 (NR) | NR | NR | 12 months: 256 (90.3) |
| 3898-Hall-2023 | Ribociclib + AI | All patients | 154 | NR | 20.6  (17.5–not estimable) | NR | NR | NR |

AI = aromatase inhibitor; CDK4/6i = cyclin-dependent kinase 4/6 inhibitors; CI = confidence interval; NR = not reported; OS = overall survival; PFS = progression-free survival; RWE = real-world evidence.

**Supplementary Table 3.** Effectiveness outcomes for first-line abemaciclib in single-arm RWE studies.

| **Study name; reference** | **Treatment** | **Subgroup** | **Sample size** | **Starting dose for CDK4/6i, n (%)** | **PFS** | | **OS** | |
| --- | --- | --- | --- | --- | --- | --- | --- | --- |
|  |  |  |  |  | Median (95% CI), months | At latest time point, n (%) | Median (95% CI), months | At latest time point, n (%) |
| 3597-Nozawa-2023 | Abemaciclib + ET | All patients | 63 | NR | 21.4 (NR) | 12 months:  NR (71.6) | NR | NR |
| 3774-King-2023 | Abemaciclib + ET | All patients | NR | NR | NR | 12 months: NR (81.1) | NR | NR |
| 4638-Matos-2023 | Abemaciclib + ET | All patients | 69 | NR | 23  (13.3–32.7) | NR | Not reached | NR |

CDK4/6i = cyclin-dependent kinase 4/6 inhibitors; CI = confidence interval; ET= endocrine therapy; NR = not reported; OS = overall survival; PFS = progression-free survival; RWE = real-world evidence.

APPENDIX I: Effectiveness outcomes for first-line CDK4/6i in subgroup populations

Single-arm RWE Studies

**Supplementary Table 1.** Effectiveness outcomes for prespecified subgroups of interest receiving first-line palbociclib in single-arm RWE studies.

| **Study name; reference** | **Treatment** | **Subgroup** | **Sample size** | **Starting dose for CDK4/6i, n (%)** | **PFS** | | | **OS** | | |
| --- | --- | --- | --- | --- | --- | --- | --- | --- | --- | --- |
|  |  |  |  |  | Median (95% CI), months | HR (95% CI); *P* value | At latest time point, n (%) | Median (95% CI), months | HR (95% CI); *P* value | At latest time point, n (%) |
| PALBOSPAIN ;    3682- Martinez-Janez-2023 | Palbociclib + ET | Cohort A: *de novo* | 233 | NR | 28 (23–39) | NR | NR | NR | NR | NR |
|  |  | Cohort B: relapsed mBC > 12 months end of adjuvant hormone therapy | 220 | NR | 29 (25–35) |  | NR | NR |  | NR |
|  |  | Cohort C: relapsed mBC within 12 months end of adjuvant hormone therapy | 309 | NR | 14 (11–17) |  | NR | NR |  | NR |
| PALBOSPAIN;    4203- Anton-2023 | Palbociclib + ET | <50 years old | 130 | NR | 27 (21–35) | NR | NR | NR | NR | NR |
|  |  | 50–70 years old | 376 | NR | 21 (19–26) |  | NR | NR |  | NR |
|  |  | >70 years old | 219 | NR | 27 (22–37) |  | NR | NR |  | NR |
|  |  | Premenopausal | 114 | NR | 27 (20–35) | NR | NR | NR |  | NR |
|  |  | Postmenopausal | 613 | NR | 24 (21–27) |  | NR | NR |  | NR |
|  |  | Visceral metastases | 418 | NR | 20 (18–23) | NR | NR | NR |  | NR |
|  |  | No visceral metastases | 344 | NR | 30 (25–36) |  | NR | NR |  | NR |
|  |  | ≥1 dose reduction | 377 | NR | 28 (25–33) | ≥1 dose reduction vs no dose reduction: 0.71 (0.58-0.85); P = 0.003 | NR | NR |  | NR |
|  |  | No dose reduction | 385 | NR | 19 (16–23) |  | NR | NR |  | NR |
| PALBOSPAIN;  SABCS23-017-F. Moreno.-2023 | Palbociclib + ET | PR positive | 530 | NR | 27 (23–31) | PR positive vs PR negative: 0.72 (0.59-0.88); P = 0.001 | NR | 44 (40–not reached) | PR positive vs PR negative: 0.73 (0.57-0.94); P = 0.01 | NR |
|  |  | PR negative | 213 | NR | 18 (15–24) |  | NR | 40 (35–not reached) |  | NR |
| POLARIS;      ESMO23-005- Tripathy-2023 | Palbociclib + ET | Vascular disorders | 495 | NR | 23.7  (19.1–28.1) | NR | NR | 50.8 (40.5– not reached) | NR | NR |
|  |  | Psychiatric disorders | 236 | NR | 19.6  (14.6–27.2) |  | NR | 47.1 (35.7– not reached) |  | NR |
|  |  | Metabolic/nutritional disorders | 172 | NR | 19.6  (14.6–26.3) |  | NR | 42.7  (35.6–50.8) |  | NR |
|  |  | Blood/lympatic disorders | 144 | NR | 16.7  (12.2–19.6) |  | NR | 36.5 (28.8– not reached) |  | NR |
|  |  | Cardiac disorders | 32 | NR | 12.5  (8.5–34.1) |  | NR | 34.1 (18.1– not reached) |  | NR |
|  |  | CCI 0 | 274 | NR | 20.3  (17.7–24.8) |  | NR | 48.8 (37.3– not reached) |  | NR |
|  |  | CCI 1-2 | 489 | NR | 24.2  (19.4–29.5) |  | NR | NR (43.0– not reached) |  | NR |
|  |  | CCI ≥3 | 138 | NR | 16.8  (11.2–20.8) |  | NR | 34.8  (29.1-44.1) |  | NR |
| 366-Law-2022 | Palbociclib + AI | Bone only metastases | 123 | NR | 44.9 (39.4– not reached) | NR | NR | NR | NR | NR |
|  |  | Visceral metastases | 78 | NR | 27.9 (13.8– not reached) |  | NR | NR |  | NR |
|  |  | Age <50 at diagnosis | 25 | NR | NR (13.3– not reached) |  | NR | NR |  | NR |
|  |  | Age 50–64 at diagnosis | 86 | NR | 26.5 (17.4– not reached) |  | NR | NR |  | NR |
|  |  | Age 65–74 at diagnosis | 80 | NR | 41.9 (29.8– not reached) |  | NR | NR |  | NR |
|  |  | Age ≥75 at diagnosis | 51 | NR | 35.8 (21.2– not reached) |  | NR | NR |  | NR |
|  |  | ECOG PS 0 | 57 | NR | 29.8 (27.9– not reached) |  | NR | NR |  | NR |
|  |  | ECOG PS 1 | 47 | NR | 31.7 (19.4– not reached) |  | NR | NR |  | NR |
|  |  | ECOG PS 2+ | 29 | NR | 13.8 (5.7– not reached) |  | NR | NR |  | NR |
|  |  | ECOG PS unknown | 98 | NR | 38.8 (30.5– not reached) |  | NR | NR |  | NR |
|  |  | CCI score 0 | 158 | NR | 44.9 (28.0– not reached) |  | NR | NR |  | NR |
|  |  | CCI score 1 | 47 | NR | 26.5 (13.3– not reached) |  | NR | NR |  | NR |
|  |  | CCI score 2+ | 37 | NR | 29.8 (21.2– not reached) |  | NR | NR |  | NR |
|  |  | White | 196 | NR | 35.8 (24.4– not reached) |  | NR | NR |  | NR |
|  |  | Black or African American | 29 | NR | 18.5 (13.8– not reached) |  | NR | NR |  | NR |
|  |  | Initiated palbociclib at 125 mg | 217 | 125 mg: 217 (100) | 31.7 (26.5– not reached) |  | NR | NR |  | NR |
|  |  | Initiated palbociclib at <125 mg | 25 | <125 mg: 25 (100) | 38.8 (10.1– not reached) |  | NR | NR |  | NR |
| 638-Carlino-2022 | Palbociclib + ET | HER2-zero | 94 | NR | 23  (18–27) | HER2-zero vs HER2-low: 0.77 (0.52-1.1); P = 0.18 | NR | Not reached | HER2-zero vs HER2-low: 0.62 (0.32-1.16); P = 0.13 | 48 months: 58 (62) |
|  |  | HER2-low | 71 | NR | 19  (14–21) |  | NR | Not reached |  | 48 months:  36 (51) |
| 1809-El Badri-2021 | Palbociclib + AI | All patients (older adults) | 276 | 125 mg: 243 (88)  100 mg: 29 (10.5) 75 mg: 3 (1.1)  NR: 1 (0.4) | NR | NR | 24 months: 179 (64.9) | NR | NR | 24 months: 204 (74) |
|  |  | PS 0-1 | 218 (79.0) | NR | NR | PS 0-1 vs PS 2-4: 3.13 (1.97-4.97); P < 0.001 | NR | NR | PS 0-1 vs PS 2-4: 3.64 (2.16-6.13); P < 0.001 | NR |
|  |  | PS 2-4 | 54 (19.6) | NR | NR |  | NR | NR |  | NR |
|  |  | Starting dose 125 mg | 243 (88.0) | 125 mg: 243 (88.0) | NR | Starting dose 125 mg vs starting dose < 125 mg: 1.459 (0.69-3.10); P = 0.327 | NR | NR | Starting dose 125 mg vs starting dose < 125 mg: 1.88 (0.72-4.91); P = 0.196 | NR |
|  |  | Starting dose < 125 mg | 32 (11.6) | 100 mg: 29 (10.5)  75 mg: 3 (1.1)  NR: 1 (0.4) | NR |  | NR | NR |  | NR |
|  |  | No dose reduction | NR | NR | NR | No dose reduction vs dose reduction: 0.81 (0.51-1.30); P = 0.391 | NR | NR | No dose reduction vs dose reduction: 0.54 (0.31-0.93); P = 0.327 | NR |
|  |  | Dose reduction | NR | NR | NR |  | NR | NR |  | NR |
| 2009-Kim-2021 | Palbociclib + letrozole | ER strong | 278 | NR | 30.3  (23.9–36.7) | NR | NR | NR | NR | NR |
|  |  | ER weak | 18 | NR | 11.9  (4.7–19.2) |  | NR | NR |  | NR |
|  |  | PR strong | 147 | NR | 31.3  (24.9–37.8) |  | NR | NR |  | NR |
|  |  | PR weak | 74 | NR | 24.5  (13.4–35.6) |  | NR | NR |  | NR |
|  |  | PR negative | 75 | NR | 17.9  (8.8–27.0) |  | NR | NR |  | NR |
|  |  | No ET | 126 | NR | 25.4  (15.3–35.6) | Primary ET resistance vs no ET: 1.91 (1.13-3.24); P = 0.022  Secondary ET resistance vs no ET: 0.87 (0.52-1.49); P = 0.022  ET sensitive vs no ET: 0.81 (0.50-1.32); P = 0.022 | NR | NR |  | NR |
|  |  | Primary resistance | 38 | NR | 12.7  (2.8–22.6) |  | NR | NR |  | NR |
|  |  | Secondary resistance | 58 | NR | 20.3  (26.2–34.4) |  | NR | NR |  | NR |
|  |  | Sensitive | 83 | NR | Not reached |  | NR | NR |  | NR |
|  |  | No visceral disease | 240 | NR | 31.3  (26.1–26.9) | NR | NR | NR |  | NR |
|  |  | Visceral | 65 | NR | 15.3  (3.7–26.9) |  | NR | NR |  | NR |
|  |  | No liver metastases | 245 | NR | 31.3  (26.1–36.6) | Liver metastasis vs no liver metastasis: 2.17 (1.42-3.31); *P* < 0.001 | NR | NR |  | NR |
|  |  | Liver metastases | 60 | NR | 12.7  (0.6–24.8) |  | NR | NR |  | NR |
|  |  | Bone only metastases | 90 | NR | 29  (20.2–37.8) | NR | NR | NR |  | NR |
|  |  | Other metastases | 114 | NR | 28.7  (20.9–36.5) |  | NR | NR |  | NR |
|  |  | Stage IV lymph node only | 36 | NR | Not reached |  | NR | NR |  | NR |
|  |  | Risk factor 0 | 106 | NR | Not reached | NR | NR | NR |  | NR |
|  |  | Risk factor 1 | 102 | NR | 28  (21.6–34.4) |  | NR | NR |  | NR |
|  |  | Risk factor 2 | 41 | NR | 8.2  (2.1–14.2) |  | NR | NR |  | NR |
|  |  | Risk factor 3 | 7 | NR | 6.8  (4.2–9.4) |  | NR | NR |  | NR |
| 3775-Kim-2023 | Palbociclib + ET | Group 1 (palbociclib dose was maintained with afebrile grade 3 neutropenia within cycle 5) | 174 | NR | NR | Group 2 vs Group 1: 0.67 (0.45-0.99); P = 0.044 | 24 months:  NR (67.9) | NR | NR | 24 months: NR (95.4) |
|  |  | Group 2 (patients who experienced dose modification with afebrile grade 3 neutropenia within cycle 5) | 128 | NR | NR |  | 24 months: NR (55.3) | NR |  | 24 months: NR (90.1) |
| 4009-Elnaghi-2023 | Palbociclib + ET | Dose reduction | 70 | NR | 22 (NR) | No dose reduction vs dose reduction: 1.33 (0.83-2.14); P = 0.23 | NR | NR | No dose reduction vs dose reduction: 1.96 (1.032-3.73); P = 0.04 | NR |
|  |  | No dose reduction | 80 | NR | 25 (NR) |  | NR | 24(NR) |  | NR |
|  |  | HER2-low | 67 | NR | 25 (NR) | HER2-low vs HER2-Zero: 1.03 (0.64-1.64); P = 0.92 | NR | 39(NR) | HER2-low vs HER2-Zero: 1.01 (0.54-1.89); P = 0.97 | NR |
|  |  | HER2-Zero | 83 | NR | 13 (NR) |  | NR | 22(NR) |  | NR |
|  |  | Premenopausal | 78 | NR | 25 (NR) | Premenopausal vs Postmenopausal: 1.11 (0.69-1.77); P = 0.67 | NR | NR | Premenopausal vs Postmenopausal: 1.17 (0.62-2.17); P = 0.963 | NR |
|  |  | Postmenopausal | 72 | NR | 15 (NR) |  | NR | 28(NR) |  | NR |
|  |  | High Allred score | 126 | NR | 35 (NR) | Low Allred score vs High Allred score: 2.42 (1.39-4.23); P = 0.002 | NR | NR | Low Allred score vs High Allred score: 2.81 (1.44-5.46); P = 0.002 | NR |
|  |  | Low Allred score | 24 | NR | 20 (NR) |  | NR | 18(NR) |  | NR |
|  |  | PR positive | 127 | NR | 38 (NR) | PR positive vs PR negative: 1.96 (1.07-3.62); *P* = 0.03 | NR | 39(NR) | PR negative vs PR positive:  2.31 (1.09-4.91); *P* = 0.029 | NR |
|  |  | PR negative | 23 | NR | 18 (NR) |  | NR | 28(NR) |  | NR |
|  |  | Bone-only | 32 | NR | 26 (NR) | Bone-only vs Others score:  0.45 (0.23-0.88); *P* = 0.02 | NR | 33(NR) | Bone-only vs Others score:  0.29 (0.11-0.85); *P* = 0.024 | NR |
|  |  | Others | 118 | NR | 19 (NR) |  | NR | 39(NR) |  | NR |
|  |  | PS 2 | 39 | NR | 22 (NR) | PS2 vs PS 0-1:  1.097 (0.65-1.85); *P* = 0.73 | NR | 40(NR) | PS2 vs PS 0-1:  0.83 (0.53-1.62); *P* = 0.59 | NR |
|  |  | PS 0–1 | 111 | NR | 24 (NR) |  | NR | NR |  | NR |
| SABCS23-045-N. Masuda-2023 | Palbociclib + ET | *De novo* | 109 | NR | 33.6  (27.0–42.4) | NR | NR | Not reached (52.7–NE) | NR | NR |
|  |  | TFI* ≥12 months | 86 | NR | 27.3  (20.4–NE) |  | NR | Not reached (NE–NE) |  | NR |
|  |  | TFI* <12 months | 193 | NR | 14.5  (12.1–19.2) |  | NR | 50.1  (41.5–NE) |  | NR |
|  |  | Nonvisceral | 212 | NR | 27.8  (21.7–35.5) |  | NR | Not reached (51.0–NE) |  | NR |
|  |  | Visceral | 208 | NR | 21.3  (16.1–27.0) |  | NR | Not reached  (47.2–NE) |  | NR |
| SABCS23-083- M. Mardani-2023 | Palbociclib +ET | Bone only | 30 | NR | 20  (12.91–27.08) | NR;  P = 0.052 | NR | NR | NR | NR |
|  |  | Visceral ± bone | 45 | NR | 28.4  (16.38–40.41) |  | NR | NR |  | NR |

* Treatment-free interval (TFI) was defined as the time from the end of adjuvant therapy to the diagnosis date of recurrence.

AI = aromatase inhibitor; CCI = Charleson comorbidity index; CDK4/6i = cyclin-dependent kinase 4/6 inhibitors; CI = confidence interval; ECOG = Eastern Cooperative Oncology Group; ER = estrogen receptor; ET= endocrine therapy; HER2 = human epidermal growth factor receptor 2; mBC = breast cancer; NR = not reported; OS = overall survival; PFS = progression-free survival; PR = progesterone receptor; PS = performance score; RWE = real-world evidence; TFI = treatment-free interval.

**Supplementary Table 2.** Effectiveness outcomes for other subgroups receiving first-line palbociclib in single-arm RWE studies.

| **Study name; reference** | **Treatment** | **Subgroup** | **Sample size** | **Starting dose for CDK4/6i, n (%)** | **PFS** | | | **OS** | | |
| --- | --- | --- | --- | --- | --- | --- | --- | --- | --- | --- |
|  |  |  |  |  | Median (95% CI), months | HR (95% CI);  *P* -value | At latest time point, n (%) | Median (95% CI), months | HR (95% CI);  *P* -value | At latest time point, n (%) |
| PALBOSPAIN;    4203- Anton-2023 | Palbociclib + ET | 1 metastatic site | 325 | NR | 34 (28–39) | NR | NR | NR | NR | NR |
|  |  | 2-3 metastatic sites | 328 | NR | 20 (18–24) |  | NR | NR |  | NR |
|  |  | >3 metastatic sites | 109 | NR | 19 (14–24) |  | NR | NR |  | NR |
| PALBOSPAIN;  SABCS23-017-F. moreno.-2023 | Palbociclib + ET | Grade 1 | 79 | NR | 33 (43–not reached) | Grade 1 vs Grade 3: 0.65 (0.44-NR); NR  Grade 2 vs Grade 3: 0.87 (0.68-1.12); P = 0.30 | NR | 43 (43–not reached) | Grade 1 vs Grade 3: 0.50 (0.30-0.85); P = 0.01  Grade 2 vs Grade 3: 0.71 (0.52-0.97); P = 0.03 | NR |
|  |  | Grade 2 | 363 | NR | 24 (42–not reached) |  | NR | 42 (42–not reached) |  | NR |
|  |  | Grade 3 | 132 | NR | 19 (40–not reached) |  | NR | 40 (40–not reached) |  | NR |
|  |  | Ductal histotype | 575 | NR | 23 (20–27) | Ductal histotype vs Lobular histotype: 0.96 (0.74-1.25); P = 0.7 | NR | 42 (40–not reached) | Ductal histotype vs Lobular histotype: 0.94 (0.68-1.31); P = 0.7 | NR |
|  |  | Lobular histotype | 110 | NR | 25 (19–36) |  | NR | 43 (35–not reached) |  | NR |
|  |  | Ki-67 <20 | 255 | NR | 35 (30–not reached) | Ki-67 <20 vs Ki-67 ≥20: 0.60 (0.48-0.75); P = 0.0004 | NR | Not reached (43–not reached) | Ki-67 <20 vs Ki-67 ≥20: 0.66 (0.50-0.86); P = 0.002 | NR |
|  |  | Ki-67 ≥20 | 398 | NR | 20 (18–23) |  | NR | 38 (36–not reached) |  | NR |
| 366-Law-2022 | Palbociclib + AI | Patients with stage IV disease at diagnosis | 134 | NR | 38.8 (24.42– not reached) | NR | NR | NR | NR | NR |
|  |  | Advanced or metastatic | 137 | NR | 38.8 (26.5– not reached) |  | NR | NR |  | NR |
|  |  | Unknown stage at diagnosis | 16 | NR | 29.8 (23.9– not reached) |  | NR | NR |  | NR |
|  |  | 1 metastatic site | 147 | NR | 44.9 (29.6– not reached) |  | NR | NR |  | NR |
|  |  | 2 metastatic sites | 45 | NR | 31.6 (14.0– not reached) |  | NR | NR |  | NR |
|  |  | ≥3 metastatic sites | 49 | NR | 14.7 (12.3– not reached) |  | NR | NR |  | NR |
| 2009-Kim-2021 | Palbociclib + letrozole | Ki-67 1+ | 167 | NR | 31.3  (25.6–37.1) | Ki-67 2+ vs 1+: 1.42 (0.94-2.14); P < 0.001  Ki-67 3+ vs 1+: 2.58 (1.17-5.67); P < 0.001  Ki-67 4+ vs 1+: 10.28 (3.52-30.09); P < 0.001 | NR | NR | NR | NR |
|  |  | Ki-67 2+ | 83 | NR | 20.9  (8.3–33.6) |  | NR | NR |  | NR |
|  |  | Ki-67 3+ | 14 | NR | 12.2  (9.0–15.5) |  | NR | NR |  | NR |
|  |  | Ki-67 4+ | 4 | NR | 5.1  (0.0–14.1) |  | NR | NR |  | NR |
|  |  | BRCA, wild-type | 300 | NR | 28.7  (21.9–35.5) | BRCA wildtype vs BRCA mutation: 9.59 (3.58-25.70); P < 0.001 | NR | NR |  | NR |
|  |  | BRCA, mutation | 5 | NR | 5.7  (0.0–14.0) |  | NR | NR |  | NR |
|  |  | 1 metastatic site | 158 | NR | 33.3 (NR) | 2 metastatic sites vs 1 metastatic site: 1.29 (0.83-2.01); P = 0.368  3 or more metastatic sites vs 1 metastatic site: 1.42 (0.81-2.50); P = 0.368 | NR | NR |  | NR |
|  |  | 2 metastatic sites | 100 | NR | 23.2  (15.4–31.0) |  | NR | NR |  | NR |
|  |  | 3 metastatic sites | 47 | NR | 15.3  (3.0–28.7) |  | NR | NR |  | NR |
|  |  | Normal baseline CA-15-3 level | 175 | NR | 35  (28.9–41.1) | Elevated baseline CA-15-3 level vs normal baseline CA-15-3 level: 1.99 (1.31-3.01); P = 0.005  Baseline CA-15-3 level not evaluated vs normal baseline CA-15-3 level: 1.93 (0.68-5.45); P = 0.005 | NR | NR |  | NR |
|  |  | Elevated baseline CA-15-3 level | 111 | NR | 17.7  (11.3–24.1) |  | NR | NR |  | NR |
|  |  | Baseline CA-15-3 level not evaluated | 19 | NR | 21.5  (12.3–30.7) |  | NR | NR |  | NR |
| 4009- Elnaghi-2023 | Palbociclib + ET | Neutropenia | 129 | NR | 25 (NR) | Neutropenia vs no neutropenia: 1.13  (0.54-2.36);  *P* = 0.75 | NR | 38(NR) | No neutropenia vs neutropenia: 0.93  (0.533-2.61); *P* = 0.89 | NR |
|  |  | No neutropenia | 21 | NR | 22 (NR) |  | NR | 39(NR) |  | NR |
|  |  | Grade 3/4 neutropenia | NR | NR | 24 (NR) | Grade 3/4 neutropenia vs Grade 1/2 neutropenia: 0.76  (0.41-1.41);  *P* = 0.39 | NR | 39(NR) | Grade 3/4 neutropenia vs Grade 1/2 neutropenia: 0.76  (0.41-1.41);  *P* = 0.39 | NR |
|  |  | Grade1/2 neutropenia | NR | NR | 22 (NR) |  | NR | 40(NR) |  | NR |
|  |  | Single site | 54 | NR | 21 (NR) | Single site vs Multiple site: 0.34  (0.19-0.59);  *P* < 0.00001 | NR | 33(NR) | Single site vs Multiple site: 0.25  (0.11-0.60);  *P* < 0.00001 | NR |
|  |  | Multiple site | 96 | NR | 23 (NR) |  | NR | 38(NR) |  | NR |
|  |  | Ki-67 low | 60 | NR | 22 (NR) | Ki-67 low vs Ki-67 high: 0.63  (0.39-1.02);  *P* = 0.058 | NR | 39(NR) | Ki-67 low vs Ki-67 high: 0.55  (0.29-1.036); *P* = 0.064 | NR |
|  |  | Ki-67 high | 86 | NR | 25 (NR) |  | NR | 39(NR) |  | NR |
| 4679-Lai-2023 | Palbociclib + ET | Earlier neutropenia | 29 | NR | 23.4 (NR) | NR | NR | NR | NR | NR |
|  |  | Later neutropenia | 26 | NR | 33.6 (NR) |  | NR | NR |  | NR |

AI = aromatase inhibitor; BRCA = breast cancer gene; CDK4/6i = cyclin-dependent kinase 4/6 inhibitors; CI = confidence interval; ET= endocrine therapy; NR = not reported; OS = overall survival; PFS = progression-free survival; RWE = real-world evidence.

**Supplementary Table 3.** Effectiveness outcomes for prespecified subgroups of interest receiving first-line ribociclib in single-arm RWE studies.

| **Study name; reference** | **Treatment** | **Subgroup** | **Sample size** | **Starting dose for CDK4/6i, n (%)** | **PFS** | | | **OS** | | |
| --- | --- | --- | --- | --- | --- | --- | --- | --- | --- | --- |
|  |  |  |  |  | Median (95% CI), months | HR (95% CI);  *P* value | At latest time point, n (%) | Median (95% CI), months | HR (95% CI);  *P* value | At latest time point, n (%) |
| REACHAUT;    133-Singer-2022 | Ribociclib + AI or fulvestrant | Patients with visceral metastasis | 116 | NR | 32.7 (NR) | NR | NR | NR | NR | NR |
| 951-Kristensen-2021 | Ribociclib + AI or fulvestrant | Patients without any dose reduction | 46 | NR | 15.6 (10.4–not reached) | NR | NR | NR | NR | NR |
|  |  | Patients with late dose reduction | 31 | NR | Not reached (19.2–not reached) |  | NR | NR |  | NR |

AI = aromatase inhibitor; CDK4/6i = cyclin-dependent kinase 4/6 inhibitors; CI = confidence interval; NR = not reported; OS = overall survival; PFS = progression-free survival; RWE = real-world evidence.

Comparative RWE Studies: CDK4/6i versus ET

**Supplementary Table 4.** Effectiveness outcomes for prespecified subgroups of interest receiving first-line palbociclib in comparative RWE studies versus ET.

| **Study name; reference** | **Treatment** | **Subgroup** | **Sample size** | **Starting dose for CDK4/6i, n (%)** | **PFS** | | | **OS** | | |
| --- | --- | --- | --- | --- | --- | --- | --- | --- | --- | --- |
|  |  |  |  |  | Median (95% CI), months | HR (95% CI);  P value | At latest time point, n (%) | Median (95% CI), months | HR (95% CI);  P value | At latest time point, n (%) |
| DeMichele 2021;    661-Brufsky-2022 | Palbociclib + letrozole | Lung/liver metastases  (sIPTW) | 321 | NR | 16.1  (13–20.2) | Lung/liver metastases (sIPTW): 0.56 (0.45-0.69); P<0.001  Lung/liver metastases (PSM): 0.57 (0.44-0.72); P<0.001  Lung metastases: 0.52 (0.40-0.69); P<0.001  Liver metastases: 0.70 (0.44-1.10) P=0.12 | NR | Not reached (38.3–not reached) | Lung/liver metastases (sIPTW): 0.58 (0.43-0.77); P<0.001  Lung/liver metastases (PSM): 0.53 (0.39-0.74); P<0.001  Lung metastases: 0.60 (0.41-0.88); P<0.001  Liver metastases: 0.56 (0.33-0.94); P=0.03 | NR |
|  |  | Lung/liver metastases  (PSM) | 194 | NR | 15.7  (12.7–20.2) |  | NR | Not reached (42.7–not reached) |  | NR |
|  |  | Lung metastases | 257 | NR | 16.5  (14–21.9) |  | NR | Not reached |  | NR |
|  |  | Liver metastases | 123 | NR | 10.7  (7.9–12.7) |  | NR | 30.1  (24.3–42.7) |  | NR |
|  | Letrozole | Lung/liver metastases  (sIPTW) | 269 | NR | 9.6  (7.2–11.0) |  | NR | 32.4  (26–40.8) |  | NR |
|  |  | Lung/liver metastases  (PSM) | 194 | NR | 9.5  (6.7–10.8) |  | NR | 29.1  (25.3–40.8) |  | NR |
|  |  | Lung metastases | 171 | NR | 10.5  (8–12.3) |  | NR | 40.2 (29– not reached) |  | NR |
|  |  | Liver metastases | 75 | NR | 8  (4.5–10.7) |  | NR | 16.8  (11.8–25.3) |  | NR |
| DeMichele 2021;    1265-DeMichele-2021 | Palbociclib + letrozole | Visceral disease | 320 | NR | NR | Visceral disease: 0.55 (0.41-0.74); NR  No visceral disease: 0.60 (0.52-0.71); NR  Bone-only disease: 0.55 (0.45-0.68), NR  No bone-only disease: 0.60 (0.49-0.74), NR  Brain metastases: 0.72 (0.22-2.32); NR  No brain metastases: 0.59 (0.52-0.68); NR  Older patients (>=70 years): 0.58 (0.46-0.74); NR  Black patients: 0.35 (0.22-0.57); NR  ECOG score 0: 0.62 (0.47-0.83); NR  ECOG score >=1: 0.54 (0.39-0.74); NR | NR | NR | Visceral disease: 0.58 (0.42-0.79); NR  No visceral disease: 0.73 (0.58-0.92); NR  Bone-only disease: 0.72 (0.53-0.97); NR  No bone-only disease: 0.63 (0.48-0.84); NR  Brain metastases: 0.64 (0.25-1.61); NR  No brain metastases: 0.69 (0.57-0.85); NR  Older patients (>=70 years): 0.55 (0.40-0.77); NR  Black patients: 0.45 (0.25-0.79); NR  ECOG score 0: 0.65 (0.43-0.98); NR  ECOG score >=1: 0.68 (0.48-0.96); NR | NR |
|  |  | No visceral disease | 518 | NR | NR |  | NR | NR |  | NR |
|  |  | Bone-only disease | 309 | NR | NR |  | NR | NR |  | NR |
|  |  | No bone-only disease | 530 | NR | NR |  | NR | NR |  | NR |
|  |  | Brain metastases | 19 | NR | NR |  | NR | NR |  | NR |
|  |  | No brain metastases | 820 | NR | NR |  | NR | NR |  | NR |
|  |  | Older patients (>=70 years) | 356 | NR | NR |  | NR | NR |  | NR |
|  |  | Black patients | 63 | NR | NR |  | NR | NR |  | NR |
|  |  | ECOG score 0 | 271 | NR | NR |  | NR | NR |  | NR |
|  |  | ECOG score >=1 | 257 | NR | NR |  | NR | NR |  | NR |
|  | Letrozole alone | Visceral disease | 269 | NR | NR |  | NR | NR |  | NR |
|  |  | No visceral disease | 429 | NR | NR |  | NR | NR |  | NR |
|  |  | Bone-only disease | 258 | NR | NR |  | NR | NR |  | NR |
|  |  | No bone-only disease | 440 | NR | NR |  | NR | NR |  | NR |
|  |  | Brain metastases | 42 | NR | NR |  | NR | NR |  | NR |
|  |  | No brain metastases | 656 | NR | NR |  | NR | NR |  | NR |
|  |  | Older patients (>=70 years) | 298 | NR | NR |  | NR | NR |  | NR |
|  |  | Black patients | 55 | NR | NR |  | NR | NR |  | NR |
|  |  | ECOG score 0 | 227 | NR | NR |  | NR | NR |  | NR |
|  |  | ECOG score >=1 | 210 | NR | NR |  | NR | NR |  | NR |
| P-REALITY-X;    4379-Brufsky-2023 | Palbociclib + AI | Older adults  (sIPTW) | 371 | 125 mg: 230 (NR)  100 mg: 53 (NR)  75 mg: 23 (NR | 20  (15.7–26.7) | 0.72 (0.59-0.89); P=0.0021 | NR | 43  (40.1–NE) | 0.66 (0.51-0.84); *P*=0.0007 | NR |
|  | AI | Older adults  (sIPTW) | 287 | NR | 15  (12.9–16.8) |  | NR | 32.4  (28.2–38.2) |  | NR |
| P-REALITY X;      4881-Brufsky-2023 | Palbociclib + AI | Lung metastases (sIPTW) | 356 | NR | 20.2  (15.7–27.3) | Lung metastases: 0.55 (0.44-0.69); P<0.0001  Liver metastases: 0.57 (0.42–0.77); P=0.0002  Lung and/or liver metastases: 0.57 (0.48-0.69); P<0.0001 | NR | Not reached  (46.9–NE) | Lung metastases: 0.62 (048.-0.81); P=0.0005  Liver metastases: 0.73 (0.521.01); P=0.0556  Lung and/or liver metastases: 0.64 (0.52-0.79); P<0.0001 | NR |
|  |  | Liver metastases (sIPTW) | 237 | NR | 9.9  (7.5–11.5) |  | NR | 31.4  (25.1–37.8) |  | NR |
|  |  | Lung and/or liver metastases (sIPTW) | 480 | NR | 17  (14.2–20.0) |  | NR | 49.3  (39.5–NE) |  | NR |
|  | AI | Lung metastases (sIPTW) | 292 | NR | 13.5  (10.5–17.4) |  | NR | 35.7  (30.3–50.8) |  | NR |
|  |  | Liver metastases (sIPTW) | 179 | NR | 5.6  (3.4–8.7) |  | NR | 21.4  (14.8–31.6) |  | NR |
|  |  | Lung and/or Liver metastases (sIPTW) | 411 | NR | 10.7  (8.9–12.9) |  | NR | 31.5  (28.5–36.8) |  | NR |
| P-REALITY-X;    SABCS23-018-Brufsky-2023 | Palbociclib + AI | Cardiovascular disease  (sIPTW) | 192 | NR | 20  (11.7–27.5) | 10.68 (0.51−0.90); P=0.007 | NR | 40.7  (30.9–56.0) | 0.73 (0.54−0.10); P=0.048 | NR |
|  | AI | Cardiovascular disease  (sIPTW) | 144 | NR | 12.5  (9.7–18.3) |  | NR | 26.5  (23.3–37.3) |  | NR |
| Rugo 2023a;    4549-Rugo-2023 | Palbociclib + AI | African American | 127 | 125 mg: 110 (86.6)  100 mg: 8 (6.3)  75 mg: 7 (5.5) | 18  (12.4–26.7) | 0.72  (0.48-1.07);  *P* = 0.102 | 20 months: NR (46.6) | Not reached  (38.2-NR) | 0.54  (0.35-0.84);  *P* = .007 | 36 months: NR (61.2) |
|  | AI | African American | 143 | NR | 10.5  (7.0–13.4) |  | 20 months: NR (30.1) | 28.2  (19.2-52.8) |  | 36 months: NR (44.3) |
| Rugo 2023b;    3494-Rugo-2023 | Palbociclib + letrozole | Older adults  (sIPTW) | 450 | NR | 22.2  (12.9–18.9) | 0.59  (0.47-0.74); *P<*0.001 | NR | Not reached | 0.55  (0.42-0.72); *P*<0.001 | NR |
|  | Letrozole | Older adults  (sIPTW) | 335 | NR | 15.8  (20.0–30.4) |  | NR | 43.4  (30.0–NE) |  | NR |

AI = aromatase inhibitor; CDK4/6i = cyclin-dependent kinase 4/6 inhibitors; CI = confidence interval; NE = not evaluable; NR = not reported; OS = overall survival; PFS = progression-free survival; PSM = propensity score matching; RWE = real-world evidence; sIPTW = stabilized inverse probability of treatment weighting.

**Supplementary Table 5.** Effectiveness outcomes for prespecified subgroups of interest receiving first-line ribociclib in comparative RWE studies versus ET.

| **Study name; reference** | **Treatment**  **(line of therapy)** | **Subgroup** | **Sample size** | **Starting dose for CDK4/6i, n (%)** | **PFS** | | | **OS** | | |
| --- | --- | --- | --- | --- | --- | --- | --- | --- | --- | --- |
|  |  |  |  |  | Median (95% CI), months | HR (95% CI);  P value | At latest time point, n (%) | Median (95% CI), months | HR (95% CI);  P value | At latest time point, n (%) |
| RIBANNA;    ESMO23-044- Woeckel-2023 | Ribociclib + AI/fulvestrant | Liver metastases | 384 | NR | 16.6  (14.2–20.6) | Liver metastases: 1.48 (0.80-2.75); P = 0.2134  RIB + AI/FUL de novo vs RIB + AI/FUL recurrent: 1.42 (1.20-1.69); P < 0.0001  RIB + AI/FUL de novo vs ET/de novo: 1.30 (0.87-1.94); P = 0.2046  RIB + AI/FUL de novo vs ET/recurrent: 1.66 (1.17-2.37); P = 0.0045 | 24 months: 105 (33.65) | NR | NR | NR |
|  |  | No liver metastases | 1427 | NR | 36.6  (33.6–41.5) |  | 24 months:  585 (57.3) | NR |  | NR |
|  | ET alone | Liver metastases | 23 | NR | 10.4  (3.5–NA) |  | 24 months:  2 (14.29) | NR |  | NR |
|  |  | No liver metastases | 169 | NR | 37.6  (26.5–53.1) |  | 24 months:  67 (54.03) | NR |  | NR |
|  | CT alone | Liver metastases | 65 | NR | 13.5  (6.7–17.1) | NR | 24 months:  9 (17.31) | NR | NR | NR |
|  |  | No liver metastases | 78 | NR | 16.8  (9.5–22.5) |  | 24 months:  14 (25.45) | NR |  | NR |

AI = aromatase inhibitor; CDK4/6i = cyclin-dependent kinase 4/6 inhibitors; CI = confidence interval; CT = chemotherapy; ET = endocrine therapy; NR = not reported; OS = overall survival; PFS = progression-free survival; RWE = real-world evidence.

Comparative RWE Studies: CDK4/6i versus CDK4/6i

**Supplementary Table 6.** Effectiveness outcomes for prespecified subgroups of interest receiving first-line comparative RWE studies assessing two or more specified CDK4/6i.

| **Study name; reference** | **Treatment** | **Subgroup** | **Sample size** | **Starting dose for CDK4/6i, n (%)** | **PFS** | | | **OS** | | |
| --- | --- | --- | --- | --- | --- | --- | --- | --- | --- | --- |
|  |  |  |  |  | Median (95% CI), months | HR (95% CI);  P value | At latest time point, n (%) | Median (95% CI), months | HR (95% CI);  P value | At latest time point, n (%) |
| 3400-Tang-2023 | Palbociclib + AI | Recurrent disease cohort | NR | NR | NR | NR | NR | 48.4 (NR) | Recurrent disease: 1.12 (0.63-1.98); P=0.69  De novo disease: 0.83 (0.17-4.05); P=0.80 | NR |
|  |  | *De novo* disease | NR | NR | NR |  | NR | 77.4 (NR) |  | NR |
|  | Ribociclib + AI | Recurrent disease cohort | NR | NR | NR |  | NR | 40.4 (NR) |  | NR |
|  |  | *De novo* disease | NR | NR | NR |  | NR | Not reached |  | NR |
| 4112-Cejuela-2023 | CDK4/6i + ET | Endocrine resistant | 78 | NR | 18.30 (NR) | Palbociclib/endocrine sensitive vs abemaciclib/endocrine sensitive: 2.41 (1.09-5.31); P = 0.029  Ribociclib/endocrine sensitive vs abemaciclib/endocrine sensitive: 2.19 (0.91-5.31); P = 0.091 | NR | NR | NR | NR |
|  |  | Endocrine sensitive | NR | NR | Not reached |  | NR | NR |  | NR |
|  |  | Visceral disease | 116 | NR | 37.81  (25.93–49.70) |  | NR | NR |  | NR |
|  |  | Nonvisceral disease | 90 | NR | 31.14  (19.61–42.67) |  | NR | NR |  | NR |
|  | Abemaciclib + ET | Dose reduction | 30 | NR | Not reached |  | NR | NR |  | NR |
|  |  | Endocrine resistant | 21 | NR | Not reached |  | NR | NR |  | NR |
|  |  | Visceral disease | 34 | NR | 39.49 (NR) |  | NR | NR |  | NR |
|  |  | Nonvisceral disease | 22 | NR | Not reached |  | NR | NR |  | NR |
|  | Palbociclib + ET | Dose reduction | 50 | NR | 35.87 (NR) |  | NR | NR |  | NR |
|  |  | Endocrine resistant | 37 | NR | 17.02 (NR) |  | NR | NR |  | NR |
|  |  | Visceral disease | 50 | NR | Not reached |  | NR | NR |  | NR |
|  |  | Nonvisceral disease | 46 | NR | 24.1  (18.04–30.12) |  | NR | NR |  | NR |
|  | Ribociclib + ET | Dose reduction | 28 | NR | 36.01 (NR) |  | NR | NR |  | NR |
|  |  | Endocrine resistant | 20 | NR | 10.38 (NR) |  | NR | NR |  | NR |
|  |  | Visceral disease | 32 | NR | 23.16  (13.58–32.74) |  | NR | NR |  | NR |
|  |  | Nonvisceral disease | 22 | NR | 36.01 (NR) |  | NR | NR |  | NR |
| 4506-Tang-2023 | CDK4/6i + ET | *De novo* | 97 | NR | 47.1 (NR) | NR | 5 years:  NR (34.7) | 77.4 (NR) | NR | 5 years:  NR (65.84) |
|  |  | Recurrent | 130 | NR | 20.3 (NR) |  | 5 years:  NR (15.37) | 37.3 (NR) |  | 5 years:  NR (36.91) |
|  |  | ER+/PR+ | 160 | NR | 38 (NR) |  | 5 years:  NR (26.73) | 62.6 (NR) |  | 5 years:  NR (51.37) |
|  |  | ER+/PR- | 67 | NR | 19.2 (NR) |  | 5 years:  NR (20.27) | 34.3 (NR) |  | 5 years:  NR (38.12) |
|  |  | ≤65 years | 97 | NR | 28 (NR) |  | 5 years:  NR (22.79) | 77.4 (NR) |  | 5 years:  NR (52) |
|  |  | 66–79 years | 95 | NR | 29 (NR) |  | 5 years:  NR (27.73) | 61.7 (NR) |  | 5 years:  NR (51.16) |
|  |  | ≥80 years | 35 | NR | 21.3 (NR) |  | 5 years:  NR (11.89) | 35 (NR) |  | 5 years:  NR (32.28) |
|  | Palbociclib + ET | *De novo* | 74 | NR | 43.6 (NR) |  | 5 years:  NR (31.43) | 77.4 (NR) |  | 5 years:  NR (62.38) |
|  |  | Recurrent | 88 | NR | 20.9 (NR) |  | 5 years:  NR (16.69) | 36.1 (NR) |  | 5 years:  NR (37.98) |
|  |  | ER+/PR+ | 114 | NR | 30.2 (NR) |  | 5 years:  NR (22.66) | 62.6 (NR) |  | 5 years:  NR (50.41) |
|  |  | ER+/PR- | 48 | NR | 21.3 (NR) |  | 5 years:  NR (21.07) | 49.5 (NR) |  | 5 years:  NR (39.48) |
|  |  | ≤65 years | 62 | NR | 30.2 (NR) |  | 5 years:  NR (25.56) | 77.4 (NR) |  | 5 years:  NR (55.27) |
|  |  | 66–79 years | 75 | NR | 28.2 (NR) |  | 5 years:  NR (25.61) | 61.7 (NR) |  | 5 years:  NR (50.17) |
|  |  | ≥80 years | 25 | NR | 14.5 (NR) |  | 5 years:  NR (0) | 29.6 (NR) |  | 5 years:  NR (23.34) |
|  | Ribociclib + ET | *De novo* | 12 | NR | NR |  | 5 years:  NR (74.07) | NR |  | 5 years:  NR (82.5) |
|  |  | Recurrent | 34 | NR | 18.85 (NR) |  | 5 years:  NR (19.1) | 44.6 (NR) |  | 5 years:  NR (27.98) |
|  |  | ER+/PR+ | 31 | NR | 44 (NR) |  | 5 years:  NR (41.82) | 54.8 (NR) |  | 5 years:  NR (45.37) |
|  |  | ER+/PR- | 15 | NR | 10.1 (NR) |  | 5 years:  NR (13.33) | 34.3 (NR) |  | 5 years:  NR (40) |
|  |  | ≤65 years | 25 | NR | 20.5 (NR) |  | 5 years:  NR (27.93) | 44.6 (NR) |  | 5 years:  NR (36.86) |
|  |  | 66–79 years | 16 | NR | 24.7 (NR) |  | 5 years:  NR (34.29) | 54.8 (NR) |  | 5 years:  NR (49.36) |
|  |  | ≥80 years | 5 | NR | 68.2 (NR) |  | 5 years:  NR (60) | NR (NR) |  | 5 years:  NR (68.86) |
|  | Abemaciclib + ET | *De novo* | 11 | NR | NR |  | 5 years:  NR | NR |  | 5 years:  NR |
|  |  | Recurrent | 8 | NR | NR |  | 5 years:  NR | 30.7 (NR) |  | 5 years:  NR |
|  |  | ER+/PR+ | 15 | NR | NR |  | 5 years:  NR | NR |  | 5 years:  NR |
|  |  | ER+/PR- | 4 | NR | NR |  | 5 years:  NR | 30.2 (NR) |  | 5 years:  NR |
|  |  | ≤65 years | 8 | NR | NR |  | 5 years:  NR | 36.5 (NR) |  | 5 years:  NR |
|  |  | 66–79 years | 6 | NR | NR |  | 5 years:  NR | NR |  | 5 years:  NR |
|  |  | ≥80 years | 5 | NR | NR |  | 5 years:  NR | NR |  | 5 years:  NR |
| ESMO23-066-Lenza-2023 | Palbociclib + ET | <2 year hormone therapy | NR | NR | 8 (NR) | NR | NR | 22 (NR) | NR | NR |
|  |  | Visceral | NR | NR | NR |  | NR | 29 (NR) |  | NR |
|  | Ribociclib + ET | <2 year hormone therapy | NR | NR | 6 (NR) |  | NR | 28 (NR) |  | NR |
|  |  | Visceral | NR | NR | NR |  | NR | 53 (NR) |  | NR |
|  | Abemaciclib + ET | <2 year hormone therapy | NR | NR | 12 (NR) |  | NR | NR |  | NR |
|  |  | Visceral | NR | NR | NR |  | NR | NR |  | NR |

AI = aromatase inhibitor; CDK4/6i = cyclin-dependent kinase 4/6 inhibitors; CI = confidence interval; ER = estrogen receptor; ET = endocrine therapy; NR = not reported; OS = overall survival; PFS = progression-free survival; PR = progesterone receptor; RWE = real-world evidence.

APPENDIX J: Effectiveness of any first-line CDK4/6i regimen versus ET in comparative studies

Progression-free survival

The PFS data for patients receiving any CDK4/6i regimen versus ET were reported in three comparative studies (**Figure 1B**), with all studies providing results for the broader population (**Table 4**). In two comparative studies, regimens that included any CDK4/6i plus ET demonstrated greater benefits relative to control treatment with ET alone, resulting in a median PFS of 27.0 (n=NR)^8^ and 31.1 months (n=77)^9^ for CDK4/6i and 17.0 (n=NR)^8^ to 21.9 months (n=130)^9^ for the control. Similarly, the third study reported improved median PFS with regimens that included either palbociclib or abemaciclib (30.0 months; n=41) compared with any non-CDK4/6i regimen (13.0 months; n=67)^10^ (**Table 4**). Notably, none of the studies reported PFS data for specific patient subgroups.

Overall survival

The OS data for patients receiving any CDK4/6i regimen versus ET were reported in three comparative studies (**Figure 1B**). All studies reported results for the broader population (**Table 4**), with no data reported for subgroup populations. In a US-based study, regimens that included any CDK4/6i demonstrated improved survival rates at 36 months compared with ET alone (73.0% [n=169] vs. 49.1% [n=461]).^11^ However, longer follow-up data from a conference abstract for the BRAVE study in Brazil indicated more comparable survival rates at 4 years between the CDK4/6i regimens and ET alone (71.3% [n=77] vs. 74.4% [n=130]).^9^ Lastly, in a conference abstract from the US, regimens that included any CDK4/6i (n=NR) showed statistically significant improvements in median OS compared with ET alone (n=NR) (52.0 vs. 42.0 months; HR: 0.74 [95% CI: NR]; P<0.001).^8^

**Supplemental Table 1.** Effectiveness outcomes for any first-line CDK4/6i in comparative RWE studies versus ET.

| **Study name; reference** | **Treatment**  **(line of therapy)** | **Subgroup** | **Sample size** | **Starting dose for CDK4/6i, n (%)** | **PFS** | | | **OS** | | |
| --- | --- | --- | --- | --- | --- | --- | --- | --- | --- | --- |
|  |  |  |  |  | Median (95% CI), months | HR (95% CI);  P value | At latest time point, n (%) | Median (95% CI), months | HR (95% CI);  P value | At latest time point, n (%) |
| BRAVE;  SABCS23-114-  Werutsky-2023 | CDK4/6i + ET | All patients | 77 | NR | 31.1  (15.8–42.8) | NR | NR | NR | NR | 4 years: NR (71.3) |
|  | ET alone | All patients | 130 | NR | 21.9  (17.3–25.5) |  | NR | NR |  | 4 years:  NR (74.4) |
|  | CT alone | All patients | 55 | NR | 13.6  (6.1–39.0) |  | NR | NR |  | 4 years:  NR (50.3) |
|  | CT followed by ET alone | All patients | 42 | NR | 18.4  (12.1–22.1) |  | NR | NR |  | 4 years: NR (39) |
| 556-Endo-2022 | Palbociclib or abemaciclib + AI or tamoxifen | All patients | 41 | NR | 30 (NR) | NR | NR | NR | NR | NR |
|  | Therapy other than CDK4/6i (unspecified) | All patients | 67 | NR | 13 (NR) |  | NR | NR |  | NR |
| 3924-Goyal-2023 | CDK4/6i + ET | All patients | 169 | NR | NR | NR | NR | NE  (NE–NE) | 0.59 (0.42-0.82); NR | 36 months:  NR (73) |
|  | ET alone | All patients | 461 | NR | NR |  | NR | 34.8  (29.2–NE) |  | 36 months:  NR (49.1) |
| ESMO23-092-Kimmick-2023 | CDK 4/6i + ET | IPW | NR | NR | 27 (NR) | 0.61 (NR); P <0.001 | NR | 52 (NR) | 0.74 (NR); P <0.001 | NR |
|  | ET alone | IPW | NR | NR | 17 (NR) |  | NR | 42 (NR) |  | NR |

AI = aromatase inhibitor; CDK4/6i = cyclin-dependent kinase 4/6 inhibitors; CI = confidence interval; CT = chemotherapy; ET = endocrine therapy; IPTW = inverse probability of treatment weight; NE = not evaluable; NR = not reported; OS = overall survival; PFS = progression-free survival; RWE = real-world evidence.

APPENDIX K: Effectiveness of CDK4/6i versus CDK4/6i: Comparative results of CDK4/6i and different backbone therapies

The following section describes the comparative studies that evaluated the same CDK4/6i in combination with different backbone therapies (e.g., palbociclib plus AI vs. palbociclib plus fulvestrant). Of note, no studies compared abemaciclib-based regimens.

Progression-free survival

Palbociclib versus palbociclib

The PFS data comparing palbociclib regimens with various backbone therapies were reported in six studies (**Figure 1C**). Four studies reported results for the broader population (**Supplemental Table 1**), and two focused on specific patient subgroup populations (**Supplemental Table 2**).

**Broader population**

In three comparative studies, palbociclib plus AI demonstrated superior PFS benefit compared with palbociclib plus fulvestrant, with a greater proportion of patients experiencing PFS at 24 months (range: 63.1%^12^ to 65.7%^13^ vs. 48.9%^12^ to 53.5%^13^). Median PFS results were more comparable in one study from Italy, which reported 15.1 months for palbociclib plus AI and 13.5 months for palbociclib plus fulvestrant, though these findings were based on notably small sample sizes (32 and 29 patients, respectively).^14^ The fourth comparative study evaluated palbociclib plus AI or fulvestrant with or without concomitant proton pump inhibitor (PPI) and found that median PFS was improved in patients who did not receive concomitant PPIs compared with those who did (44 months vs. 14 months)^15^ (**Supplemental Table 1**).

**Subgroups**

One Chinese study evaluated subgroups based on the starting dose of palbociclib and reported improved median PFS among patients who received 125 mg of palbociclib initially compared with those who received 100mg (range: 15.5 to 24.9 months vs. 10.6 to 17.3 months).^16^ The second study compared patients with endocrine-resistant disease with those with endocrine-sensitive disease in Denmark and found that median PFS was higher in patients who are endocrine sensitive (range: 19.9 to 31.3 months) than those who are endocrine resistant (range: 12 to 21.5 months).^17^ Both studies demonstrated noteworthy improvements in median PFS when treated with palbociclib plus AI versus palbociclib plus fulvestrant (**Supplemental Table 2**).

Ribociclib versus ribociclib

The PFS data comparing ribociclib plus AI versus ribociclib plus fulvestrant in the broader population were reported in four studies (**Figure 1C**). Across these studies, ribociclib plus AI consistently demonstrated superior PFS outcomes compared with ribociclib plus fulvestrant, with median PFS ranging from 28.6^18^ to 31 months^19^ and 9.1^18^ to 16.8 months,^19^ respectively. Additionally, a conference abstract from the RIBANNA study compared 24-month PFS rates across several patient cohorts resembling those in RCTs. The highest PFS rate observed was 58.4% in a regimen of ribociclib plus AI (mimicking MONALEESA-2), whereas the lowest was 37% in a regimen of ribociclib plus fulvestrant (mimicking MONALEESA-3).^20^ The fourth study evaluated ribociclib plus AI or fulvestrant with or without concomitant PPI and found that median PFS was improved in patients who did not receive concomitant PPIs compared with those who did (49.2 months vs. 35.3 months)^21^ (**Supplemental Table 3**). Notably, none of the studies reported PFS data for specific patient subgroups.

Regimens including any CDK4/6i

The PFS data comparing regimens, including any CDK4/6i with different backbone therapies, were reported in two studies focused on subgroups not prespecified as those of interest, such as histology and *BRCA* mutation. These results are summarized in **Supplemental Table 4**. Notably, neither of the studies reported PFS data for the broader population or the prespecified subgroups of interest.

Overall survival

Palbociclib versus palbociclib

The OS data comparing palbociclib regimens with various backbone therapies were reported in four studies (**Figure 1C**). Three studies reported results for the broader population (**Supplemental Table 1**), and one focused on specific patient subgroup populations (**Supplemental Table 2**).

**Broader population**

In two comparative studies, palbociclib plus AI and palbociclib plus fulvestrant showed similar OS rates at 24 months, with palbociclib plus AI ranging from 90.6%^12^ to 90.8%^13^ and palbociclib plus fulvestrant ranging from 92.7%^12^ to 92.8%.^13^ In one Italian study, the median OS was 28 months with palbociclib plus AI or fulvestrant after 24 months of median follow-up^14^ (**Supplemental Table 1**).

**Subgroups**

The Denmark-based study that evaluated PFS among patients with endocrine-resistant versus endocrine-sensitive disease also reported OS data. The OS results were consistent with the PFS trends observed, with higher median OS in patients with endocrine-sensitive disease compared with those with endocrine-resistant disease. Additionally, greater improvements in median OS were seen with palbociclib plus AI compared to palbociclib plus fulvestrant, mirroring the PFS findings (**Supplemental Table 2**).^17^

Ribociclib versus ribociclib

Among studies comparing ribociclib plus AI to ribociclib plus fulvestrant in the broader population, only one reported OS data (**Figure 1C**). This study from Jordan, presented in a conference abstract, indicated that the median OS had not been reached after a median follow-up of 31.1 months (**Supplemental Table 3**).^18^ No OS data for specific patient subgroups were reported.

Regimens including any CDK4/6i

The same two studies that reported comparative PFS data for regimens, including any CDK4/6i with different backbone therapies also provided OS data. These studies focused on subgroups such as histology and *BRCA* mutation rather than the broader population or prespecified subgroups of interest. The OS results are summarized in **Supplemental Table 4.**

**Supplemental Table 1.** Effectiveness outcomes for overall first-line palbociclib in comparative RWE studies versus palbociclib.

| **Study name; reference** | **Treatment** | **Subgroup** | **Sample size** | **Starting dose for CDK4/6i, n (%)** | **PFS** | | | **OS** | | |
| --- | --- | --- | --- | --- | --- | --- | --- | --- | --- | --- |
|  |  |  |  |  | Median (95% CI), months | HR (95% CI);  P value | At latest time point, n (%) | Median (95% CI), months | HR (95% CI);  P value | At latest time point, n (%) |
| IRIS: Europe, North and South America, and Asia;  261-Mycock-2021 | Palbociclib + AI | All patients | 1,621 | 125 mg: 1,432 (88.3) 100 mg: 149 (9.2)  75 mg: 40 (2.5) | NR | NR | 24 months: NR (65.7) | NR | NR | 24 months: NR (90.8) |
|  | Palbociclib + fulvestrant | All patients | 552 | 125 mg: 479 (86.8) 100 mg: 57 (10.3)  75 mg: 16 (2.9) | NR |  | 24 months: NR (53.5) | NR |  | 24 months: NR (92.8) |
| IRIS Europe II;    1901-Mycock-2021 | Palbociclib + AI | All patients | 925 | NR | NR | NR | 24 months: 583 (63.1) | NR | NR | 24 months: 833 (90.6) |
|  | Palbociclib + fulvestrant | All patients | 379 | NR | NR |  | 24 months: 185 (48.9) | NR |  | 24 months: 336 (92.7) |
| 1067-Palumbo-2021 | Palbociclib + letrozole or fulvestrant | All patients | 61 | NR | 14  (9.5–25) | NR | NR | 28 (NR) | NR | NR |
|  | Palbociclib + letrozole | All patients | 32 | NR | 15.1  (7.2–25) |  | NR | NR |  | NR |
|  | Palbociclib + fulvestrant | All patients | 29 | NR | 13.5  (6.5–18) |  | NR | NR |  | NR |
| 4060-Criado-2023 | Palbociclib + AI/fulvestrant ± PPI | All patients | NR | NR | 36 (NR) | NR | NR | NR | NR | NR |
|  | Palbociclib + AI/fulvestrant + PPI | All patients | NR | NR | 14 (NR) |  | NR | NR |  | NR |
|  | Palbociclib + AI/fulvestrant | All patients | NR | NR | 44 (NR) |  | NR | NR |  | NR |

AI = aromatase inhibitor; CDK4/6i = cyclin-dependent kinase 4/6 inhibitors; CI = confidence interval; NR = not reported; OS = overall survival; PFS = progression-free survival; PPI = proton pump inhibitor; RWE = real-world evidence.

**Supplemental Table 2.** Effectiveness outcomes for prespecified subgroups of interest receiving first-line palbociclib in comparative RWE studies versus palbociclib.

| **Study name; reference** | **Treatment** | **Subgroup** | **Sample size** | **Starting dose for CDK4/6i, n (%)** | **PFS** | | | **OS** | | |
| --- | --- | --- | --- | --- | --- | --- | --- | --- | --- | --- |
|  |  |  |  |  | Median (95% CI), months | HR (95% CI);  P value | At latest time point, n (%) | Median (95% CI), months | HR (95% CI);  P value | At latest time point, n (%) |
| 1624-Zhang-2021 | Palbociclib + AI or fulvestrant | Palbociclib 100 mg initially | 26 | 100 mg: 26 (100) | 12.3 (NR) | NR | NR | NR | NR | NR |
|  |  | Palbociclib 125 mg initially | 62 | 125 mg: 62 (100) | 20.9 (NR) |  | NR | NR |  | NR |
|  | Palbociclib + AI | Palbociclib 100 mg initially | 15 | 100 mg: 15 (100) | 17.3  (1.63–32.96) |  | NR | NR |  | NR |
|  |  | Palbociclib 125 mg initially | 41 | 125 mg: 41 (100) | 24.9 (NR) |  | NR | NR |  | NR |
|  | Palbociclib + fulvestrant | Palbociclib 100 mg initially | 11 | 100 mg: 11 (100) | 10.6  (2.88– 18.26) |  | NR | NR |  | NR |
|  |  | Palbociclib 125 mg initially | 21 | 125 mg: 21 (100) | 15.5  (2.19– 28.75) |  | NR | NR |  | NR |
| 3954-Garly-2023 | Palbociclib + AI | Endocrine resistant | 44 | NR | 21.5  (14.9–37.2) | NR | NR | 43.5  (31.4–NA) | NR | NR |
|  |  | Endocrine sensitive | 423 | NR | 31.3  (28.2–34.6) |  | NR | 56.9  (52.5–NA) |  | NR |
|  | Palbociclib + fulvestrant | Endocrine resistant | 101 | NR | 12  (8.4–17.6) |  | NR | 28.8  (24.5–32.1) |  | NR |
|  |  | Endocrine sensitive | 158 | NR | 19.9  (16.5–24.3) |  | NR | 43.6  (37.9–NA) |  | NR |

AI = aromatase inhibitor; CDK4/6i = cyclin-dependent kinase 4/6 inhibitors; CI = confidence interval; NE = not evaluable; NR = not reported; OS = overall survival; PFS = progression-free survival; RWE = real-world evidence.

**Supplemental Table 3.** Effectiveness outcomes for overall first-line ribociclib in comparative RWE studies versus ribociclib.

| **Study name; reference** | **Treatment**  **(line of therapy)** | **Subgroup** | **Sample size** | **Starting dose for CDK4/6i, n (%)** | **PFS** | | | **OS** | | |
| --- | --- | --- | --- | --- | --- | --- | --- | --- | --- | --- |
|  |  |  |  |  | Median (95% CI), months | HR (95% CI);  P value | At latest time point, n (%) | Median (95% CI), months | HR (95% CI);  P value | At latest time point, n (%) |
| REACHAUT;    SABCS23-117-Singer-2023 | Ribociclib + AI/fulvestrant | All patients | 281 | NR | 29.7  (24.1–35.4) | NR | NR | NR | NR | NR |
|  | Ribociclib + AI | All patients | 210 | NR | 31  (26.9–36.0) |  | NR | NR |  | NR |
|  | Ribociclib + fulvestrant | All patients | 39 | NR | 16.8  (6.1–35.4) |  | NR | NR |  | NR |
| RIBANNA;    SABCS22-089-Jackisch-2022 | Ribociclib + letrozole | MONALEESA-2-like RIBANNA population | 553 | NR | NR | NR | 24 months: 323 (58.4) | NR | NR | NR |
|  | Ribociclib + fulvestrant | MONALEESA-3-like RIBANNA population | 192 | NR | NR |  | 24 months: 71 (37.0) | NR |  | NR |
|  | Ribociclib + letrozole or anastrozole | MONALEESA-7-like RIBANNA population | 57 | NR | NR |  | 24 months: 27 (46.5) | NR |  | NR |
|  | Ribociclib + AI or fulvestrant | Other* | 1,009 | NR | NR |  | 24 months: 514 (51.0) | NR |  | NR |
| 576-Del Re-2022 | Ribociclib + letrozole or fulvestrant | All patients | 78 | 600 mg: 78 (100) | 49.2 (NR) | 1.18 (0.64-2.15); P = 0.59 | NR | NR | NR | NR |
|  | Ribociclib + letrozole or fulvestrant + PPI | All patients | 50 | 600 mg: 50 (100) | 35.3 (NR) |  | NR | NR |  | NR |
| SABCS23-105-Sharaf-2023 | Ribociclib + AI/fulvestrant | All patients | 257 | NR | 19.3  (17.6– 23.3) | NR | NR | Not reached | NR | NR |
|  | Ribociclib + AI | All patients | 134 | NR | 28.6  (17.8– 23.1) |  | NR | NR |  | NR |
|  | Ribociclib + Fulvestrant | All patients | 20 | NR | 9.1  (4.4– not reached) |  | NR | NR |  | NR |
|  | Ribociclib + ET | All patients | 162 | NR | 23.1  (19.7– not reached) |  | NR | NR |  | NR |

*Other refers to a population that does not mimic those of the MONALEESA clinical trials.

AI = aromatase inhibitor; CDK4/6i = cyclin-dependent kinase 4/6 inhibitors; CI = confidence interval; ET = endocrine therapy; NR = not reported; OS = overall survival; PFS = progression-free survival; PPI = proton pump inhibitor; RWE = real-world evidence.

**Supplemental Table 4.** Effectiveness outcomes for other subgroups receiving any first-line CDK4/6i regimen in comparative RWE studies versus any CDK4/6i regimen.

| **Study name; reference** | **Treatment** | **Subgroup** | **Sample size** | **Starting dose for CDK4/6i, n (%)** | **PFS** | | | **OS** | | |
| --- | --- | --- | --- | --- | --- | --- | --- | --- | --- | --- |
|  |  |  |  |  | Median (95% CI), months | HR (95% CI);  P value | At latest time point, n (%) | Median (95% CI), months | HR (95% CI);  P value | At latest time point, n (%) |
| 271-Mouabbi-2022 | CDK4/6i + AI | IDC | 829 | NR | 16.0 (NR) | NR | 12 months: 1064 (58) | 38.3 (NR) | NR | 60 months: 315 (38) |
|  |  | ILC | 158 | NR | 18.8 (NR) |  | 12 months: 208 (62) | 35.9 (NR) |  | 60 months: 22 (14) |
|  |  | Mixed | 34 | NR | 16.9 (NR) |  | 12 months: 56 (71) | 34.6 (NR) |  | 60 months: 9 (25) |
|  | CDK4/6i + fulvestrant | IDC | 416 | NR | 8.9 (NR) |  | 12 months: 332 (40) | 28.7 (NR) |  | 60 months: 50 (12) |
|  |  | ILC | 90 | NR | 8.7 (NR) |  | 12 months: 62 (39) | 25.9 (NR) |  | 60 months: 11 (12) |
|  |  | Mixed | 34 | NR | 13.6 (NR) |  | 12 months: 21 (61) | 24.6 (NR) |  | 60 months: 4 (17) |
| 659-Bruno-2022 | Palbociclib or ribociclib + letrozole or fulvestrant | *BRCA1*/2- *ATM*-*CHEK2* pathogenic variant | 7 | NR | 10.2  (5.9–14.5) | BRCA1/2- ATM-CHEK2 versus WT: 0.47 (0.25-0.91); P = 0.025  BRCA1/2- ATM-CHEK2 versus nontested: 0.39 (0.21-0.73); P = 0.003 | NR | NR | BRCA1/2- ATM-CHEK2 versus WT: 0.31 (0.13-0.77); P = 0.011  BRCA1/2- ATM-CHEK2 versus nontested: 0.43 (0.19-0.97); P = 0.0410 | NR |
|  |  | Wild-type | 33 | NR | 15.0  (4.4–25.6) |  | NR | NR |  | NR |
|  | CDK4/6i + letrozole or fulvestrant | Nontested | 107 | NR | 23.3  (14.3–32.3) |  | NR | NR |  | NR |

AI = aromatase inhibitor; ATM = ataxia-telangiectasia mutated; BRCA = breast cancer gene; CDK4/6i = cyclin-dependent kinase 4/6 inhibitors; CHEK2 = checkpoint serine-threonine kinase 2; CI = confidence interval; IDC = invasive duct carcinoma; ILC = invasive lobular carcinoma; NR = not reported; OS = overall survival; PFS = progression-free survival; RWE = real-world evidence.

APPENDIX L: Supplementary references

1. Page MJ, McKenzie JE, Bossuyt PM, et al. The PRISMA 2020 statement: An updated guideline for reporting systematic reviews. *BMJ.* 2021;372:n71.

2. Visani L, Livi L, Ratosa I, et al. Safety of CDK4/6 inhibitors and concomitant radiation therapy in patients affected by metastatic breast cancer. *Radiotherapy and oncology : journal of the European Society for Therapeutic Radiology and Oncology.* 2022;177:40-45.

3. Mónica Cejuela Solís MAC, Ana Gil, Alejandro Falcon, Marta Benavent, Isabel Miras Rodríguez, Manuel Ruiz-Borrego, Sonia Molina-Pinelo, Javier Salvador Bofill. HER2-low expression as a prognostic factor in patients treated with CDK4/6 inhibitors: A retrospective analysis. In:2023.

4. Gad AM, AlSayed A, AlShibani A, Twegieri T, Suleman K, Ajarim D. Clinical outcome and toxicity profile of cyclin dependent kinase 4/6 inhibitors in combination with hormonal treatment in management of metastatic breast cancer patients: A middle-east real world experience. *Cancer Research.* 2023;83(5 Supplement).

5. Amaro CP, Batra A, Lupichuk S. First-line treatment with a cyclin-dependent kinase 4/6 inhibitor plus an aromatase inhibitor for metastatic breast cancer in Alberta. *Current oncology (Toronto, Ont).* 2021;28(3):2270-80.

6. Choong GM, Liddell S, Ferre RAL, et al. Clinical management of metastatic hormone receptor-positive, HER2-negative breast cancer (MBC) after CDK 4/6 inhibitors: A retrospective single-institution study. *Breast cancer research and treatment.* 2022;196(1):229-37.

7. Rottier P, Emile G, Johnson A, et al. Pretreatment neutrophil to lymphocyte ratio as prognostic factor in metastatic breast cancer treated with cyclin dependent kinase 4/6 inhibitors. *Frontiers in Oncology.* 2023;12:1105587.

8. Kimmick G, Pilehvari A, You W, Bonilla G, Anderson R. Disparities in receipt of CDK4/6 inhibitors with endocrine therapy as therapy for hormone receptor-positive, HER2-negative metastatic breast cancer in the real-world setting. In:2023.

9. Werutsky G, Reinert T, Rosa DD, et al. Real-world data on first-line treatment of HR-positive, HER2-negative, metastatic breast cancer in Brazil (BRAVE study/LACOG 0221). *Cancer Research.* 2023;83(5 Supplement).

10. Endo Y, Yoshimura A, Sawaki M, et al. Time to chemotherapy for patients with estrogen receptor-positive breast cancer and cyclin-dependent kinase 4 and 6 inhibitor use. *Journal of breast cancer.* 2022;25(4):296-306.

11. Goyal RK, Chen H, Abughosh SM, Holmes HM, Candrilli SD, Johnson ML. Overall survival associated with CDK4/6 inhibitors in patients with HR+/HER2- metastatic breast cancer in the United States: A SEER-medicare population-based study. *Cancer.* 2023;129(7):1051-63.

12. Mycock K, Zhan L, Hart K, et al. Real-world treatment of patients with palbociclib for HR+/HER2-advanced/metastatic breast cancer: The Europe IRIS study. *Future oncology (London, England).* 2021.

13. Mycock K, Hanson KA, Taylor-Stokes G, et al. Real-world treatment patterns and clinical outcomes associated with palbociclib combination therapy: A multinational, pooled analysis from the Ibrance real world insights study. *Clinical Therapeutics.* 2022;44(12):1588-601.

14. Palumbo R, Sottotetti F, Presti D, et al. Patterns of treatment and outcome of palbociclib plus endocrine therapy in hormone receptor-positive/HER2 receptor-negative metastatic breast cancer: A real-world multicentre Italian study. *Therapeutic Advances in Medical Oncology.* 2021;13.

15. Criado JA, Martinez VM, Aunon MPZ, et al. Concomitant administration of palbociclib and proton pump inhibitors affects clinical outcomes in metastatic breast cancer patients. *ESMO Open.* 2023;8(1 Supplement 4):101431.

16. Zhang L, Song G, Shao B, et al. The efficacy and safety of palbociclib combined with endocrine therapy in patients with hormone receptor-positive HER2-negative advanced breast cancer: A multi-center retrospective analysis. *Anti-cancer drugs.* 2021.

17. Garly R, Berg T, Jensen MB, et al. A retrospective, non-interventional study of breast cancer patients diagnosed with ER+/HER2 negative, locally advanced or metastatic breast cancer treated with palbociclib in Denmark. *Acta Oncologica.* 2023;62(3):290-97.

18. Sharaf. B. From clinical trials to clinical practice; real world clinical outcomes of patients treated with ribociclib in combination with aromatase inhibitors or fulvestrant for HR positive, HER2- negative metastatic breast cancer. In:2023.

19. Singer. C. Real-world outcomes with first-line ribociclib + endocrine therapy in patients with metastatic HR+, HER2– breast cancer: Fourth interim analysis of REACH AUT trial. In:2023.

20. Jackisch C, Brucker C, Decker T, et al. Abstract P4-01-01: RIBANNA 5th interim analysis: Matched-pair analysis of progression-free survival (PFS) across treatment cohorts and comparison of frontline ribociclib + endocrine therapy PFS data from RIBANNA vs MONALEESA trials, in HR+, HER2– ABC. *Cancer Research.* 2023;83(5_Supplement):P4-01-01.

21. Del Re M, Crucitta S, Omarini C, et al. Concomitant administration of proton pump inhibitors does not significantly affect clinical outcomes in metastatic breast cancer patients treated with ribociclib. *Breast (Edinburgh, Scotland).* 2022;66:157-61.
